# Coordinated oral–gut microbiota relocation in connective tissue diseases: a systematic review

**DOI:** 10.3389/fimmu.2026.1841874

**Published:** 2026-07-03

**Authors:** Verena Ida Meyer, Sylvio Redanz, Martin Alexander Kriegel

**Affiliations:** 1 Department of Translational Rheumatology and Immunology, Institute of Musculoskeletal Medicine, University of Münster, Münster, Germany; 2 Section of Rheumatology and Clinical Immunology, Department of Internal Medicine, University Hospital Münster, Münster, Germany; 3 Cells in Motion Interfaculty Centre, University of Münster, Münster, Germany; 4 Department of Immunobiology, Yale University School of Medicine, New Haven, CT, United States

**Keywords:** connective tissue diseases, dysbiosis, gastrointestinal microbiome, microbiota, mouth, mucosal immunity, Sjögren´s disease, systemic lupus erythematosus

## Abstract

**Background:**

Alterations of the gut microbiome are well documented in connective tissue diseases, whereas the oral microbiome has largely been studied in isolation. Emerging evidence suggests coordinated dysbiosis across mucosal sites with oral-gut relocation of pathobionts occurring in animal models; however, it remains unclear whether consistent oral and gut microbiome alterations occur in systemic lupus erythematosus (SLE) and primary Sjögren’s syndrome (pSS). This review systematically synthesizes evidence on oral and gut microbiome alterations in SLE and pSS with a focus on recurrent opposing abundance patterns across anatomical sites compatible with oral–gut microbial relocation.

**Methods:**

Observational studies comparing adult patients with SLE or pSS to healthy controls and reporting oral and/or gut microbiome data were included. Interventional studies, case reports, reviews, and non-human studies were excluded. PubMed was searched from inception to November 2024. Study quality was assessed using the Newcastle–Ottawa Scale. Microbial alterations were harmonized using current NCBI taxonomy and synthesized descriptively without meta-analysis.

**Results:**

Thirty-three studies comprising 1,385 patients and 2,131 healthy controls were included. Intestinal Shannon and Simpson α-diversity were frequently reduced, whereas oral diversity was preserved or increased. Recurrent opposing abundance patterns were observed for specific taxa, most consistently involving *Streptococcus* and Actinomycetota in SLE and Pseudomonadota in pSS, characterized by decreased oral and increased intestinal relative abundance. Several taxa, including *Veillonella* and *Veillonellaceae*, showed parallel enrichment across both sites.

**Discussion:**

SLE and pSS are characterized by coordinated dysregulation of the oral and gut microbiomes. Opposing abundance patterns across anatomical sites support the concept of disease-associated microbial redistribution although causal inference is limited given the data was derived primarily from cross-sectional studies with relative abundances. Overall, this study highlights the oral–gut axis as an underexplored dimension of mucosal immune dysregulation in connective tissue diseases.

## 1 Introduction

The human microbiome plays a central role in host physiology and immune homeostasis. Microbial communities exhibit strong niche specialization across different anatomical sites (1, 2), with the gut microbiome representing the most complex ecosystem and the oral cavity harbouring the second most diverse microbial community in the human body (1).

The oral microbiome forms a critical interface between the host and the external environment and contributes to mucosal immune regulation (2). It is composed of site-specific microbial communities (3) dominated by genera such as *Streptococcus*, *Veillonella*, *Neisseria* and *Prevotella* (4, 5). Although relatively stable under physiological conditions, the oral microbiome is susceptible to inflammatory and immunological stressors (6).

Autoimmune diseases arise from dysregulation of immunoregulatory mechanisms and are consistently associated with alterations of the gut microbiome (7, 8). These alterations often overlap across different autoimmune conditions, suggesting shared pathogenic mechanisms rather than disease-specific microbial signatures (9–11). Connective tissue diseases represent a heterogeneous subgroup of autoimmune disorders characterized by chronic inflammation and multi-organ involvement. Among these, systemic lupus erythematosus (SLE) and primary Sjögren’s syndrome (pSS, recently renamed Sjögren's disease) are of particular interest due to their systemic immune activation and frequent mucosal manifestations (12–16). In both diseases, microbiome alterations have been reported predominantly in the gut, while oral microbiome changes have received comparatively less attention. We have previously profiled oral, gut, and skin microbiomes of SLE patients (17) and identified in the buccal mucosa *Actinomyces massiliensis* as a Ro60 ortholog-containing bacterium that could represent an oral trigger of human Ro60 autoimmunity. Chen et al. (18) demonstrated oral-to-gut relocation of A. massiliensis in untreated SLE patients, which prompted this systematic review.

An increasingly discussed concept linking oral and systemic disease is the oral–gut microbiome axis (19). This hypothesis proposes that orally associated bacteria may act as pathobionts of non-communicable diseases when translocated to the gastrointestinal tract, particularly under conditions of oral dysbiosis, impaired intestinal barrier function, or altered immune surveillance (20–24). Swallowed oral bacteria may survive gastrointestinal transit and contribute to immune dysregulation in the gut (23, 25, 26). Indeed, greater disruption of the oral microbiome has been associated with increased overlap between oral and gut bacterial taxa (21), and orally associated genera such as *Streptococcus* (21, 27), *Haemophilus* (27, 28) and *Klebsiella* (22) have been implicated in this context.

Despite growing evidence for microbiome involvement in connective tissue diseases, most studies continue to investigate the oral and gut microbiomes in isolation. Whether consistent, coordinated microbial alterations occur across these anatomical sites remains insufficiently explored. Recurrent opposing abundance patterns, characterized by decreased oral and increased intestinal relative abundance of specific taxa would be compatible with an oral–gut microbiome axis but have not been systematically synthesized. This review systematically synthesizes evidence on coordinated oral and gut microbiome alterations in SLE and pSS, with a focus on recurrent opposing abundance patterns across anatomical sites. Additionally, the influence of factors such as medications (proton pump inhibitors (PPI), corticosteroids) was investigated. Overall, the aim of this study was to understand potential interactions between the oral and gut microbiomes with associations identified to provide insights on mechanisms such as oral-gut relocation and impact on mucosal and systemic immunity disturbed in connective tissue diseases.

## 2 Materials and methods

This systematic review was conducted in accordance with the PRISMA 2020 guidelines for Systematic Reviews.

### 2.1 Information sources

A systematic literature search was conducted using the PubMed database. PubMed was selected as the primary information source due to its comprehensive coverage of biomedical and immunological literature. Taxonomic verifications of the identified microbial taxa were performed using the NCBI taxonomy browser to ensure consistency with current nomenclature. BioRender was used for visual representations.

### 2.2 Preliminary literature assessment

Previously published meta-analyses and systematic reviews addressing the oral-gut microbiome axis in connective tissue diseases were screened in PubMed database from inception date to November 1^st^, 2024. The following search strategy was applied: [Microbiota] MeSH AND [Connective Tissue Diseases] MeSH, article type: Meta-Analysis or Systematic Review. This yielded 19 results, of which 12 were considered relevant to the topic. Only three publications directly addressed both oral and gut microbiome, of which one addressed RA (29), one pSS (30) and one SLE (31). The remainder exclusively focused on the gut and did not investigate oral-gut interactions.

Accordingly, this review specifically emphasizes the oral-gut connection to provide a new perspective going beyond isolated microbiota studies. Additionally, by including more than one connective tissue disease (CTD), the aim was to provide a more comprehensive understanding of potential oral-gut microbiota shifts across CTDs. The following disease states were included: pSS (also called Sjögren's disease), SLE, idiopathic inflammatory myositis/dematomyositis, and systemic sclerosis.

### 2.3 Selection process

A systematic search of PubMed was conducted from inception date to November 10^th^, 2024. The search strategy was as follows:

[Systemic Lupus Erythematosus] MeSH AND [Microbiota] MeSH → 167 results

[Systemic Lupus Erythematosus] MeSH AND [Microbiota] MeSH AND [Mouth] MeSH → 9 results

[Sjögren´s syndrome] MeSH AND [Microbiota] MeSH → 59 results

[Sjögren´s syndrome] MeSH AND [Microbiota] MeSH AND [Mouth] MeSH → 11 results

[Scleroderma, Systemic] MeSH AND [Microbiota] MeSH → 41 results

[Scleroderma, Systemic] MeSH AND [Microbiota] MeSH AND [MOUTH] MeSH → 1 result

[Myositis] MeSH AND [Microbiota] MeSH → 9 results

[Myositis] MeSH AND [Microbiota] MeSH AND [MOUTH] MeSH → 1 result

Article type: no exceptions

Due to the limited number of oral microbiome studies in scleroderma and myositis, the final analysis focused on SLE and pSS.

**Figure 1:**
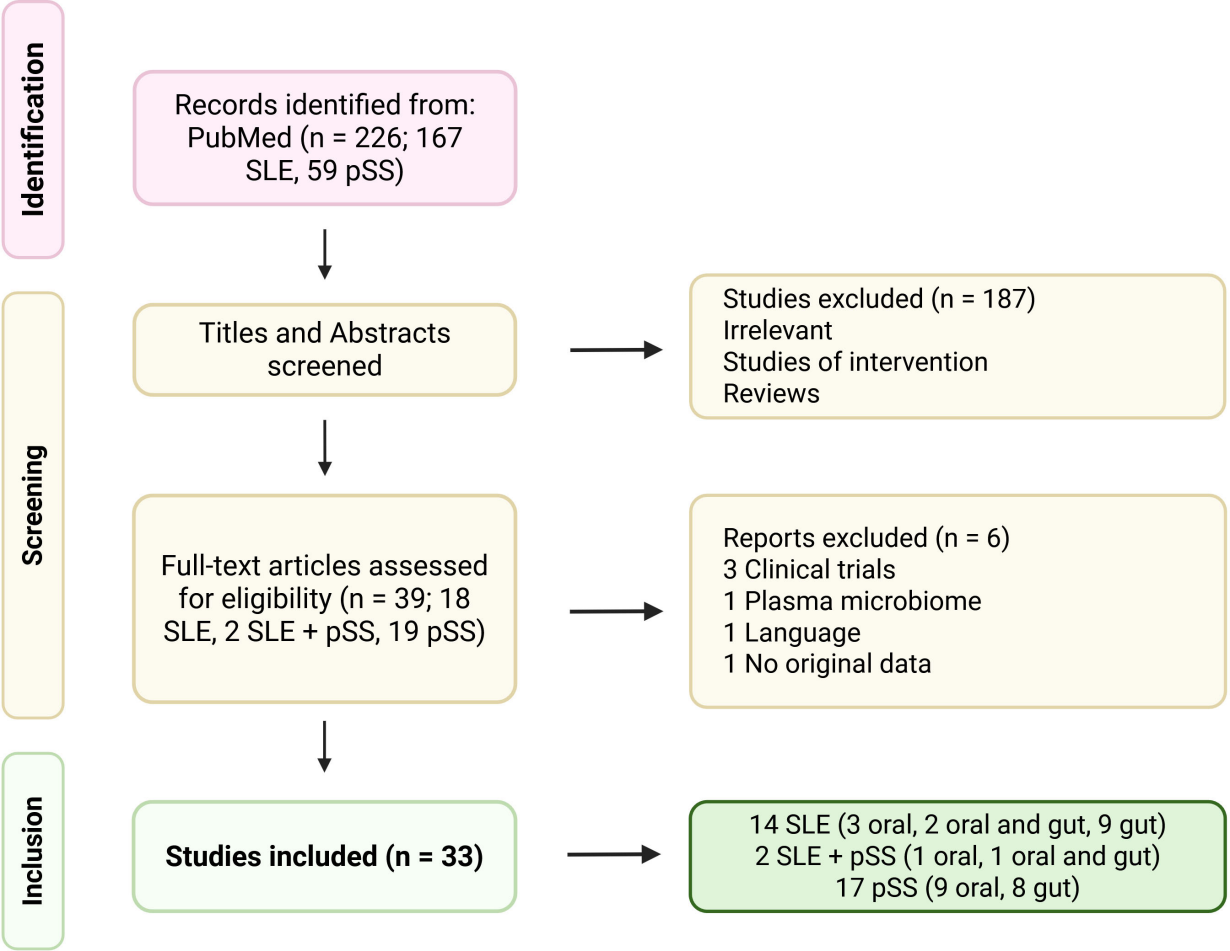
Study selection flowchart. Flowchart illustrating the systematic literature search and study selection process for systemic lupus erythematosus (SLE) and primary Sjögren’s syndrome (pSS). Records were identified through PubMed database searches, screened for eligibility, and assessed by full-text review. Reasons for exclusion at each stage are indicated. Created in BioRender.com.

The selection process is illustrated in **Figure 1**. Title and abstract of the studies were screened with inclusion criteria as follows: (1) Comparative analysis between patients and healthy controls (HC) with both being ≥18 years of age, (2) Observational design (no interventional studies, case reports, editorials or narrative reviews) and original research (no review or meta-analysis), (3) Relevance for objective with data on gut and/or oral microbiota, (4) Full text available in English. Title and abstract screening as well as full-text eligibility assessment were performed by a single reviewer.

### 2.4 Data extraction

For each study, the following information was extracted:

- MeSH terms, title, PMID, author, year, journal
- Microbiome sampling sites (oral/gut)
- Demographics: gender distribution, sample size, age, ethnicity, geography (**Supplementary Table 1**)
- Sample type, matching variables
- Medication (PPI, cortico steroids, xerogenic medications)
- Study design and inclusion criteria
- Microbiome sequencing technology, taxonomic and statistical analysis methods
- Potential biomarkers, key findings as reported in the abstract and conclusion and additional functional and clinical information (e.g., microbial pathway analysis, immunological markers, correlation with disease activity)
- Microbial diversity metrics (α-, β-diversity)
- Relative abundance data (phylum, family, genus, species level)
- Firmicutes/Bacteroidetes ratio
- Abundance of recurrently reported taxa (*Faecalibacterium prausnitzii* and *Mediterraneibacter gnavus)*
- Availability of raw sequencing data in public repositories
- Potential biases (assessed using the Newcastle–Ottawa Scale)

Compiled information of all studies can be found in the **Supplementary Data Sheet 1**. Missing information was requested from the corresponding authors by e-mail. A list of contacted authors and responses can also be found in the **Supplementary Table 2**. Potential biases were assessed using the New Castle Ottawa Scale for cross sectional studies (**Supplementary Table 3**). Absolute bacterial abundances were not available in most studies and thus excluded from the extraction table.

### 2.5 Synthesis and visualization

Two types of synthesis tables were compiled. First, a table summarizing relative abundance changes of microbial taxa (oral vs. gut microbiome) on phylum, family, genus and species level (**Supplementary Data Sheet 2**). All comparisons were made between patients and HC. Differences in microbial relative abundance were categorized as statistically significant (p < 0.05), non-significant trends (increase/decrease) or no observable difference. Presumptions at higher taxonomic levels were considered valid if they were supported by at least two independent study results (p < 0.05) at that level. However, if one of the supporting findings represented a presumption derived from consistent results at a lower taxonomic level rather than a direct study result, at least three supporting studies were required. In cases of conflicting evidence, a presumption was considered valid only if at least twice as many studies supported one direction compared with the opposing direction.

Secondly, a medication-related relative abundance table (**Supplementary Data Sheets 3**, **4**) was compiled, comparing microbial profiles between patients receiving certain medications known to influence gastric mucosal barriers (PPI/corticosteroids) and those not receiving them. When available, information on medication dosage and the proportion of treated patients was included. Although NSAIDs are also well known to affect mucosal integrity, the available data were not sufficiently consistent or detailed to justify a separate evaluation of their potential impact.

Furthermore, taxonomic harmonization was performed by mapping all reported taxa to the current NCBI taxonomy as of July 2025. Outdated taxonomic terms had to be reassigned to the correct phylum, family, genus, or species level. A full list of adjustments is provided in the **Supplementary Tables 4–7**.

## 3 Results

### 3.1 Study characteristics

During full text screening including supplementary materials, six studies (32–37) were removed based on the inclusion criteria, which resulted in a total of 33 included studies (18, 38–69) (**Figure 1**). Their publication date ranged from 2014 (45) to 2024 (51, 67) with most studies published in 2020 (n = 6) (38, 41, 48, 56–58) and 2023 (n = 8) (40, 43, 44, 46, 52, 55, 59, 64).

In total, 1,385 patients (828 SLE and 557 pSS) and 2,131 HC were included in the analysis. Sample size ranged from nine (69) to 225 patients (44) per study. The high number of HC was largely driven by van der Meulen et al. (63), having published a large community-based control group (965 HC) and fewer patients (30 SLE, 39 pSS).

Overall, approximately one-third of the studies matched age and sex between patients and HC. A lack of matched controls occurred mostly in pSS studies (n = 5) (56, 57, 62, 66, 67) (**Supplementary Figure 1**).

Most patients were female with an average F:M ratio of 10.1:1 similar across SLE and pSS. HC showed a lower female predominance of 2.9:1. Sex distribution was not reported in three SLE studies (46, 48, 60). Six SLE and eight pSS studies included 100% female patient cohorts (**Supplementary Figure 2**).

The mean age was lower in SLE patients (39.61 years) than in pSS patients (53.16 years). The age of HC were in average 37.84 years (SLE studies) and 46.88 years (pSS studies) although several studies lacked HC age-related data (39, 40, 46, 58, 60) (**Supplementary Figure 3**).

Most studies (60%) recruited participants from Asia (China, India and South Korea) with the remainder from Europe or America, resulting in predominantly Asian cohorts (**Supplementary Table 1**).

**Figure 2:**
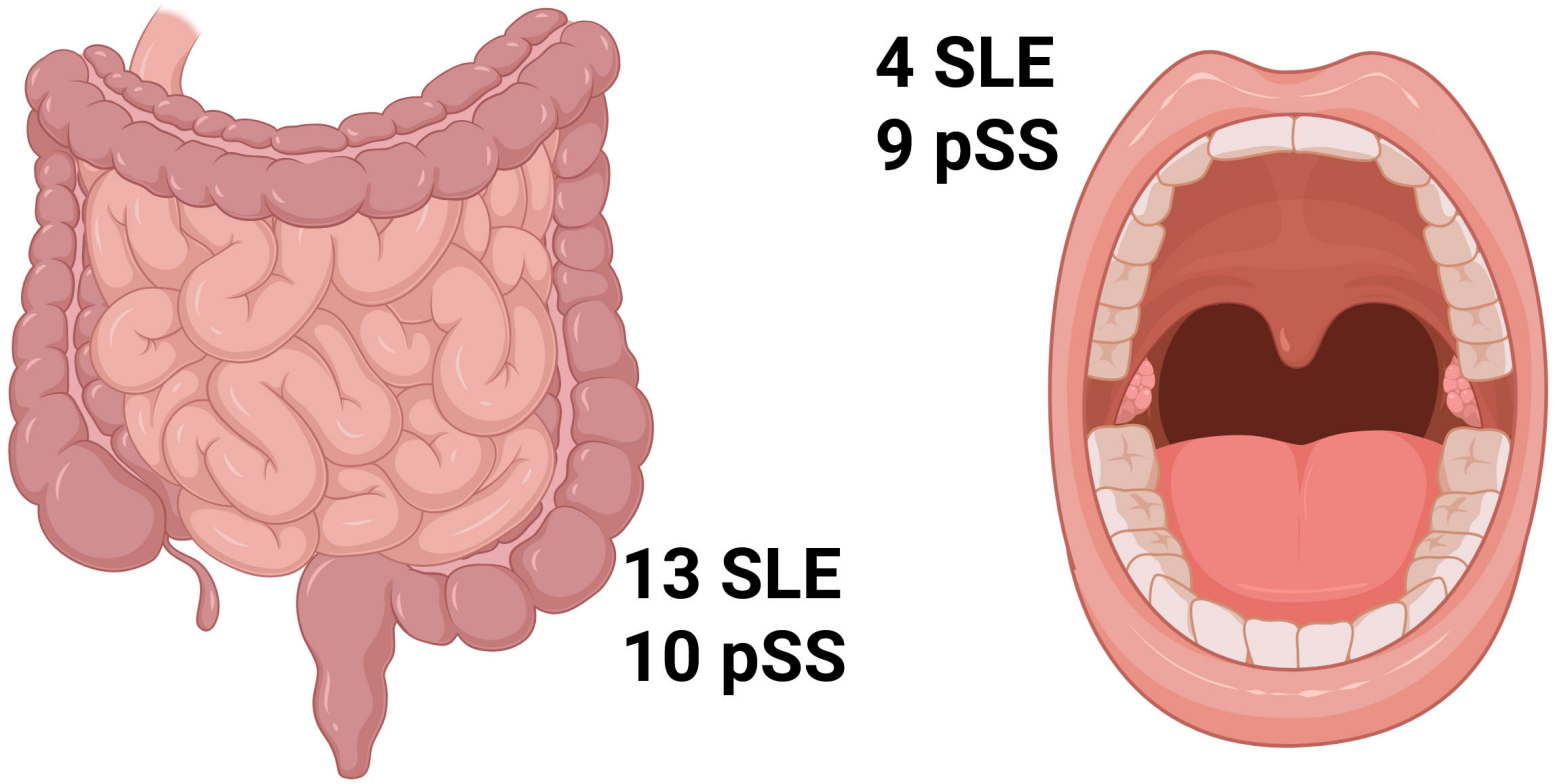
Anatomical location of microbiome sampling. Distribution of sampling sites across included studies. Oral samples included saliva, subgingival plaque, supragingival plaque, and oral swabs, while gut samples consist exclusively of stool specimens. Numbers indicate the total count of studies per disease group and sampling location. Created in BioRender.com.

Among the included studies, 14 investigated SLE (three oral, two oral and gut - one of them only reporting explicitly gut data (18) -and nine gut only) and 17 pSS studies (nine oral, eight gut). Two addressed both diseases (one gut (46) and one oral and gut - yet only with complete data on the gut microbiome (63). In total, 13 studies investigated the oral (four SLE, nine pSS) and 23 the gut microbiome (13 SLE, 10 pSS) (**Figure 2**).

**Figure 3:**
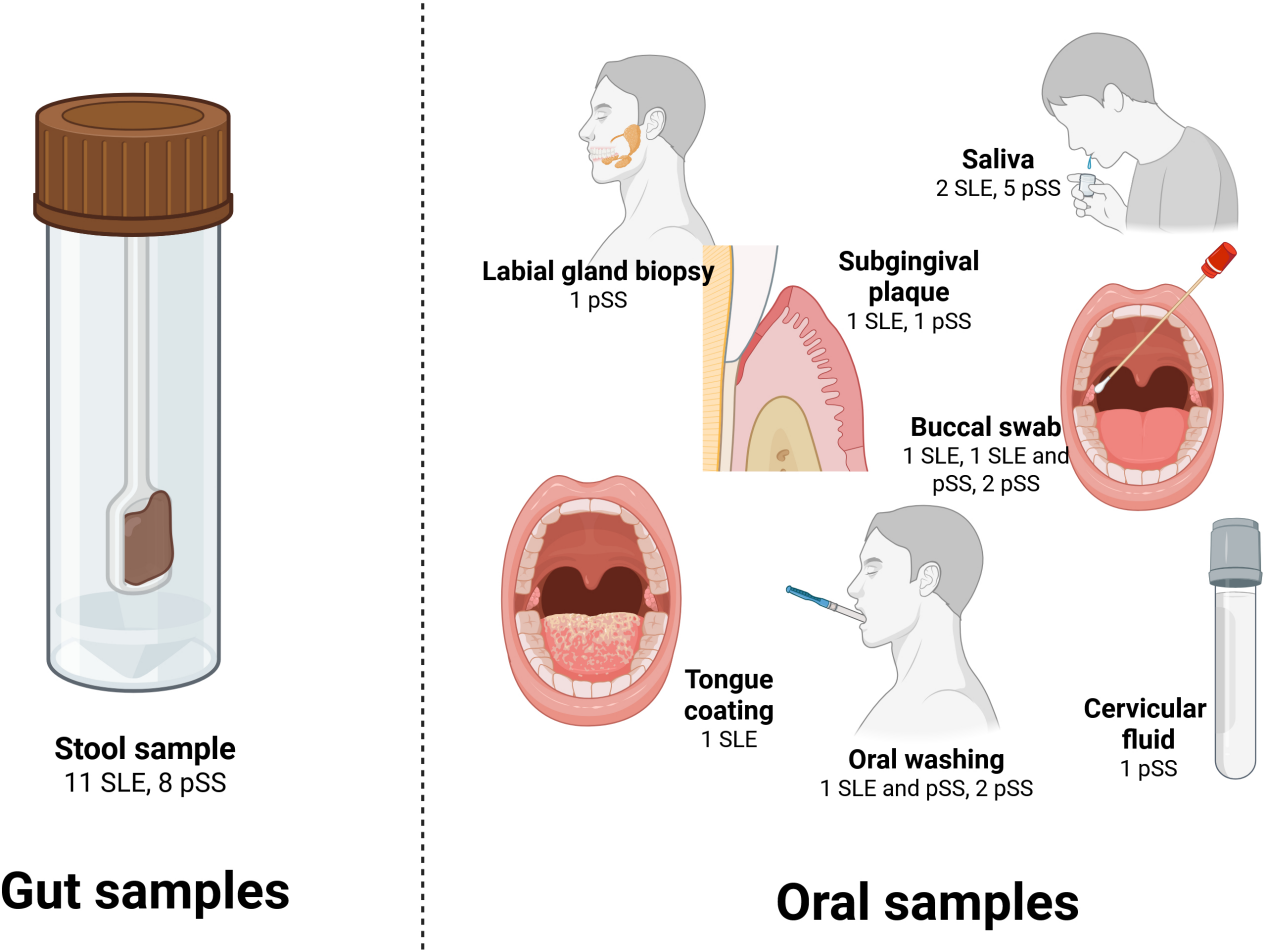
Types of biological samples analysed. Overview of biological sample types used for oral and gut microbiome analyses in the included studies. Gut microbiome investigations uniformly relied on stool samples, whereas oral microbiome studies employed heterogeneous sampling methods. Created in BioRender.com.

There was an expected imbalance in the variety of types of oral samples. While gut studies exclusively used stool samples, oral sampling methods differed widely including saliva and plaque-based samples (**Figure 3**). A comprehensive overview on the sampling methods can be found in the **Supplementary Data Sheet 1**.

**Figure 4:**
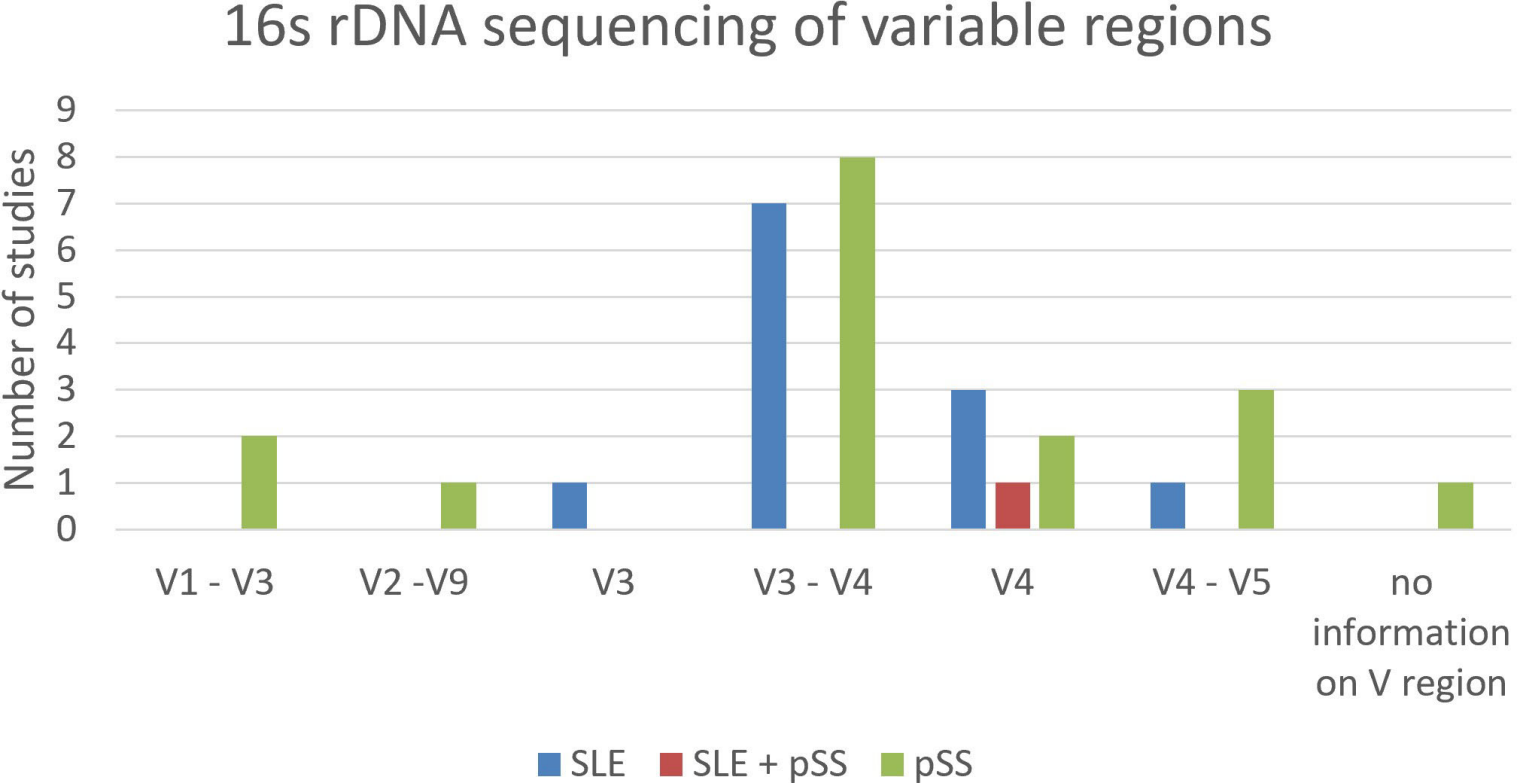
16S rDNA sequencing regions. Targeted hypervariable regions of the 16S rRNA gene used for microbiome profiling across studies. The V3–V4 region was most frequently sequenced, followed by other regions or combinations thereof. Bars indicate the number of studies per region and disease group. Created in Microsoft Word.

Most studies applied 16S rRNA gene sequencing, mainly targeting the V3 – V4 region (n = 15) with other regions used less frequently (**Figure 4**).

Three studies employed alternative approaches including shotgun metagenome sequencing (18, 46) and deep RNA sequencing (43).

Regarding taxonomic processing, 22 studies (10 SLE, 12 pSS) used operational taxonomic unit (OTU)-based clustering (mostly 97% similarity threshold) and seven (5 SLE and 2 pSS) used amplicon sequence variant (ASV)-based analyses. A hybrid approach was applied in two studies of Azzouz et al. (39, 40).

### 3.2 Quality assessment

**Figure 5:**
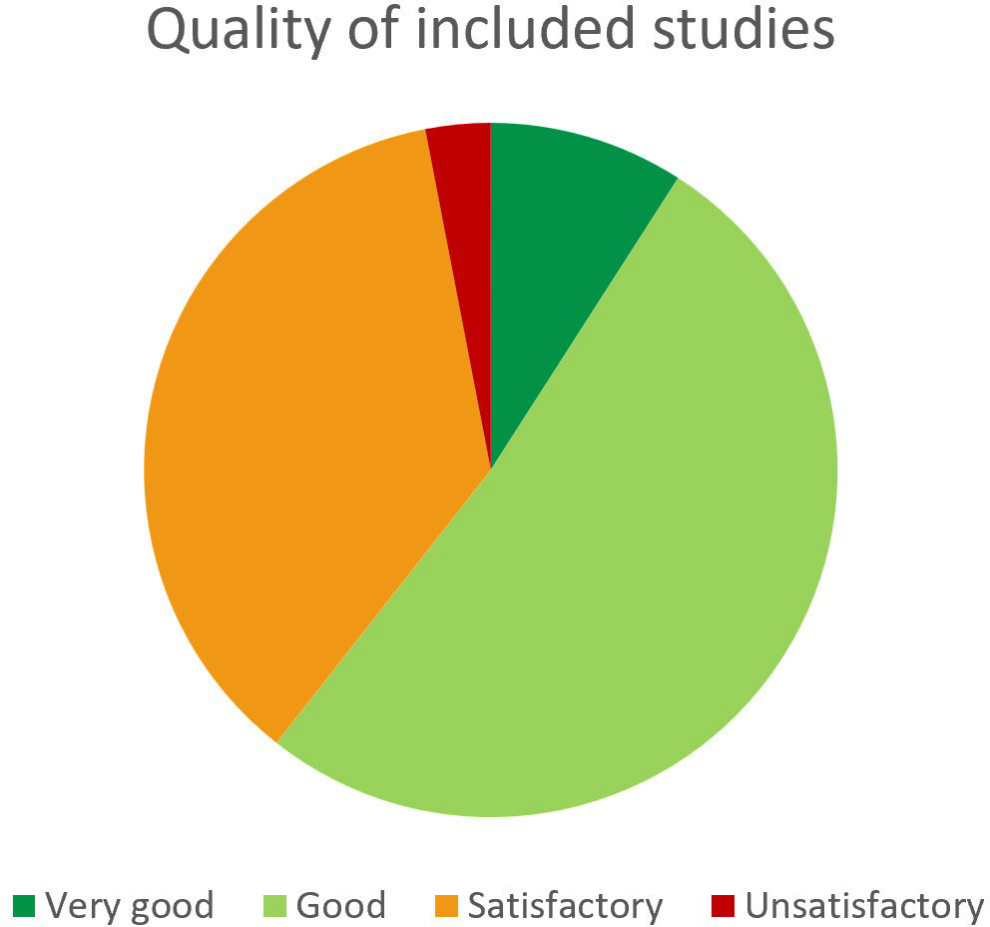
Quality assessment of included studies. Methodological quality of included cross-sectional studies assessed using the Newcastle–Ottawa Scale. Studies are categorized as very good (9–10 points), good (7–8 points), satisfactory (5–6 points), or unsatisfactory (≤4 points). Distributions are shown for all included studies. Created in Microsoft Word

The Newcastle-Ottawa scale for Cross sectional studies was used for assessment of methodological quality. The scale evaluates three domains: selection, comparability and outcome, with a maximum score of 10 points (70). Collectively, there was a balanced distribution for pSS and SLE studies rated as good or satisfactory (**Figure 5**). Three studies were rated as very good (42, 44, 53), while one study was rated unsatisfactory (39).

Individual data for each study as well as structured assessments of study design, representativeness and data extraction can be found in the **Supplementary Data Sheet 1**. For pSS patients, individuals with sicca symptoms, who did not meet classification criteria for pSS were frequently used as controls instead of HC (38, 47, 56, 57, 61). However, only comparisons between patients and HC were included in the relative abundance analysis. Accordingly Kim et al. (47) was excluded from the comparative relative abundance table.

### 3.3 Diversity metrics

The hypothesis proposed by Hevia et al. (45) of a lower Bacillota/Bacteroidota ratio (outdated taxonomy: Firmicutes/Bacteroides ratio) in connective tissue diseases, especially SLE, was examined in the included studies. Only limited data regarding this ratio were available, mostly regarding the gut. Toumi et al. reported a lower Bacillota/Bacteroidota ratio in SLE especially influenced by higher disease activity (SLEDAI) (60). However, the available evidence was insufficient to identify consistent associations across studies.

β-diversity analyses were mostly assessed using principal coordinate analysis (PCoA). High β-diversity between patients and HC was observed in both connective tissue diseases in most cases (29/33 studies), corresponding to a distinct separation of microbial community structures. This pattern was observed in both gut and oral microbiome studies.

**Figure 6:**
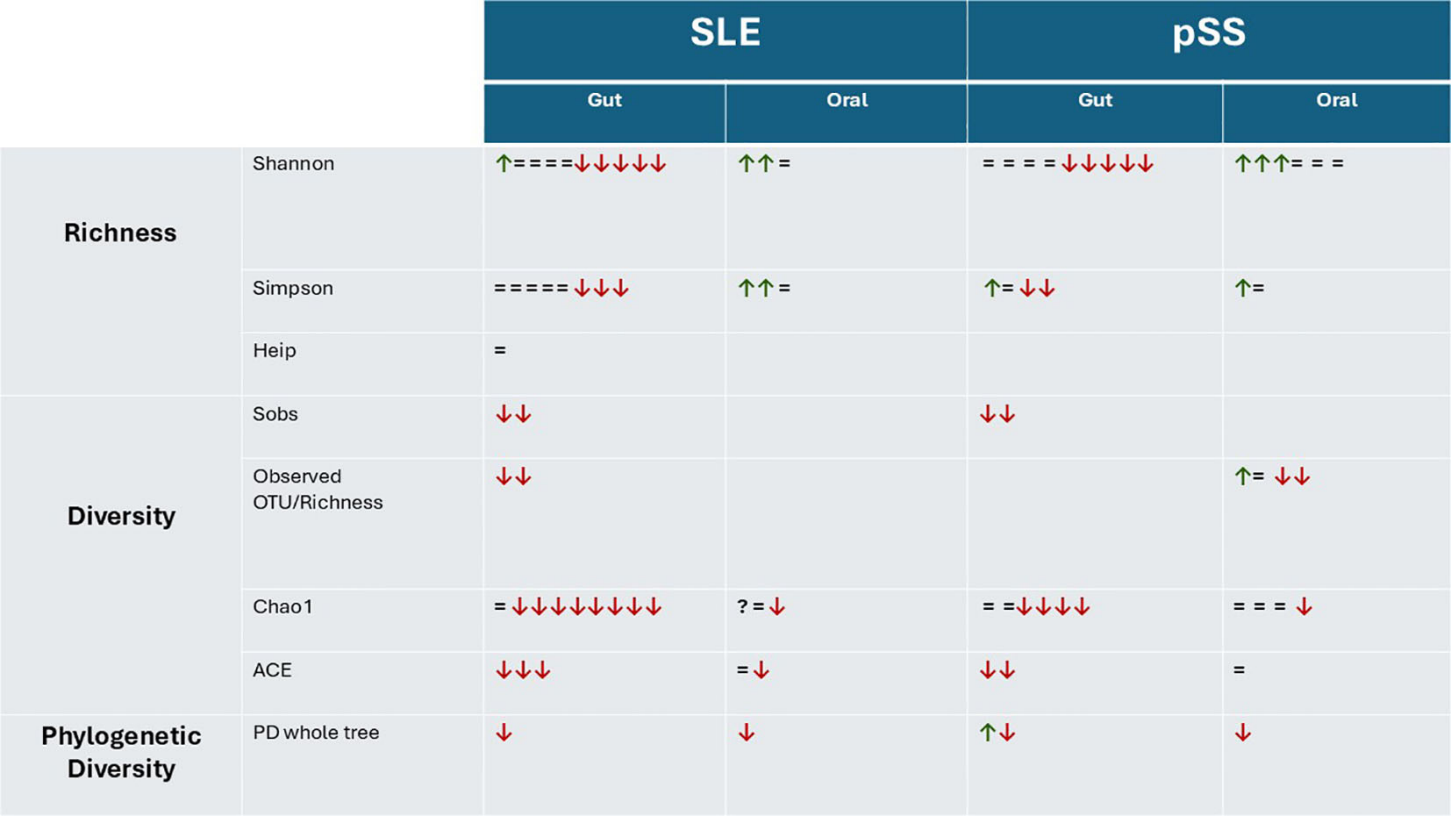
α-diversity alterations in oral and gut microbiomes. Significant changes in α-diversity indices comparing patients with connective tissue diseases to healthy controls. Only statistically significant results are displayed. Decreases in richness and diversity are predominantly observed in the gut microbiome, whereas increased diversity indices are more frequently reported in the oral microbiome. Symbols indicate the respective α-diversity metrics (e.g., Shannon, Simpson, Sobs (Species observed), Chao1, Abundance-based Coverage Estimator (ACE), Phylogenetic Diversity (PD) whole tree). The question mark denotes conflicting findings depending on periodontal status as reported by Corrêa et al. (43) Created in Microsoft PowerPoint.

Overall, the α-diversity indices reflecting microbial richness were more often reported as being reduced in patients than in HCs in both the gut and the oral microbiomes, respectively. Richness estimators most frequently reported included Chao1 and observed features. When examining the diversity or evenness α-indices, contrasting patterns between oral and gut microbiome were found. Several studies reported higher Shannon and Simpson indices in oral samples from patients, whereas reduced values were more commonly reported in gut samples of patients. These opposing trends were observed both within and across studies (**Figure 6**).

In cohort-internal analyses, Liu et al. reported reduced faecal Shannon and Simpson indices together with increased oral indices in the same SLE patient cohort (n = 35) (53). Jia et al. found a decrease in Shannon diversity in both pSS as well as SLE faeces, yet the pSS decrease was more significant (46). In contrast, one study reported an increased Simpson index in the gut of pSS patients (66).

### 3.4 Recurrent oral-gut abundance patterns

Original data from included publications as well as derived presumptions, defined as higher-level taxonomic inference based on at least two consistent findings at lower taxonomic level, were included in the relative abundance table (**Supplementary Data Sheet 2**). Absolute abundance data was requested from the corresponding authors but was insufficient for inclusion (**Supplementary Data Sheet 1**). The relative abundance data of Kim et al. were excluded due to comparisons of pSS patients with control subjects with similar symptoms (non-SS sicca symptoms) (47); for Wu et al., only pre-treatment data was included, to ensure comparability as patients received an experimental herbal formulation (66). The remaining relative abundance data were predominantly available at the genus level (28/33 studies) as shown in **Supplementary Figure 4**. The ratio of oral/gut studies in pSS studies was more balanced than in SLE with comparatively fewer oral studies available for SLE.

**Figure 7:**
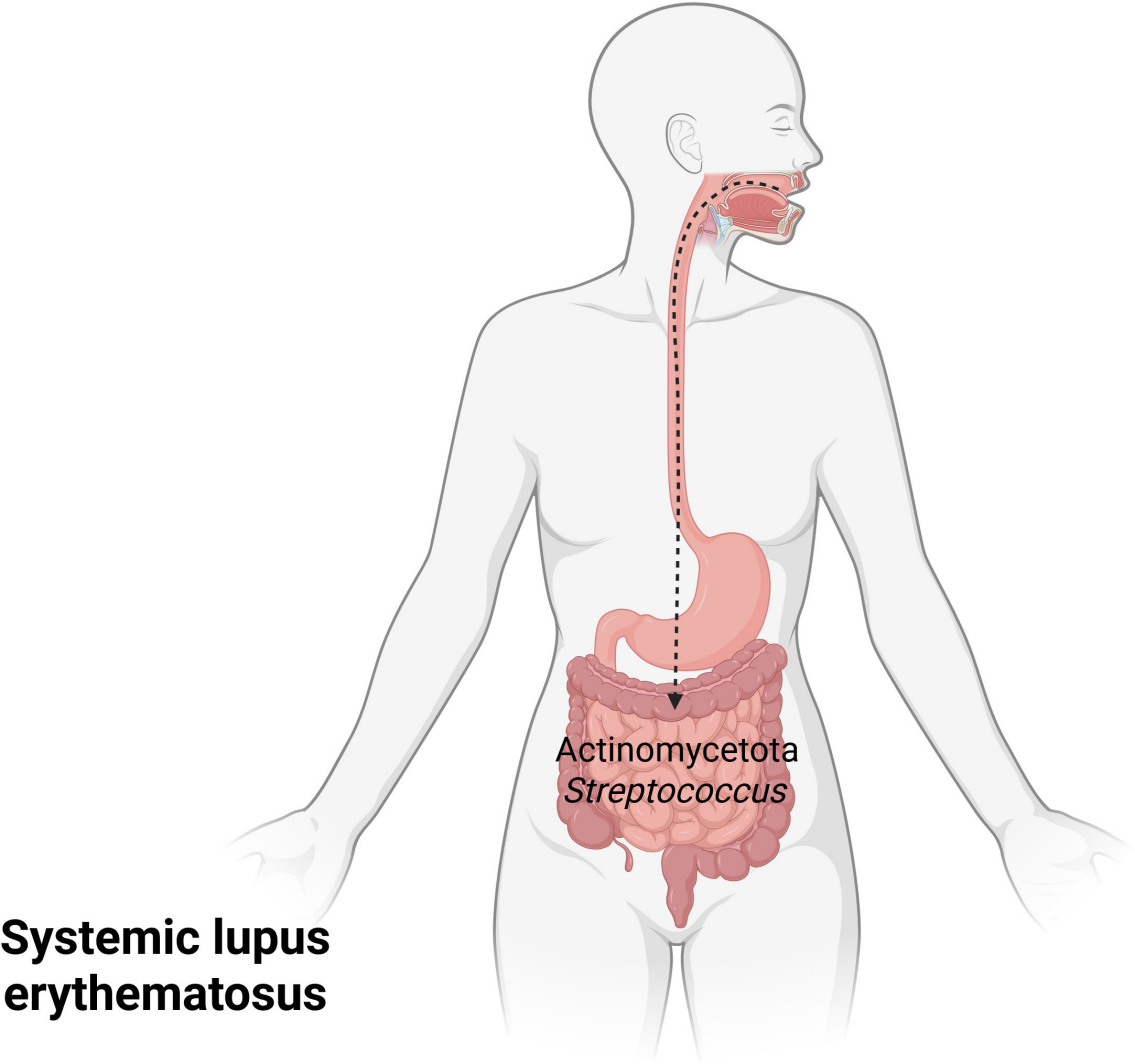
Criteria fulfilling oral–gut microbial changes in systemic lupus erythematosus. Schematic illustration summarizing bacterial taxa showing consistent, opposing relative abundance patterns between the oral cavity and the gut in systemic lupus erythematosus (SLE). Actinomycetota and *Streptococcus* demonstrate decreased relative abundance in the oral microbiome and increased relative abundance in the gut across multiple independent studies, fulfilling the predefined criteria for hypothesis-confirming taxa. These findings support the concept of a potential oral–gut microbial relocation axis in SLE. Created with BioRender.com.

**Figure 8:**
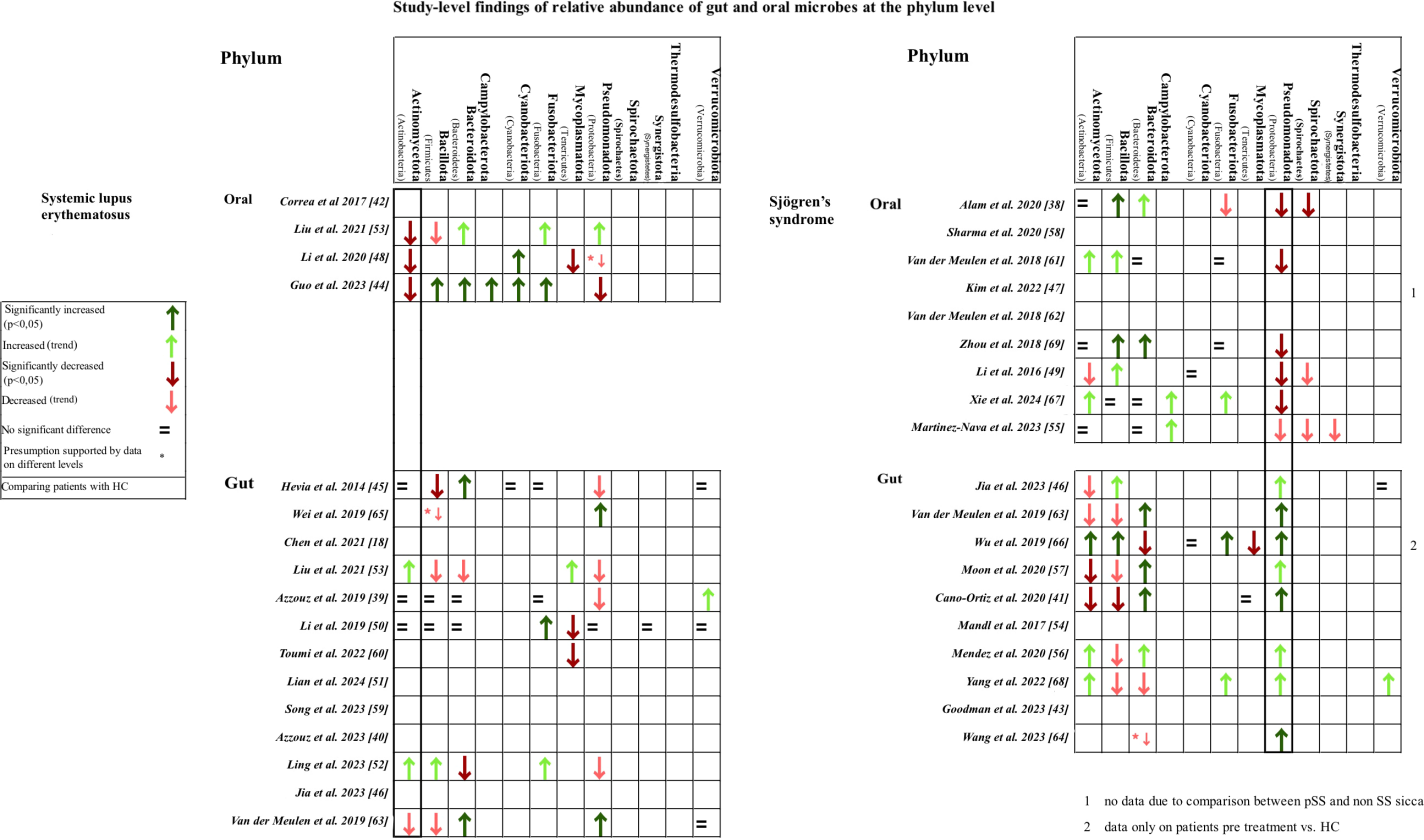
Relative abundance changes of bacterial phyla across oral and gut microbiome studies. Matrix summarizing relative abundance changes of bacterial phyla in patients with systemic lupus erythematosus (SLE) and primary Sjögren’s syndrome (pSS) compared with healthy controls. Oral and gut microbiome studies are displayed separately. Green arrows (dark = significant, light = trends) indicate increased relative abundance, red arrows (dark = significant, light = trends) indicate decreased relative abundance, and black equal signs denote no statistically significant difference. Empty fields indicate taxa not reported in the respective study. Stars (*) denote presumptions based on underlying taxonomic levels, supported by at least two independent significant study results. Only relative abundance data were included; absolute abundance data was not available. Taxonomic nomenclature was harmonized according to current NCBI taxonomy. Bold colums represent main findings on phylum level. Created in Microsoft Excel.

Hypothesis-confirming taxa were defined when supported by ≥2 intestinal and ≥2 oral studies, with ≤1 opposing result. Using these criteria, confirmed oral-gut relocating taxa for SLE were Actinomycetota with 3x oral decrease and 2x intestinal increase accompanied by one opposing result and three studies reporting no significant differences. *Streptococcus* fulfilled the criteria with 3x oral decrease and 7x intestinal increase and no opposing results (**Figures 7**, **8**).

**Figure 9:**
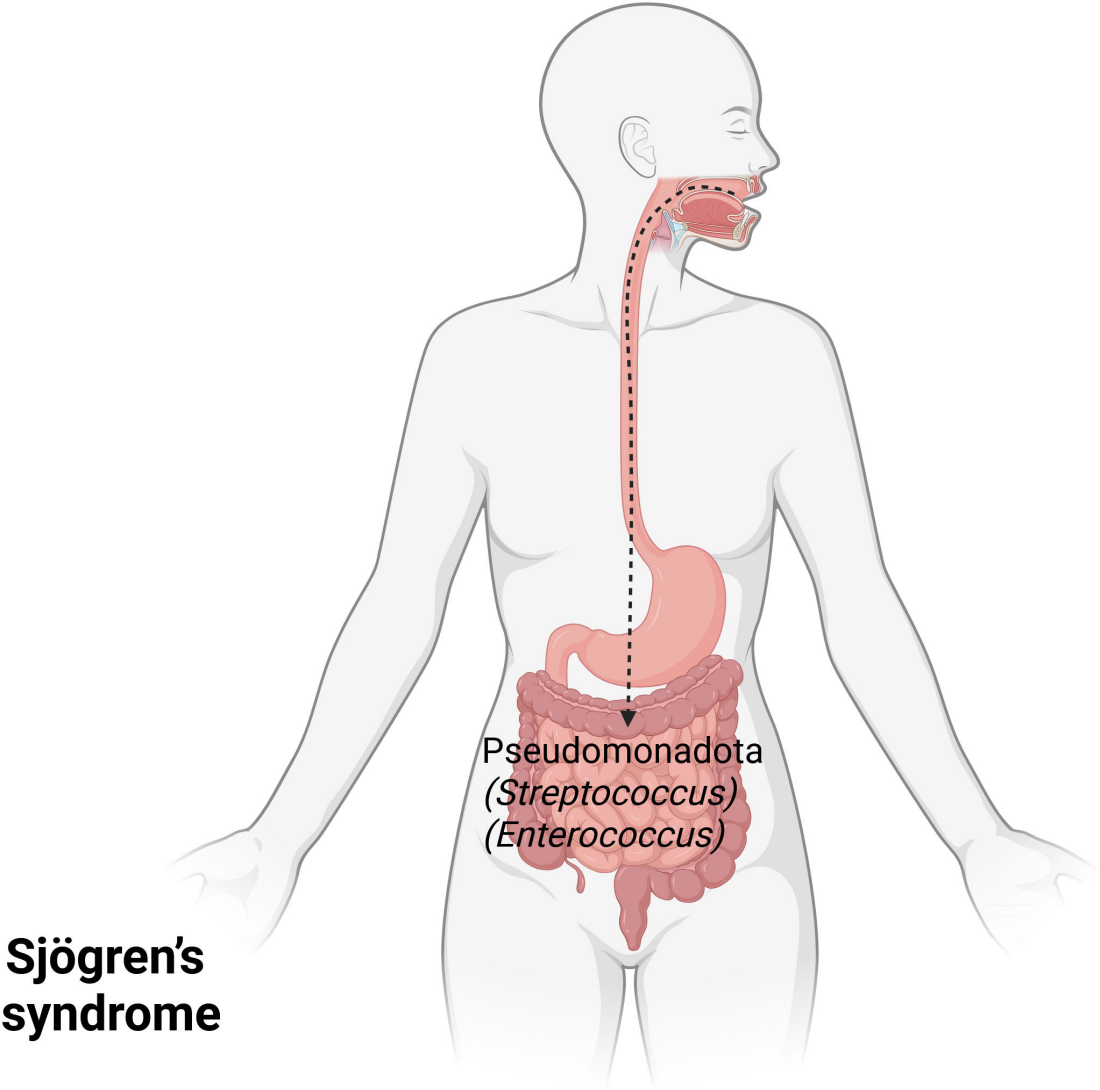
Criteria fulfilling oral–gut microbial changes in primary Sjögren’s syndrome. Schematic representation of bacterial taxa exhibiting consistent oral–gut abundance patterns in primary Sjögren’s syndrome (pSS). The phylum Pseudomonadota shows reduced relative abundance in the oral microbiome and increased relative abundance in the gut across included studies, meeting the criteria for hypothesis-confirming oral–gut microbial shifts. Similar trends were observed with *Streptococcus* and *Enterococcus* on the genus level. Created with BioRender.com.

Confirmed taxa for pSS were Pseudomonadota with 6x oral decrease and 8x intestinal increase without opposing results (**Figures 8**, **9**).

These consistently opposing relative abundance patterns between the oral and gut microbiomes support coordinated oral–gut microbiome alterations. Especially *Streptococcus* in SLE and Pseudomonadota in pSS exhibited the most consistent oral-gut relocation across anatomical sites.

### 3.5 Bacterial taxa with disease-specific changes

In addition to the main findings, several disease specific microbial alterations were observed across both anatomical sites as well as at individual locations (**Tables 1**, **2**).

**Table 1:** Microbial alteration trends observed concurrently in oral and gut microbiomes.

Disease	Taxonomy	Oral↑, gut↓	Oral↓, gut↑	Oral and gut↑	Oral and gut↓
SLE	phylum		Actinomycetota	Fusobacteriota	Mycoplasmatota
	family		Streptococcaceae	**Veillonellaceae**	
	genus	*Prevotella*	***Streptococcus***	*Blautia* *Lactobacillus* ***Veillonella*** *Leptotrichia* *Fusobacterium*	
	species		*Corynebacterium durum* *Rothia aeria* *Streptococcus anginosus* *Dialister invisus*	*Limosilactobacillus mucosae*	
pSS	phylum	Bacillota	Pseudomonadota	Bacteroidota	
	family			Coriobacteriaceae Lactobacillaceae **Veillonellaceae**	
	genus	*Actinomyces* *Bifidobacterium*	*Enterococcus* ***Streptococcus***	*Limosilactobacillus* ***Veillonella*** *Prevotella*	*Ruminococcus*
	species			*Streptococcus anginosus* *Streptococcus mutans* *Ligilactobacillus salivarius*	

Summary of bacterial taxa showing relative abundance changes detected simultaneously in the oral cavity and the gut of patients with systemic lupus erythematosus (SLE) and primary Sjögren’s syndrome (pSS) compared with healthy controls.

Arrows indicate the direction of relative abundance change in patients (↑ increase, ↓ decrease). Grey shading highlights taxa exhibiting opposing abundance patterns between oral and gut microbiomes consistent with a potential oral–gut microbial relocation. Bold font indicates microbial alterations observed in both diseases. Only relative abundance data were included. Created in Microsoft Word.

**Table 2:** Microbial alteration trends observed in a single anatomical location.

Disease	Taxonomy	Oral↑	Oral↓	Gut↑	Gut↓
SLE	phylum	Bacteroidota Cyanobacteriota			Pseudomonadota
	family		Micrococcaceae Peptostreptococcaceae Halomonadaceae		**Lachnospiraceae** **Oscillospiraceae**
	genus		*Rothia* ***Granulicatella*** *Halomonas* ***Haemophilus***	***Megasphera*** *Alistipes*	*Bifidobacterium* *Phascolarctobacterium* *Roseburia* ***Faecalibacterium*** *Megamonas*
	species	*Hoylesella pleuritidis*		*Mediterraneibacter gnavus* *Intestinibacter bartlettii* *Veillonella dispar* *Bacteroides fragilis*	*Roseburia intestinalis* ***Faecalibacterium prausnitzii*** *Ruminococcus callidus*
pSS	phylum	Campylobacterota	Spirochaetota	Fusobacteriota	
	family		Burkholderiaceae Neisseriaceae Pasteurellaceae	Enterobacteriaceae	**Lachnospiraceae** **Oscillospiraceae** Porphyromonadaceae
	genus	*Atopobium* *Peptostreptococcus* *Dialister* *Fusobacterium*	*Abiotrophia* ***Granulicatella*** *Neisseria* ***Haemophilus***	*Lactobacillus* ***Megasphera*** *Bacteroides*	*Collinsella* *Eubacterium* *Blautia* *Dorea* ***Faecalibacterium*** *Alistipes*
	species		*Neisseria perflava* *Haemophilus parainfluenzae*	*Bifidobacterium dentium* *Escherichia coli*	***Faecalibacterium prausnitzii***

Summary of bacterial taxa showing relative abundance changes detected in a single anatomical location (oral cavity/gut) of patients with systemic lupus erythematosus (SLE) and primary Sjögren’s syndrome (pSS) compared with healthy controls.

Arrows indicate the direction of relative abundance change in patients (↑ increase, ↓ decrease). Grey shading highlights taxa exhibiting opposing abundance patterns between oral and gut microbiomes consistent with a potential oral–gut microbial shift. Bold font indicates microbial alterations observed in both diseases. Only relative abundance data were included. Created in Microsoft Word.

Data on species level were often limited. Nevertheless, additional taxa were identified that showed opposing oral and gut relative abundance patterns even though they did not fully meet the predefined inclusion criteria for hypothesis-confirming taxa. Moreover, several microbial alterations overlapped between pSS and SLE, indicating shared patterns across connective tissue diseases.

One of the most consistent observations was a parallel increase of Veillonellaceae and *Veillonella* in both oral and gut microbiome of patients with both diseases (**Table 1**). *Streptococcus* exhibited opposing relative abundance patterns between the oral cavity and the gut in both diseases with a similar trend for *Enterococcus* spp. in pSS (**Table 1**). In contrast, Bacillota (Firmicutes) displayed opposing relative abundance alterations, which were more pronounced in pSS than SLE.

When examining individual anatomical locations separately, additional microbial changes became evident. Shared alterations across pSS and SLE included *Granulicatella*, and *Haemophilus* (both oral decreases), *Megasphera* (faecal increase), Lachnospiraceae, Oscillospiraceae, Faecalibacterium as well as *Faecalibacterium prausnitzii* (all faecal decreases; **Table 2**, **Supplementary Data Sheet 2**). Especially the decrease of *Faecalibacterium prausnitzii* in the gut was consistently reported for both diseases. In addition, decreased oral abundance of *Granulicatella* and *Haemophilus* together with increased intestinal abundance of *Megasphera* was observed in both connective tissue diseases. In contrast to the pSS pattern, a decreased relative abundance of Pseudomonadota in the gut was found in SLE patients.

### 3.6 Effects of medications

**Figure 10:**
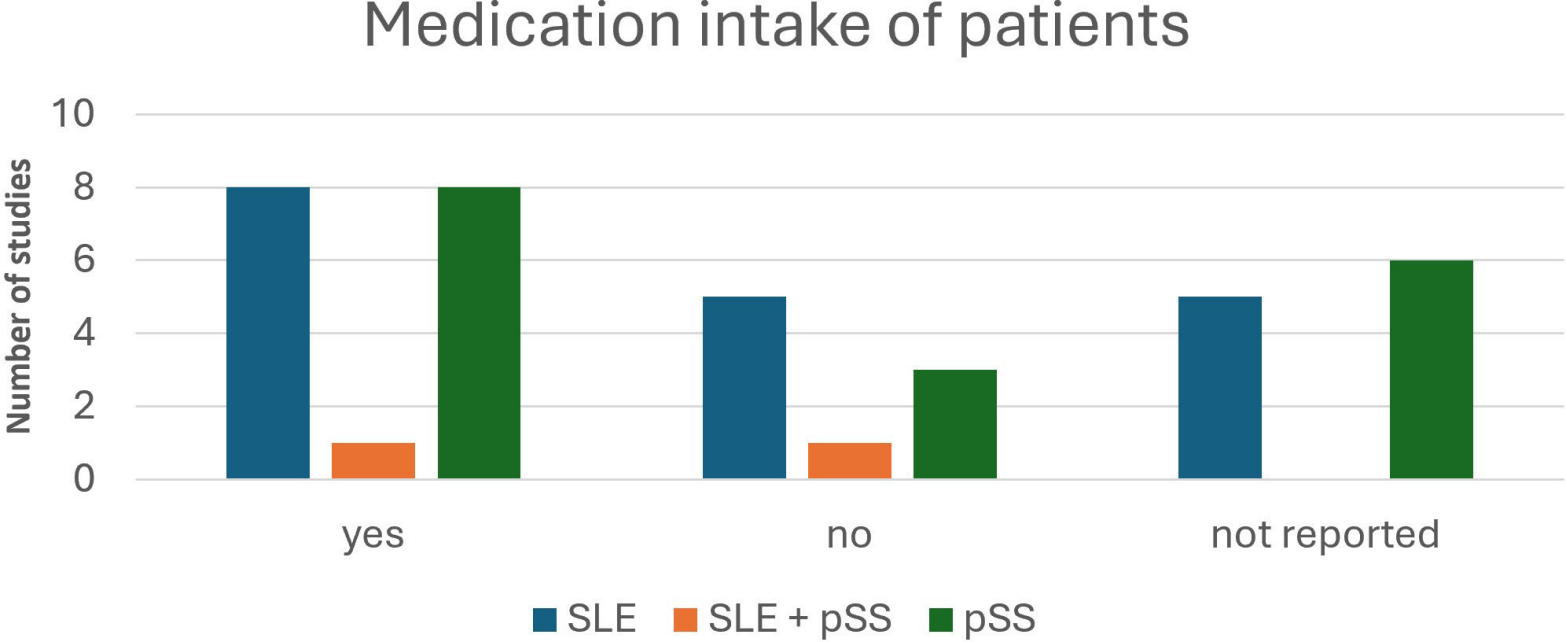
Reported medication use in included patient cohorts. Distribution of studies according to whether medication intake was reported for patients with systemic lupus erythematosus (SLE), SLE with primary Sjögren’s syndrome (SLE + pSS), and primary Sjögren’s syndrome (pSS). Bars indicate the number of studies reporting medication use (“yes”), no medication use (“no”), or not providing information on medication status (“not reported”). Created in Microsoft Word.

Among the included studies, nine investigated treatment-naive patients, while seventeen included patients receiving medication at the time of sampling (**Figure 10**).

Most frequently reported medication were antimalarials including hydroxychloroquine, prednisone, non-steroidal anti-inflammatory drugs (NSAID), proton pump inhibitors (PPI), methotrexate (MTX) and mycophenolate mofetil (MMF).

Further analyses focused on the influence of PPI and cortico steroids regarding microbial alterations across the included studies given its effects on mucosal barriers. In contrast, NSAID use was not assessed because available data was too inconsistently reported from the onset of each study to allow for systematic evaluation.

Data regarding the impact of PPI intake on the microbiome were evaluated, yet insufficient, to allow firm conclusions (47, 54, 61–63). Regarding SLE, only Van der Meulen et al. reported specific data on PPI use, with 18 of 28 patients receiving PPI treatment (63). For pSS, five studies provided data on PPI use (3 oral, 2 gut) (47, 54, 61–63). A summary table with available data on the impact of PPI on the microbiome is included in the **Supplementary Data Sheet 3**.

**Figure 11:**
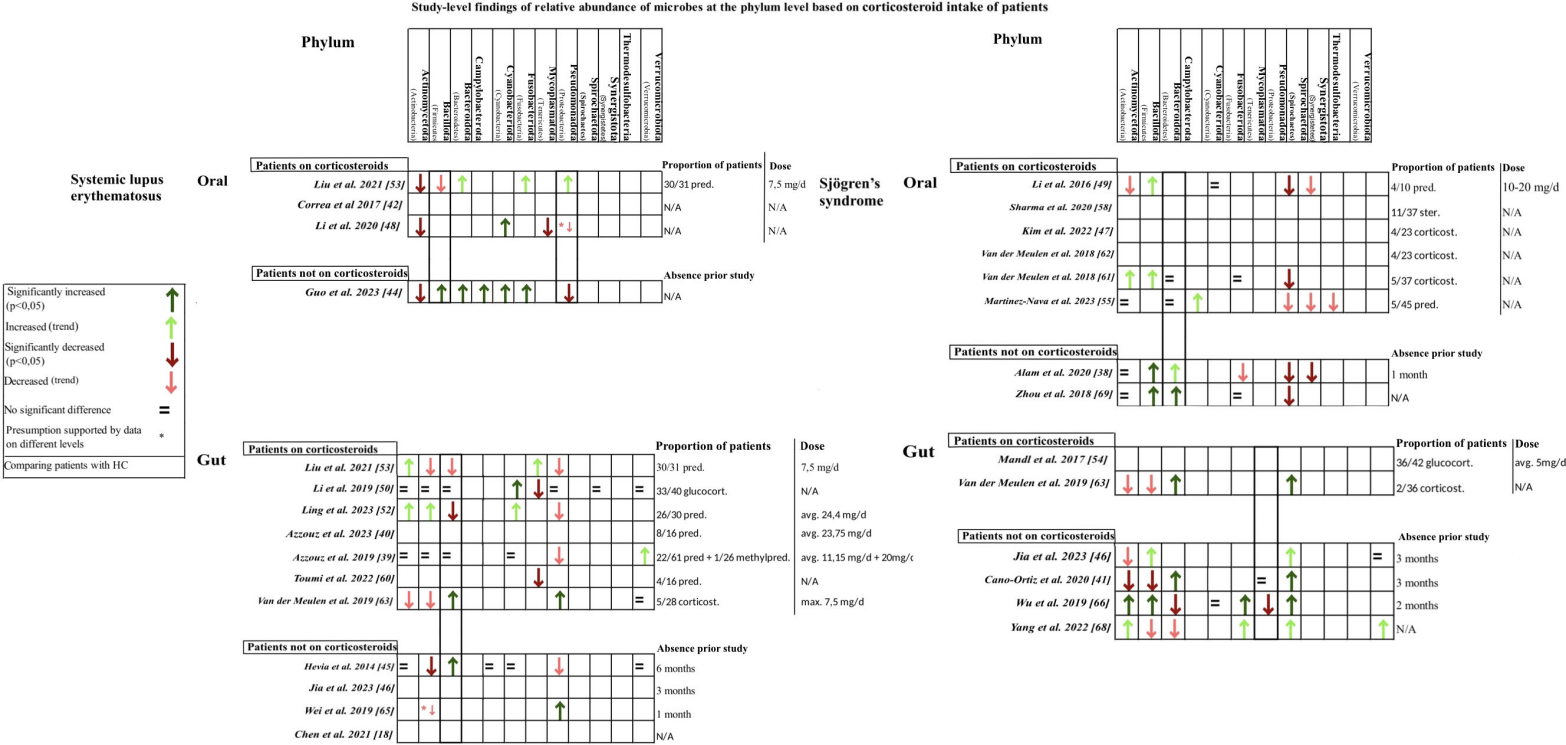
Microbial alterations associated with corticosteroid use, dosage, and patient numbers. Schematic overview of relative microbial abundance changes on the phylum level associated with corticosteroid use in patients with systemic lupus erythematosus (SLE) and primary Sjögren’s syndrome (pSS). Oral and gut microbiome alterations are displayed separately. For each taxon, information on corticosteroid use, reported dosage, and the number of patients included in the respective studies is indicated where available. Arrows represent the direction of relative abundance change in patients compared to HC, green reflecting increase (dark = significantly, light = trend), red decrease (dark = significantly, light = trend) and black equal signs reflects no significant difference. Stars (*) denote presumptions based on underlying taxonomic levels, supported by at least two independent significant study results. Only relative abundance data was included. Bold colums represent main findings on phylum level. Created with Microsoft Excel.

Data on corticosteroid treatment were more extensive (18, 38–42, 44–50, 52–55, 58, 60–63, 65, 66, 68, 69) allowing the possibility of drawing conclusions from the available data (**Table 3**, **Figure 11**). A summary table of corticosteroid treatment is also available in the **Supplementary Data Sheet 4**.

**Table 3:** Relative microbial abundance changes associated with steroid treatment.

Disease	Taxonomy	Oral↑	Oral↓	Gut↑	Gut↓
SLE	phylum	Pseudomonadota	Bacillota		Bacteroidota
	family				
	genus		*Sphingomonas*	*Ruminococcus*	
	species			*Phocaeicola plebius*	
pSS	phylum		Bacteroidota		
	family				
	genus	*Lactobacillus*			*Alloscardovia*
	species				

Summary of bacterial taxa showing relative abundance differences between corticosteroid-treated and non-treated patients with systemic lupus erythematosus (SLE) and primary Sjögren’s syndrome (pSS). Oral and gut microbiome data are presented separately.

Arrows indicate the direction of relative abundance change in corticosteroid-treated patients compared with untreated patients (↑ increase, ↓ decrease). Reported changes are based on relative comparisons within individual studies and do not imply absolute abundance differences. Only taxa for which corticosteroid-associated comparisons were available are shown. Created in Microsoft Word.

**Figure 12:**
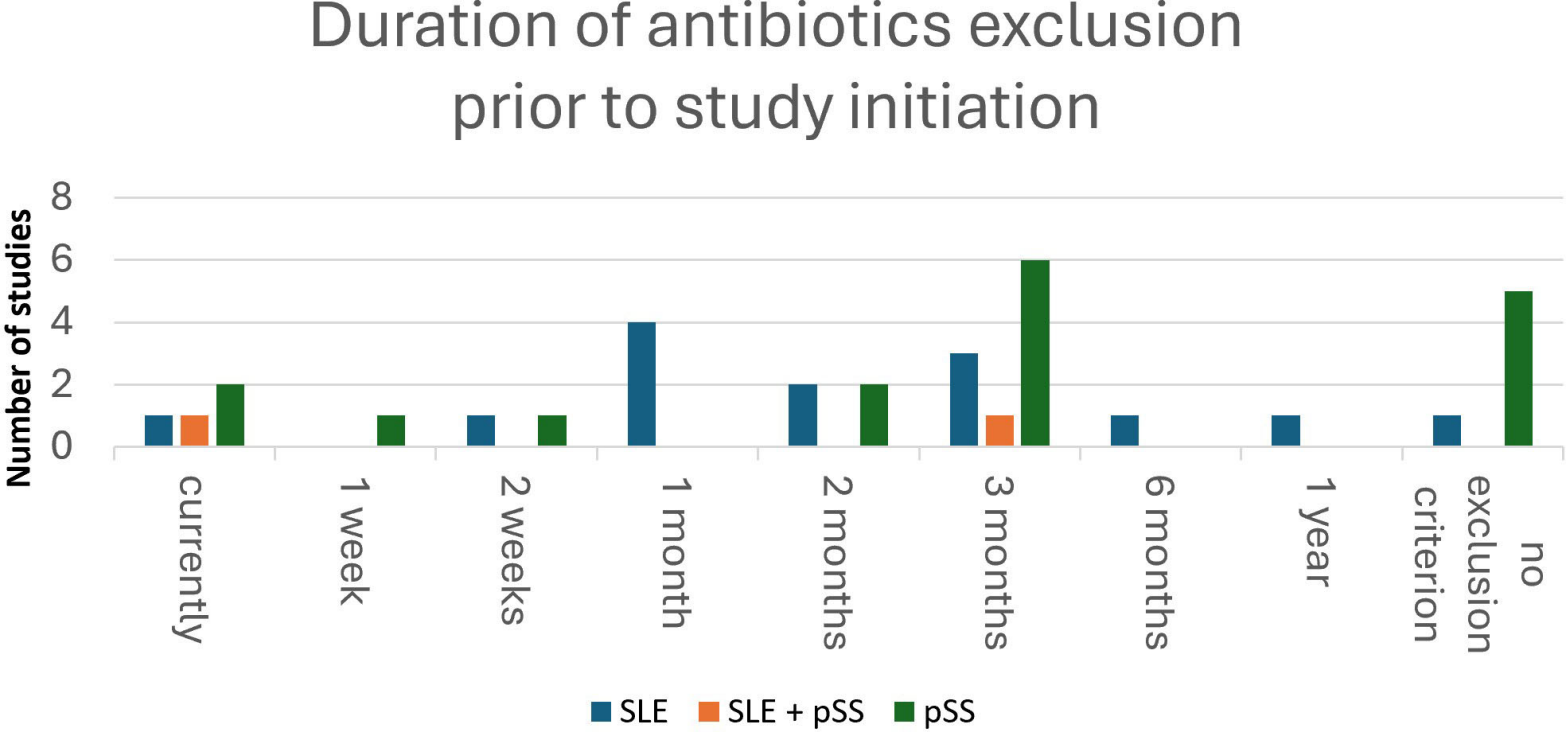
Antibiotic-related exclusion criteria in included studies. Distribution of studies according to the minimum antibiotic-free period required for participant inclusion in cohorts with systemic lupus erythematosus (SLE), SLE with primary Sjögren’s syndrome (SLE + pSS), and primary Sjögren’s syndrome (pSS). Bars indicate the number of studies excluding participants based on recent antibiotic use at different time intervals prior to sampling. Studies with no exclusion of antibiotic use are indicated as “no exclusion criterion”. Created in Microsoft Word.

Comparisons between corticosteroid-treated and non-treated patients were based on relative abundance data within individual studies. An observed increase in one group did not necessarily indicate an absolute increase but may also reflect a less pronounced decrease. However, data availability remained limited, particularly for the oral microbiome. A potential confounder microbiome studies is antibiotic use (71), which can substantially influence microbial composition. In most of the included studies, antibiotic use was an exclusion criterion (n = 27), as detailed in **Figure 12**. However, the required antibiotic-free intervals varied across studies, which may still have affected the observed microbial alterations.

In SLE patients, corticosteroid intake was associated with increased oral Pseudomonadota and decreased Bacillota and *Sphingomonas* abundance. In the gut microbiome, corticosteroid use was associated with increased *Ruminococcus* and *Phocaeicola plebius* and decreased Bacteroidota abundance. In pSS, corticosteroid-treated patients showed increased oral *Lactobacillus* and decreased Bacteroidota abundance as well as reduced *Alloscardovia* in the gut.

Additional findings regarding medication-associated microbial alterations were reported in individual studies. Prednisone dosage correlated with periodontal disease and increased abundance of *Fretibacterium* and *Porphyromonas gingivalis* (42). Corticosteroid treatment was reported to alter *Lactobacillus* and *Streptococcus* abundance (49). Conversely, patients without prior glucocorticoid use exhibited an increased relative abundance of Actinobacteria (phylum), Veillonellaceae, Actinomycetaceae and Micrococcaceae (families) as well as *Veillonella, Rothia, Actinomyces* (genus) together with a significant decrease in *Prevotella and Olsenella* (48). Nevertheless, some studies reported no detectable association between corticosteroid intake and microbial alterations (40, 50, 54).

## 4 Discussion

### 4.1 Main findings of the systematic review

This review synthesizes current evidence on alterations of the oral and gut microbiomes in connective tissue diseases. Our findings indicate that SLE and pSS are associated with coordinated altered microbial profiles across both anatomical sites. Across studies, patients showed a frequent reduction in α-diversity Shannon and Simpson index in the intestine and an increase in the oral cavity. This pattern can be put in the context of potential oral–gut microbial interactions. Importantly, our findings identified recurring patterns of microbial relative abundance alterations relevant to the hypothesis of an oral-gut microbiome axis with oral-to-gut relocation of specific taxa. Across multiple independent studies, *Streptococcus* and Actinomycetota in SLE as well as Pseudomonadota in pSS were reported to be decreased in the oral cavity and increased in the gut microbiome. Both diseases shared opposing oral and gut relative abundance patterns for *Streptococcus*, although the strength and consistency of this pattern varied across studies. *Enterococcus* showed a similar trend in pSS but not SLE although certain *Enterococcus* spp. are highly implicated in the pathogenesis of SLE and could promote type-I interferon and pathogenic T and B cell responses in autoimmunity more generally (72–74). Their detection in stool studies is, however, limited due to their adhesion and translocation in the upper gastrointestinal tract (72–74). Moreover, several microbial changes were found with many also shared between both diseases such as the increase of Veillonellaceae and *Veillonella* in the oral and gut microbiome. This observation is compatible with the possibility of shared pathogenic pathways, potentially involving oral–gut microbial interactions. Together, these findings highlight the potential relevance of the oral–gut axis in connective tissue diseases and support the consideration of multi-site microbial alterations in future investigations of disease mechanisms and therapeutic strategies.

### 4.2 Integration with the existing literature

To contextualize our findings within the rapidly evolving field, an updated literature search was conducted up to 3^rd^ December 2025, focusing on recent systematic reviews and meta-analyses addressing the microbiome in autoimmune and connective tissue diseases. The search resulted in seven additional findings. These were mainly related to external treatment interventions (75–78), the microbiome in systemic scleroderma (79), the role of the gut microbiome in ankylosing spondylitis and arthritis (80) and the oral microbiome in pSS (81). Notably, only four newly published primary studies investigated the oral microbiome, and all were restricted to pSS, underscoring the persistent lack of oral microbiome data in SLE and other connective tissue diseases.

The updated database search yielded the following results:

[Systemic Lupus Erythematosus] MeSH AND [Microbiota] MeSH → 200 results (+33)

[Systemic Lupus Erythematosus] MeSH AND [Microbiota] MeSH AND [Mouth] MeSH → 9 results (unchanged)

[Sjögren´s syndrome] MeSH AND [Microbiota] MeSH → 72 results (+13)

[Sjögren´s syndrome] MeSH AND [Microbiota] MeSH AND [Mouth] MeSH → 15 results (+4)

[Scleroderma, Systemic] MeSH AND [Microbiota] MeSH → 51 results (+ 10)

[Scleroderma, Systemic] MeSH AND [Microbiota] MeSH AND [MOUTH] MeSH → 1 result (unchanged)

[Myositis] MeSH AND [Microbiota] MeSH → 9 results (unchanged)

[Myositis] MeSH AND [Microbiota] MeSH AND [MOUTH] MeSH → 1 result (unchanged)

The newly identified studies reported an increase of Pseudomonadota (Proteobacteria) in the gut of SLE patients (82), a pattern only partially observed in the prior SLE studies but more clearly in the prior pSS cohorts, also confirmed by newer studies (83). Importantly, intestinal enrichment of Pseudomonadota (Proteobacteria) has also been described as a feature of lupus nephritis (84), suggesting that this microbial signature may be linked to disease severity and organ involvement. Additional evidence of overlapping microbial traits between SLE and inflammatory bowel disease support this notion of cross-disease microbial patterns (11).

Enrichment of *Streptococcus* in the gut has been associated with impaired intestinal barrier function and inflammation (85), supporting its potential role in systemic immune activation. Such barrier disruption and translocation of gut pathobionts contributes to further organ damage (72–74, 86, 87). Together, these findings support the hypothesis that orally associated *Streptococcus* taxa may contribute to intestinal immune dysregulation when enriched in the gut, thereby potentially influencing disease severity and progression. Similarly, *Veillonella* enrichment has emerged as a recurring feature in both the oral microbiome (88) and the gut microbiome (83) of pSS patients, reinforcing its role as a shared marker across both anatomical sides.

Despite the expanding literature on microbiome alterations in connective tissue diseases, studies explicitly interrogating an oral–gut microbiome axis, especially with designs that allow mechanistic or therapeutic inferences, remain scarce. This gap underscores the relevance of the present study. By integrating evidence across oral and gut compartments in both SLE and pSS, this review provides a cross-body site perspective on dysbiosis and identifies recurrent taxa-level patterns that may inform hypotheses on mucosal immune dysregulation and systemic disease features. Moreover, the consistency of opposing patterns across independent cohorts highlights the promise of the oral–gut axis as a future therapeutic target, supporting the development of microbiome-based interventions in these diseases.

### 4.3 Biological interpretation

#### 4.3.1 α and β diversity

β-diversity analyses consistently demonstrated separation between patients and healthy controls across both oral and gut microbiome studies, indicating global differences in microbial community structure (89). In contrast, α-diversity measures showed site-specific and heterogeneous patterns, particularly for Shannon and Simpson indices (**Figure 6**).

Across studies, microbial richness, most frequently assessed by Chao1, was commonly reduced in patients, most consistently in the gut and less prominently in the oral microbiome of both SLE and pSS (39, 41, 48, 50–53, 59, 63–67). Reduced intestinal richness has been linked to impaired epithelial barrier integrity and decreased colonization resistance, potentially facilitating expansion or unmasking of pathobionts (90). Richer communities have previously been associated with greater functional redundancy and metabolic resilience (89) suggesting that the beneficial community structure is compromised in these chronic disease states.

In contrast, oral richness patterns were inconsistent and appeared strongly influenced by local inflammatory conditions, such as periodontal status (42). Liu et al. similarly observed reduced Chao1 and Abundance-based Coverage Estimator (ACE) indices in the gut of SLE patients but found no significant difference in saliva from the same cohort (53). This finding supports site-specific effects rather than global richness loss. Several other oral studies also reported no significant Chao1 difference (58, 61, 69). As three of these studies (53, 58, 69) analysed saliva samples and one used buccal swaps (61), the consistent findings may reflect similarities in oral sampling methods.

More pronounced compartment-specific differences emerged when considering Shannon and Simpson diversity indices. The Simpson index represents the probability that two randomly selected species belong to the same species whereas the Shannon index reflects community entropy (89). Shannon is often regarded as the most informative among standard diversity metrics (91).

Several studies reported reduced diversity in the gut microbiome (17, 18, 40, 41, 46, 53, 60, 63, 64, 66) whereas increased diversity was more frequently observed in oral samples (38, 44, 53, 55, 67). This opposing pattern was also demonstrated within the same SLE patient cohort (53) supporting a site-specific rather than cohort-driven effect. Azzouz et al. further linked gut bacterial community variance to high disease activity (SLEDAI >8) (40) suggesting an association between intestinal microbial instability and systemic inflammation. In addition, Greiling et al. found reduced oral and faecal α-diversity in Ro60-negative SLE patient subsets (17). Nevertheless, a substantial proportion of studies reported no significant α-diversity differences (40, 43, 45, 47, 48, 50–52, 56–58, 61, 65, 68, 69), highlighting considerable inter-study heterogeneity.

Reduced gut α-diversity has been associated with systemic inflammation, impaired barrier function (92), and adverse clinical outcomes (93), whereas increased oral diversity has been linked to inflammatory oral conditions and systemic immune activation (94). Inflammatory oral dysbiosis may promote the expansion and downstream dissemination of orally associated pathobionts, particularly in the context of reduced intestinal colonization resistance (90). For translocation-driven inflammatory effects to occur, two conditions appear critical: (i) inflammatory oral dysbiosis and (ii) impaired gut barrier function, often accompanied by reduced microbial diversity (90).

Together, the combination of reduced intestinal diversity and preserved or increased oral diversity is compatible with the proposed oral–gut axis hypothesis. However, α-diversity metrics alone cannot establish causality, and these observations should only be interpreted as suggestive for coordinated oral–gut microbial alterations in SLE and pSS.

#### 4.3.2 Oral-gut microbial relocation

Building on the observed compartment-specific diversity patterns, taxon-level analyses identified several microbial groups displaying opposing abundance trends between the oral cavity and the gut. These findings are compatible with oral–gut microbial redistributions in connective tissue diseases. These include Actinomycetota and *Streptococcus* in SLE and Pseudomonadota in pSS, fulfilling the predefined criteria for opposing oral-gut abundance patterns. Additional patterns, including concurrent oral decreases and concurrent intestinal increases of oral-associated genera, further support this paradigm.

The concept of oral–gut microbial translocation(or "relocation" given that no physical barriers are in most instances overcome) is increasingly supported by ecological and clinical evidence, suggesting that orally associated taxa can contribute to intestinal immune perturbation under permissive conditions. Periodontal inflammation has been linked to intestinal inflammatory responses with oral pathobionts shown to accumulate in the gut and to induce local immune activation (23). Oral–gut dissemination has been implicated in multiple disease contexts including gastric carcinogenesis (95), colorectal cancer (96), rheumatoid arthritis (24) and both irritable and inflammatory bowel diseases (96). Immune dysregulation and relative suppression, a frequent feature in systemic autoimmune diseases, may facilitate ectopic gut colonization by oral bacteria, especially when accompanied by oral dysbiosis (96).

Although the precise pathways of oral–gut bacterial redistribution remain incompletely defined, three principal routes have been proposed: (i) enteric, (ii) hematogenous, and (iii) immune-mediated dissemination (97). The enteric route involves the survival and passage of oral bacteria through the gastrointestinal tract (98), a process normally constrained by gastric acidity and intestinal colonization resistance (24, 97). Accordingly, successful intestinal establishment of oral pathobionts appears to involve two conditions: (i) disruption of gut microbial and chemical (gastric) barriers and (ii) inflammatory dysbiosis within the oral cavity (23).

Our findings highlight a consistent intestinal decrease of several Bacillota (Firmicutes) families, including Lachnospiraceae and Oscillospiraceae (formerly Ruminococcaceae), particularly the genus *Faecalibacterium* and the key species *Faecalibacterium prausnitzii*. These taxa are reported as major butyrate producers essential for epithelial barrier integrity and immune homeostasis (99). *F. prausnitzii*, the dominant butyrate producer in the human colon (100), is characteristically depleted during chronic inflammatory states (99). Loss of these commensals may weaken colonization resistance and epithelial barrier integrity, thereby creating a permissive niche for orally associated taxa to expand within the gut microenvironment. Nevertheless, oral bacteria can reach the gut even in healthy individuals; approximately one-third of oral microbial cells were shown to transit the gastrointestinal tract (24) with translocation increasing with age (21).

A second, less likely, potential mechanism is the hematogenous spread (95), mainly suggested for Fusobacterium to colonize colon cancers, although a direct oral-gut relocating route is supported by more recent data (101). Several oral taxa have been detected at low abundance in both blood and intestinal samples (102). *Streptococcus*, particularly *S. salivarius*, exhibits consistent presence across oral, blood, and gut compartments (102), supporting its capacity for systemic dissemination beyond its primary oral niche as also was shown for type 1 diabetes (103) and cardiovascular diseases (102). In both SLE and pSS, *Streptococcus* displayed a reduction in the oral cavity, yet enrichment in the intestine, although this pattern was more consistent in SLE. *Streptococcus* is the genus with the highest number of shared ASVs across oral and gut habitats (21), is associated with systemic inflammatory signatures (97) and has been implicated in gastric carcinogenesis (95). These findings highlight its potential immunopathogenic relevance beyond the oral cavity. As a dominant biofilm-forming genus frequently co-occurring with *Actinomyces* (104), *Streptococcus* may benefit from cooperative biofilm dynamics that facilitate survival during translocation and subsequent intestinal engraftment. Importantly, some degree of *Streptococcus* transfer also occurs in healthy individuals (96) suggesting that disease-related dysbiosis modifies the immunological consequences. In addition, *Streptococcus* spp. as well as *Enterococcus* spp. can translocate directly across dysfunctional oral or upper gastrointestinal epithelial barriers to promote systemic autoimmunity related to rheumatoid arthritis (105) and SLE (72–74). In these settings, faecal presence may not be overt in human association studies given that direct epithelial translocation into lymphoid organs and target tissues may occur as was also shown for other pathophysiologic states (106, 107).

Evidence for immune-cell-mediated routes remains scarce, though this pathway is of interest given the impaired or dysregulated immune function characteristic of connective tissue diseases.

A key unresolved question is whether relocated oral bacteria actively expand within the gut (expansion hypothesis) or whether their increased relative abundance reflects loss of competing gut commensals (marker hypothesis) (108). Liao et al. provide strong support for the marker hypothesis, demonstrating that microbiome–disease associations are driven predominantly by depletion of core gut commensals rather than true expansion of disease-associated taxa (108). These findings suggest that apparent oral–gut shifts may require concurrent oral dysbiosis and loss of gut microbial resilience with oral taxa acting as indicators of an altered intestinal ecosystem rather than primary drivers of dysbiosis. In this context, increases in the relative abundance of orally associated taxa do not necessarily imply numerical expansion but may instead reflect reduced abundance of common gut commensals within a destabilized microbial community. This interpretation aligns with the consistent depletion of butyrate-producing taxa such as *Faecalibacterium prausnitzii* observed in both SLE and pSS, which may lower colonization resistance and unmask oral taxa in relative abundance analyses. Importantly, even when acting primarily as markers of dysbiosis, orally associated taxa may exert disproportionate immunological effects as oral pathobionts as they have been shown to preferentially induce Th17 responses upon ectopic gastrointestinal localization (23). Also, strain-resolved and functional studies argue against an exclusively passive marker role of oral taxa. Atarashi et al. demonstrated that ectopically colonizing oral *Klebsiella* strains can expand in the gut and induce Th1 responses (22), while Chen et al. provided evidence for oral-to-gut relocation of taxa including Ro60 (autoantigen) ortholog-expressing *Actinomyces massiliensis* in treatment-naïve SLE patients (17, 18). These findings suggest that, under conditions of reduced colonization resistance, translocated oral bacteria may not only mark dysbiosis but also actively contribute to immune modulation, indicating that the marker and expansion hypotheses are not mutually exclusive. The oral-to-gut relocation of Ro60-positive bacteria, specifically *A. massiliensis*, originally profiled by us longitudinally in SLE patients (17) but not at strain-level resolution, prompted this systematic review of other SLE studies with oral and/or gut microbiome data.

In pSS, the phylum Pseudomonadota (Proteobacteria) displayed the most consistent opposing oral–gut abundance pattern. Although mechanistic insights at the phylum-level remain limited, Pseudomonadota is one of the dominant oral phyla (109), making them plausible contributors to oral–gut microbial redistribution. Enterobacteriaceae, a major family within Pseudomonadota, are frequently enriched during oral dysbiosis and capable of inducing intestinal inflammation in immunocompromised hosts (23). Blooms of *Klebsiella* and *Enterobacter* are also found to correlate with exacerbation of intestinal inflammation (23). *Klebsiella* is a well-characterized oral pathobiont capable of gut colonization (21) and a potent inducer of Th1-mediated inflammation (22). Similarly, Actinomycetota, particularly the genus *Actinomyces*, is abundant in the oral cavity (109) and has been documented to translocate to the gut (24) including in SLE patients when the oral origin of faecal *A. massiliensis* was tracked (18), a Ro60 ortholog-carrying pathobiont (17). Nevertheless, evidence supporting active intestinal expansion of Actinomycetota remains limited on the phylum level and observed gut enrichment may primarily reflect reduced competition from obligate anaerobic commensals rather than true engraftment.

These phylum-level signatures in SLE and pSS are rarely reported and warrant further investigation. Future studies should determine whether pathogenicity arises from specific taxa or whether broader phylum-level traits contribute to oral–gut shifts. Additionally, it remains unclear whether oral bacterial abundance truly declines during translocation to the gut or whether oral taxa continue to proliferate locally while simultaneously seeding the gut. This uncertainty represents a limitation of our approach although the relative abundance data remain informative for complementary analyses such as *in-vivo* mechanistic work as done for other disease states (22).

Finally, both diseases, SLE and pSS, exhibited consistent oral and intestinal increases in Veillonellaceae, particularly *Veillonella*. *Veillonella* abundance has been associated with elevated inflammatory cytokine levels (98), and *V. parvula* possesses metabolic flexibility allowing growth on organic acids and enhanced immune evasion, facilitating gut colonization (97). Veillonellaceae expansion is also observed in immunocompromised individuals (96). Additional corresponding changes include oral decreases in *Granulicatella* and *Haemophilus* and intestinal increases in *Megasphera*. *Haemophilus* depletion has been reported in rheumatoid arthritis and associated with systemic inflammation (110) and autoantibody levels (111).

Together, the collective interpretation of diversity metrics, taxon-specific patterns, and immunological contexts suggests that oral–gut microbial alterations in connective tissue diseases arise from an interplay of compartment-specific dysbiosis, impaired mucosal barrier function, and disease-specific immune dysregulation besides oral-to-gut translocation mechanisms.

#### 4.3.3 Impact of medications on oral and gut microbial communities

We examined whether medication use influences the microbiome beyond disease-related effects and to what extent such influences may shape the observed oral and gut microbial patterns. It is well established that gut microbial signatures are shaped not only by disease itself but also by therapeutic variables (82), complicating causal interpretations. Given that PPI have the most profound effect on the gut microbiome (112) and corticosteroids are routinely used in CTDs, we were particularly interested in studying those treatment effects on oral and gut microbiomes.

PPI therapy has been shown to substantially alter the gut microbiome and associated metabolic pathways (113). This is of particular relevance to our research question as PPI use has been associated with the development of autoimmune diseases (71) and may facilitate oral–gut microbial shifts by reducing gastric acidity, thereby increasing survival of orally derived bacteria during gastrointestinal transit (114). In addition, PPIs have been reported to disrupt the intestinal epithelial barrier by interfering with reactive oxygen species, thereby inducing structural damage to the intestinal mucosa (71). Such barrier impairment could further promote oral–gut microbial shifts, especially in an already dysbiotic intestinal ecosystem.

In PPI-treated SLE patients, increased abundances of *Lactobacillus*, *Roseburia*, *Oxalobacter*, and *Desulfovibrio* have been reported together with decreased levels of *Veillonella*, *Escherichia*, *Morganella*, and *Pseudomonas*, suggesting partial shift toward a microbiome composition closer to a healthier state in some contexts (115). Notably, *Streptococcus* abundance appears to be particularly influenced by PPI use. Oral *Streptococcus* species, especially *Streptococcus anginosus*, have been detected more frequently in the gut of PPI users (116), and *Streptococcus salivarius* has also been reported to be increased in the intestines of PPI-treated individuals (113). In our analysis, *Streptococcus* emerged as a potential taxon involved in oral–gut relocation, a phenomenon that may be amplified by PPI use. However, our data are insufficient to draw definitive conclusions regarding PPI-driven oral–gut translocation, underscoring the exploratory nature of these observations.

We also examined the influence of corticosteroid intake on the microbiome. Evidence regarding glucocorticoid-induced microbiome alterations remains heterogeneous, with some studies reporting no association (40, 50, 54) and others identifying significant changes (42, 44, 48, 49). Overall, there is considerably less evidence regarding corticosteroid-induced microbiome alterations in autoimmune diseases compared to PPIs. One study reported that the gut microbiome of glucocorticoid-treated SLE patients more closely resembled that of healthy controls than that of untreated SLE patients (117).

Nevertheless, glucocorticoids have been shown to impair intestinal barrier function by increasing permeability and reducing IgA production (118), which may facilitate microbial redistribution. In our analysis, we identified some microbiome alterations that differed between cortico steroid users and non-users. An increased oral abundance of Pseudomonadota in SLE patients is noteworthy, as this phylum emerged as a potential translocator in pSS. Enhanced glucocorticoid exposure in SLE patients might therefore exert similar effects. Supporting this hypothesis, an increase of Pseudomonadota (Proteobacteria) in the gut of SLE patients has been reported (117). In addition, increased abundances of *Actinobacteria, Bifidobacterium*, and *Lactobacillus* have been observed in glucocorticoid users (119), whereas *Streptococcus* abundance was reported to be decreased in the gut of glucocorticoid-treated SLE patients (117). Moreover, prednisone use has been associated with periodontal destruction in SLE (42), which may secondarily exacerbate oral dysbiosis.

In summary, we did observe differences between the microbiomes of glucocorticoid users and non-users in both SLE and pSS. However, reported microbiome effects of glucocorticoids remain heterogeneous across studies, likely reflecting differences in dosage, treatment duration, and underlying disease activity. Consequently, further research is needed to clarify these associations.

Reporting of other medication classes was inconsistent, making systematic comparisons across studies difficult. This applied particularly to biologic therapies, which were only reported in a small number of cohorts, including isolated reports of belimumab (39) and rituximab (63) use, as well as biologic treatment without further classification (50). Consequently, the potential influence of several treatment modalities on oral and gut microbiome composition could not be adequately evaluated.

Overall, medication use appears to impact the microbiome, but the extent of this effect requires further systematic investigation, especially concerning *Streptococcus* and Pseudomonadota as potential mediators of oral–gut microbial shifts.

### 4.4 Methodological considerations and limitations

Several methodological limitations should be considered when interpreting the findings of this review.

First, data availability across diseases and anatomical sites was uneven. Although multiple connective tissue diseases were screened, the analysis had to focus on SLE and pSS due to a lack of oral microbiome data in other conditions. Even within these diseases, sampling was imbalanced: SLE studies predominantly investigated the gut microbiome, whereas oral data were scarce, while pSS studies showed a more balanced study of both oral and gut microbiomes.

Second, heterogeneity in study populations and control selection likely influenced relative abundance estimates. Healthy controls were not consistently matched for age and sex, and the proportion of male participants was substantially higher among controls than patients. SLE patients were on average younger than controls, and demographic information was incomplete in several studies. In addition, most cohorts were recruited from Asian populations, which may limit generalizability of the findings to other ethnic or geographic groups. Geographical location was also discussed to influence the gut microbiota through diet (65). Dietary influences were inconsistently assessed across studies. While some cohorts attempted to control for dietary habits (44, 45, 48, 59, 65), standardized nutritional assessments were largely lacking, limiting the evaluation of diet-related effects on oral or gut microbial community structures.

Third, substantial methodological heterogeneity was present across studies. Although most investigations used 16S rRNA gene sequencing, targeted regions, sequencing platforms, and bioinformatic pipelines varied. Both OTU- and ASV-based approaches were applied, with ASV inference having been more common in SLE studies. Taxonomic harmonization was therefore necessary to enable cross-study comparisons, but this process may have influenced observed patterns and masked finer-scale differences.

Fourth, oral microbiome sampling was highly heterogeneous, including saliva, supragingival, and subgingival plaque, each representing distinct ecological niches. This limits direct comparability of oral findings across studies and may partially account for inconsistent diversity and abundance patterns.

Fifth, analyses of medication effects were constrained by incomplete reporting. Data on PPI use were extremely limited, and although corticosteroid use was more frequently reported, most studies lacked individual-level information on treatment status, dosage, and duration. Oral microbiome data from corticosteroid-naive patients were particularly scarce, limiting causal inference regarding medication-associated microbial shifts.

Finally, the oral–gut re- or translocation hypothesis was primarily supported by relative abundance patterns and diversity indices. These measures provide indirect evidence and cannot establish causality or confirm active microbial migration. Moreover, several taxa showed trends opposite to the proposed hypothesis, underscoring the complexity of oral–gut microbial interactions.

Despite these limitations, this study has several important strengths. The structured data extraction strategy, predefined criteria for hypothesis-confirming taxa, and formal quality assessment of included studies reduced the risk of selective interpretation. Taxonomic harmonization using updated classifications enabled meaningful comparison across studies published in over a decade.

Notably, consistent opposing abundance patterns between oral and gut compartments were observed across independent cohorts, supporting the biological plausibility of an oral–gut microbial shift. Together, these strengths highlight the relevance of a multi-site microbiome perspective and support further investigation of the oral–gut axis as a contributor to connective tissue disease pathogenesis.

### 4.5 Clinical and research implications

Clinically, the observed patterns support the concept that oral and gut microbiomes should not be considered independent compartments in connective tissue diseases. The consistent coexistence of oral dysbiosis and intestinal microbial instability suggests that maintaining gut microbiome resilience may be critical for preventing the expansion or persistence of orally derived pathobionts in the intestine such as *A. massiliensis* (17, 18). Strategies aimed at preserving gastric (chemical) barrier integrity and microbial diversity may therefore represent a protective mechanism against oral–gut microbial shifts.

In this context, microbiome-modulating interventions warrant further investigation. While probiotics and dietary interventions are being explored in SLE and pSS, more comprehensive approaches such as faecal microbiota transplantation (FMT) should be examined systematically. FMT has the theoretical potential to restore a stable, diverse gut microbial ecosystem, which may enhance colonization resistance and reduce susceptibility to oral bacterial engraftment (108). However, its safety, durability, and disease-specific efficacy in connective tissue diseases remain largely unexplored and require carefully designed clinical trials beyond pilot studies (34). More promising are tailored microbiome targeting approaches such as bacteriophage therapy against defined pathobionts as explored in other inflammatory conditions (120).

From a research perspective, future studies should adopt longitudinal and multi-site sampling designs, simultaneously assessing oral and gut microbiomes within the same individuals. Integration of absolute abundance data, strain-level resolution, and functional readouts such as metabolomics or host immune markers will be essential to distinguish true microbial re- or translocation from secondary or relative effects. In addition, standardized reporting of medication exposure, antibiotic history, oral health status, and sampling protocols is necessary to improve comparability across studies. Finally, mechanistic studies in *in-vivo* models (22) are warranted to dissect further cause and effect of the oral and gut microbial community structures.

## 5 Conclusions

This systematic review demonstrates that SLE and pSS are associated with coordinated dysbiosis of the oral and gut microbiomes. Consistent opposing abundance patterns across anatomical sites, most notably involving *Streptococcus* and Actinomycetota in SLE and Pseudomonadota in pSS, support the hypothesis of an oral–gut microbial shift contributing to disease-associated immune dysregulation.

Although current evidence does not establish causality, the convergence of diversity metrics, taxonomic patterns, and biological plausibility highlights the oral–gut axis as a relevant and previously underexplored dimension of connective tissue disease pathogenesis. Future longitudinal, mechanistic, and interventional studies are needed to determine whether stabilizing the gut microbiome can ameliorate oral–gut microbial translocation and influence clinical outcomes. Addressing this axis may open new avenues for microbiome-based prevention and therapeutic strategies in connective tissue diseases.

## Data Availability

The original contributions presented in the study are included in the article/**Supplementary Material**, further inquiries can be directed to the corresponding author/s.

## Supplementary material

Supplementary Figure 1 Matching variables across study groups.

Supplementary Figure 2 Proportion of female participants across study groups.

Supplementary Figure 3 Mean age of patients and healthy controls across study groups.

Supplementary Figure 4 Taxonomic levels at which differential abundance data were reported.

Supplementary Figure 5 16S rRNA gene variable regions targeted for sequencing.

Supplementary Figure 6 Taxonomic profiling approaches used in included studies.

Supplementary Table 1 Geographic distribution of included studies.

Supplementary Table 2 Overview of data requests and author responses for included studies.

Supplementary Table 3 Methodological quality assessment of included studies.

Supplementary Table 4 Updated bacterial phylum nomenclature used in this study.

Supplementary Table 5 Updated bacterial family nomenclature used in this study.

Supplementary Table 6 Updated bacterial genus nomenclature used in this study.

Supplementary Table 7 Updated bacterial species nomenclature used in this study.

Supplementary Data Sheet 1 Data extraction table.

Supplementary Data Sheet 2 Relative abundance changes in oral and gut.

Supplementary Data Sheet 3 Relative abundance changes associated with PPI use.

Supplementary Data Sheet 4 Relative abundance changes associated with corticosteroid use.
