# Supplementary material for: Coordinated oral–gut microbiota relocation in connective tissue diseases: a systematic review
Source: Front Immunol. 2026 Jul 3;17:1841874. doi: 10.3389/fimmu.2026.1841874 (PMC13376073; doi:10.3389/fimmu.2026.1841874)
Supplement: Supplementary Figure 1 — Matching variables across study groups. [file SupplementaryFile1.docx]

Supplementary Material

# Supplementary Results

# S1: Study characteristics and cohort composition

This section provides an extended descriptive overview of study characteristics and cohort composition across the included studies. These data are presented to document the heterogeneity of study designs and populations but were not considered central to the primary outcome analyses.

## 1.1 Matching variables

Supplementary Figure 1: Matching variables across study groups.

The bar chart shows the distribution of matching variables used in the included studies for systemic lupus erythematosus (SLE), SLE with primary Sjögren’s syndrome (SLE + pSS), and primary Sjögren’s syndrome (pSS). Variables include age, sex, combined age and sex, age and body mass index (BMI), age, sex and BMI, lifestyle-related factors (smoking status, dietary habits, and physical activity, oral health–related variables) no matching, and no information provided. Numbers indicate the count of studies applying each matching strategy per group. Created in Microsoft Word.

Additional information:

*Jia et al.* [46]: Healthy controls (HC) and pSS groups were matched for age, sex, and BMI. pSS and SLE groups were matched for age and BMI.

*Azzouz et al.* [40] (SLE): No information provided regarding matching.

## Sex distribution

Supplementary Figure 2: Proportion of female participants across study groups.

The bar chart illustrates the distribution of studies according to the reported proportion of female participants in systemic lupus erythematosus (SLE), SLE with primary Sjögren’s syndrome (SLE + pSS), and primary Sjögren’s syndrome (pSS). Categories represent percentage ranges of female participants per study. Bars indicate the number of studies within each category; studies with no reported sex distribution are shown as “no information”. Created in Microsoft Word.

Additional information:

Majority of patients are female (female predominance in SLE and pSS) 🡪 Female-to-male ratio (F:M) 10.1 :1

HC groups contain relatively more males 🡪 (F:M) 2.9:1

**SLE:**

Missing data: *Li et al.* [48] F/M ratio for SLE patients, *Toumi et al.* [60] F/M ratio for HC, *Jia et al.* [46] F/M ratio for SLE (although included in pSS).

Ratios:

- 6 studies with **100% female** patients
- Remaining studies: 2x 94%, 93%, 91%, 88%, 79%, and 48% female.

**Overall ratio SLE**:

- 455 females / 49 males → **9.3: 1** (F:M).
- Including partial data from *Guo et al.* [44] (where only part of the cohort is specified):

586 females / 58 males → **10.1: 1** (F:M).

Patients with unspecified gender (*Guo et al*. [44], *Jia et al*. [46], *Li et al*. [48]): n = 184.

**pSS:**

Ratios:

- 8 studies with **100% female** participants.
- Remaining studies: 97%, 95%, 94%, 93%, 86%, 84%, 83%, 69%, 30% female.

**Overall ratio pSS:**

507 females / 50 males → **10.1: 1** (F:M).

**HC:**

Ratios:

- Total: 1,515 females / 520 males → **2.9 : 1** (F:M).
- Most HC data derived from *Van der Meulen et al*. [63] (557 F / 405 M).
- Missing data: *Toumi et al.* [60] (n = 77) and *Li et al.* [48] (n = 19): F/M ratio not available; *Kim et al.* [47] included only non-SS sicca comparators (no HC) – no data.

## Age distribution

Only studies with adult patients were analysed.

Supplementary Figure 3: Mean age of patients and healthy controls across study groups.

The bar chart shows the mean age of patients and healthy controls (HC) in studies with systemic lupus erythematosus (SLE) and primary Sjögren’s syndrome (pSS). Bars represent the average reported age across studies for each group. The standard error of the mean (SEM) is represented by the error bars. Created in Microsoft Word.

**Mean ages** (pooled averages across studies):

- SLE patients: 39.61 years
- pSS patients: 53.16 years
- HC (for SLE studies): 37.84 years
- HC (for pSS studies): 46.88 years

**SLE**

Mean age range: 30.8 years (*Chen et al*. [18] ) – 49.2 years (*Hevia et al.* [45]).

Missing age data:

*Jia et al.* [46] included age-matched pSS and HC, but no data on the age of SLE age.

**pSS**

Mean age range: 38.4 years (*Guo et al.* [44]) – 62 years (*Mandl et al*. [54] ).

van der Meulen et al*.* [63] included both patient groups (SLE and pSS) and their HC cohorts, counted twice for HC calculations.

**HC**

Missing age data:

*Azzouz et al*. [39], *Toumi et al*. [60], *Azzouz et al*. [40], *Jia et al.* [46] (SLE), *Sharma et al*. [58]

## Geographic origin

Supplementary Table 1: Geographic distribution of included studies.

|  |  | **SLE** | **SLE + pSS** | **pSS** | tot |  |
| --- | --- | --- | --- | --- | --- | --- |
| **Asia** | China | 9 | 1 | 6 | 16 | **20** |
|  | India |  |  | 1 | 1 |  |
|  | South Korea |  |  | 3 | 3 |  |
| **Europe** | Spain | 1 |  | 1 | 2 | **5** |
|  | Netherlands |  | 1 |  | 1 |  |
|  | France | 1 |  |  | 1 |  |
|  | Sweden |  |  | 1 | 1 |  |
| **America** | USA | 2 |  | 2 | 4 | **6** |
|  | Mexico |  |  | 1 | 1 |  |
|  | Brazil | 1 |  |  | 1 |  |
| **Undetermined** | N/A |  |  | 2 | 2 | **2** |

Distribution of studies by geographic region and country for systemic lupus erythematosus (SLE), SLE with primary Sjögren’s syndrome (SLE + pSS), and primary Sjögren’s syndrome (pSS). Numbers indicate the count of studies conducted in each country. “N/A” denotes not assigned (studies for which the country of origin was not reported). Since both studies are by a research group from the Netherlands (van der Meulen et al. [61][62]), the patient cohorts likely also originated from the Netherlands. Created in Microsoft Word.

## **Abundance metrics**

Supplementary Figure 4: Taxonomic levels at which differential abundance data were reported.

Distribution of studies reporting microbial abundance data at the phylum, family, genus, and species level in cohorts with systemic lupus erythematosus (SLE), SLE with primary Sjögren’s syndrome (SLE + pSS), and primary Sjögren’s syndrome (pSS). Bars indicate the number of studies reporting differential abundance results at each taxonomic level. Original data from publications as well as derived presumptions based on reported values, were included. Created in Microsoft Word.

Excluded data:

*Kim et al*. [47]: Data excluded; comparison limited to pSS vs. non-SS sicca, not done with a healthy control group.

*Wu et al*. [66]: Only pre-treatment data included.

- All relative abundance data compiled in Supplementary Data Sheet 2.

## Sequencing approaches and data processing

Supplementary Figure 5: 16S rRNA gene variable regions targeted for sequencing.

Distribution of studies according to the 16S rRNA gene variable regions sequenced in cohorts with systemic lupus erythematosus (SLE), SLE with primary Sjögren’s syndrome (SLE + pSS), and primary Sjögren’s syndrome (pSS). Bars represent the number of studies targeting each variable (V) region or region combination. Studies not reporting the amplified 16S rRNA gene region are indicated as “no information”. Created in Microsoft Word.

In some studies, there were other taxonomic analysis methods used:

**SLE:** Shotgun metagenome sequencing -> *Chen et al*. [18]

**SLE + pSS**: Shotgun metagenome sequencing profiling -> *Jia et al*. [46]

**pSS:** Deep RNA sequencing -> *Goodman et al*. [43]

## Taxonomic and statistical analysis methods

Supplementary Figure 6: Taxonomic profiling approaches used in included studies.

Distribution of studies according to the taxonomic analysis method applied for 16S rRNA gene sequencing data in cohorts with systemic lupus erythematosus (SLE), SLE with primary Sjögren’s syndrome (SLE + pSS), and primary Sjögren’s syndrome (pSS). Methods include operational taxonomic unit (OTU)–based clustering, amplicon sequence variant (ASV)–based inference, other taxonomic approaches, or not assigned (N/A). Bars indicate the number of studies using each method. Created in Microsoft Word.

**OTU vs. ASV-Picking:**

- **OTU:** 22 studies (10 SLE, 12 pSS) – mostly 97% similarity threshold
- **ASV:** 7 studies (5 SLE (*Liu et al*.[53]; *Azzouz et al* [39] [40]; *Toumi et al*. [60]; *Ling et al*. [52]) and 2 pSS (*Martínez-Nava et al*.[55] ; *Kim et al*. [47]))

Hybrid approach: *Azzouz et al.*[39] [40]) applied both OTU and ASV.

No data reported:

*Van der Meulen et al*. [63] (SLE + pSS), *Alan et al.* [38] and *Mandl et al*. [54] (pSS).

Statistical Analysis Software:

Most frequently used platforms were R (n = 20), SPSS (n = 16). Other programs that were used are LefSe, QIIME, and MOTHUR.

## Quality assessment

A structured quality assessment was conducted for every study addressing the following aspects:

- **Study design:** type of study, sample size, sampling method, power analysis
- **Representativeness:** race, gender, age, region, stage of disease
- **Data:** analysis method, level of data availability, information on medication
- **Conclusion:** plausibility of results and conclusions

Frequent points of critique: age, race, gender, matching variables, and sample size

- For pSS studies, symptom controls (non-SS sicca) were used (*Alam et al*. [38], *Moon et al*. [57], *Van der Meulen et al*. [62] [63], *Kim et al*. [47], *Mendez et al*. [56]), but only data comparing healthy controls vs. patients were included in the relative abundance table.

Details available in Supplementary Data Sheet 1.

## Medications

**Study distribution:**

- 9 studies: Treatment-naïve patients (no medication intake)
- 17 studies: Patients under medication treatment

Most reported medications:

- Hydroxychloroquine, antimalarials, prednisone, NSAIDs/NSAR, proton pump inhibitors (PPI), immunosuppressants, mycophenolate mofetil (MMF), methotrexate (MTX), azathioprine, xerogenic medication

# S2: Contacted corresponding authors of included studies

Corresponding authors of eligible studies were contacted by email to obtain quantitative microbiome data and relevant clinical or methodological metadata not reported in the original publications. Requested information included taxonomic abundance data, participant characteristics, methodological details, and derived indices (e.g., B/B (Bacillota/Bacteroidota) ratio). The date of initial contact and response status are reported where applicable. No reminder emails were sent.

Supplementary Table 2: Overview of data requests and author responses for included studies.

| Study | PMID | Date | Data requested | Response |
| --- | --- | --- | --- | --- |
| *Wei et al. 2019 [65]* | 30870437 | 27 Jan 2025 | Absolute abundance; relative abundance (species level) | No response |
| *Correa et al. 2017 [42]* | 28320468 | 30 Jan 2025 | Absolute abundance; relative abundance (phylum, family level) | Data received (5 Feb 2025) |
| *Chen et al. 2021 [18]* | 33124780 | 29 Jan 2025 | \|  \| \| --- \|  \| Absolute abundance; relative abundance (phylum, family, genus level) \| \| --- \| | No response |
| *Liu et al. 2021 [53]* | 34276643 | 31 Jan 2025 | Absolute abundance; relative abundance (family, genus level) | No response |
| *Azzouz et al. 2019 [39]* | 30782585 | 8 Feb 2025 | Absolute abundance; clinical metadata (age); matching variables | No response |
| *Li et al. 2020 [48]* | 32203722 | 11 Feb 2025 | Absolute abundance; clinical metadata (sex, medical history) | No response |
| *Li et al. 2019[50]* | 30872359 | 13 Feb 2025 | \|  \| \| --- \|  \| Absolute abundance; relative abundance; ethnicity \| \| --- \| | No response |
| *Toumi et al. 2022[60]* | 35983031 | 17 March 2025 | Absolute abundance; clinical metadata (age, sex) | No response |
| *Lian et al. 2024 [51]* | 38972998 | 19 March 2025 | Absolute abundance; relative abundance (phylum, family level); clinical metadata; disease state; medication; inclusion criteria; B/B ratio | No response |
| *Song et al. 2023 [59]* | 38169617 | 19 March 2025 | Absolute abundance; relative abundance (phylum, family, genus level); ethnicity; medication | No response |
| *Azzouz et al. 2023 [40]* | 37365013 | 20 March 2025 | Absolute abundance; relative abundance (phylum, family level); inclusion criteria; B/B ratio; ratio and age of controls | No response |
| *Ling et al. 2023 [52]* | 36969178 | 20 March 2025 | Absolute abundance; relative abundance (family level); inclusion criteria; B/B ratio | No response |
| *Guo et al. 2023 [44]* | 36755319 | 21 March 2025 | Absolute abundance; relative abundance (family level); B/B ratio | No response |
| *Jia et al. 2023 [46]* | 37120327 | 9 April 2025 | Absolute abundance; relative abundance (phylum, family, genus level); clinical metadata (age, sex); B/B ratio | No response |
| *Van der Meulen et al. 2019 [63]* | 30416033 | 10 April 2025 | Relative abundance (phylum, family, genus level);; biomarkers | No response |
| *Wu et al. 2019 [66]* | 31161440 | 13 April 2025 | Relative abundance (phylum, family, genus level);; biomarkers; medication | No response |
| *Alam et al. 2020 [38]* | 32208441 | 15 April 2025 | \|  \| \| --- \|  \| Relative abundance (phylum, family level);; biomarkers; B/B ratio; medication; taxonomic analysis method \| \| --- \| | No response |
| *Sharma et al. 2020 [58]* | 31514257 | 15 April 2025 | Relative abundance (phylum, family, genus level);; biomarkers; B/B ratio; age of controls | No response |
| *Moon et al. 2020 [57]* | 32059038 | 18 April 2025 | Biomarkers | No response |
| *Van der Meulen et al. 2018 [61]* | 30060225 | 23 April 2025 | Biomarkers; ethnicity (patients and controls); B/B ratio; recruiting hospital | No response |
| *Cano-Ortiz et al. 2020 [41]* | 33228011 | 24 April.2025 | Biomarkers; medication; B/B ratio | No response |
| *Kim et al. 2022 [47]* | 35603219 | 25 April 2025 | Ethnicity (patients and controls); B/B ratio | No response |
| *Mandl et al. 2017 [54]* | 29065905 | 27 April 2025 | Biomarkers; ethnicity (patients and controls); B/B ratio; relative abundance (phylum, family level); taxonomic and statistical analysis methods; alpha and beta diversity | Response, no data (28 April 2025) |
| *Van der Meulen et al. 2018 [62]* | 29572289 | 10 May 2025 | Ethnicity; inclusion and exclusion criteria; relative abundance (phylum level); B/B ratio | No response |
| *Yang et al. 2022 [68]* | 35634334 | 29 April 2025 | Relative abundance (phylum, family, genus level) | No response |
| *Zhou et al. 2018 [69]* | 30272305 | 1 May 2025 | \|  \| \| --- \|  \| B/B ratio \| \| --- \| | No response |
| *Li et al. 2016 [49]* | 27351333 | 11 May 2025 | B/B ratio; biomarkers | No response |
| *Xie et al. 2024 [67]* | 38602612 | 12 May 2025 | B/B ratio; biomarkers; ethnicity | No response |
| *Martinez-Nava et al. 2023 [55]* | 38079342 | 19 May 2025 | B/B ratio; ethnicity; sex of controls | Data received (22 May 2025) |
| *Goodman et al. 2023 [43]* | 36729650 | 20 May 2025 | Absolute abundance; B/B ratio; medication; biomarkers | No response |
| *Wang et al. 2023 [64]* | 36598587 | 21 May 2025 | Absolute abundance; B/B ratio; medication; biomarkers | No response |

Summary of data requests sent to corresponding authors of included studies, including publication details, date of contact, requested data types, and response status. Requested data comprised microbial abundance measures, clinical and demographic metadata, methodological information, and disease-related variables. Responses indicate whether requested data were received or no reply was obtained as of the last contact date. Created in Microsoft Word.

# S3: Quality assessment of included studies by the Newcastle–Ottawa Scale

The methodological quality of the included studies was assessed using an adapted version of the Newcastle–Ottawa Scale for cross-sectional studies. Study quality was evaluated across three domains: Selection, Comparability, and Outcome with a maximum total score of ten points. Quality assessment was performed by a single reviewer following predefined criteria. Detailed scores for each study are provided below. The original blank Newcastle-Ottawa Scale is also provided.

Supplementary Table 3: Methodological quality assessment of included studies.

| **Study** | **Selection (0-5)** | **Comparability (0-2)** | **Outcome (0-3)** | **Total score (0-10)** |
| --- | --- | --- | --- | --- |
| *Hevia et al. 2014 [45]* | 3 | 2 | 3 | **8** |
| *Wei et al. 2019 [65]* | 2 | 0 | 3 | **5** |
| *Correa et al. 2017 [42]* | 4 | 2 | 3 | **9** |
| *Chen et al. 2021 [18]* | 2 | 2 | 2 | **6** |
| *Liu et al. 2021 [53]* | 5 | 2 | 3 | **10** |
| *Azzouz et al. 2019 [39]* | 2 | 0 | 2 | **4** |
| *Li et al. 2020 [48]* | 1 | 2 | 3 | **6** |
| *Li et al. 2019 [50]* | 3 | 2 | 3 | **8** |
| *Toumi et al. 2022 [60]* | 4 | 2 | 2 | **8** |
| *Lian et al. 2024 [51]* | 3 | 0 | 3 | **6** |
| *Song et al. 2023 [59]* | 3 | 2 | 3 | **8** |
| *Azzouz et al. 2023 [40]* | 4 | 2 | 2 | **8** |
| *Ling et al. 2023 [52]* | 3 | 2 | 3 | **8** |
| *Guo et al. 2023 [44]* | 4 | 2 | 3 | **9** |
| *Jia et al. 2023 [46]* | 4 | 2 | 2 | **8** |
| *Van der Meulen et al. 2019 [63]* | 4 | 0 | 3 | **7** |
| *Wu et al. 2019 [66]* | 3 | 0 | 3 | **6** |
| *Alam et al. 2020 [38]* | 4 | 0 | 3 | **7** |
| *Sharma et al. 2020 [58]* | 4 | 0 | 2 | **6** |
| *Moon et al. 2020 [57]* | 3 | 0 | 3 | **6** |
| *Van der Meulen et al. 2018 [61]* | 4 | 0 | 3 | **7** |
| *Cano-Ortiz et al. 2020 [41]* | 3 | 2 | 3 | **8** |
| *Kim et al. 2022 [47]* | 4 | 0 | 2 | **6** |
| *Mandl et al. 2017 [54]* | 2 | 2 | 2 | **6** |
| *Mendez et al. 2020 [56]* | 2 | 0 | 3 | **5** |
| *Van der Meulen et al. 2018 [62]* | 3 | 0 | 3 | **6** |
| *Yang et al. 2022 [68]* | 3 | 2 | 3 | **8** |
| *Zhou et al. 2018 [69]* | 3 | 2 | 2 | **7** |
| *Li et al. 2016 [49]* | 3 | 2 | 3 | **8** |
| *Xie et al. 2024 [67]* | 2 | 0 | 3 | **5** |
| *Martinez-Nava et al. 2023 [55]* | 2 | 2 | 3 | **7** |
| *Goodman et al. 2023 [43]* | 2 | 2 | 3 | **7** |
| *Wang et al. 2023 [64]* | 3 | 2 | 2 | **7** |

Quality assessment of included studies based on the Newcastle–Ottawa Scale (NOS). Studies were evaluated across three domains: Selection (maximum 5 points), Comparability (maximum 2 points), and Outcome (maximum 3 points), with a total possible score of 10 points. Higher scores indicate better methodological quality. Created in Microsoft Word.

## 3.1 Newcastle-Ottawa Scale adapted for cross-sectional studies

**Selection:** (Maximum 5 stars (maximum of stars to reach per category resembled by * symbol))

1) Representativeness of the sample:

a) Truly representative of the average in the target population. * (all subjects or random sampling)

b) Somewhat representative of the average in the target population. * (non-random sampling)

c) Selected group of users.

d) No description of the sampling strategy.

2) Sample size:

a) Justified and satisfactory. *

b) Not justified.

3) Non-respondents:

a) Comparability between respondents and non-responders characteristics is established, and the response rate is satisfactory. *

b) The response rate is unsatisfactory, or the comparability between respondents and non-respondents is unsatisfactory.

c) No description of the response rate or the characteristics of the responders and the non-responders.

4) Ascertainment of the exposure (risk factor):

a) Validated measurement tool. **

b) Non-validated measurement tool, but the tool is available or described.*

c) No description of the measurement tool.

**Comparability:** (Maximum 2 stars)

1) The subjects in different outcome groups are comparable, based on the study design or analysis. Confounding factors are controlled.

a) The study controls for the most important factor (select one). *

b) The study control for any additional factor. *

**Outcome:** (Maximum 3 stars)

1) Assessment of the outcome:

a) Independent blind assessment. **

b) Record linkage. **

c) Self report. *

d) No description.

2) Statistical test:

a) The statistical test used to analyze the data is clearly described and appropriate, and the measurement of the association is presented, including confidence intervals and the probability level (p value). *

b) The statistical test is not appropriate, not described or incomplete

# S4: Taxonomic adjustments

All reported taxa in the included studies were adjusted to current NCBI taxonomy as of July 2025. Outdated taxonomic terms, that had to be reassigned to the correct phylum, family, genus, or species level are listed below. Taxonomic reassignments reflect nomenclature changes implemented by NCBI and do not imply biological reclassification within the original studies.

**Phylum**

Supplementary Table 4: Updated bacterial phylum nomenclature used in this study.

| Before taxonomic adjustment | After taxonomic adjustment |
| --- | --- |
| Actinobacteria | **Actinomycetota** |
| Verrucomicrobia | **Verrucomicrobiota** |
| Tenericutes | **Mycoplasmatota** |
| Firmicutes | **Bacillota** |
| Bacteroidetes | **Bacteroidota** |
| Proteobacteria | **Pseudomonadota** |
| Synergistetes | **Synergistota** |
| Fusobacteria | **Fusobacteriota** |
| Cyanobacteria | **Cyanobacteriota** |
| Spirochaetes | **Spirochaetota** |

Mapping of bacterial phylum names to their updated taxonomic designations according to current nomenclature standards. Original phylum names as reported in included studies (“Before taxonomic adjustment”) were harmonized to updated names (“After taxonomic adjustment”) to ensure consistency across analyses. Created in Microsoft Word.

**Family**

Supplementary Table 5: Updated bacterial family nomenclature used in this study.

| Before taxonomic adjustment | After taxonomic adjustment |
| --- | --- |
| Ruminococcaceae | **Oscillospiraceae** |
| Clostridiae | **Clostridiaceae** |
| Xanthomonadaceae | **Lysobacteraceae** |

Mapping of bacterial family names to their updated taxonomic designations according to current nomenclature standards. Original family names as reported in included studies (“Before taxonomic adjustment”) were harmonized to updated names (“After taxonomic adjustment”) to ensure consistency across analyses. Created in Microsoft Word.

**Genus**

Supplementary Table 6: Updated bacterial genus nomenclature used in this study.

| Before taxonomic adjustment | After taxonomic adjustment |
| --- | --- |
| *Mitsuaria* | ***Roseateles*** |

Mapping of bacterial genus names to their updated taxonomic designations according to current nomenclature standards. Original genus names as reported in included studies (“Before taxonomic adjustment”) were harmonized to updated names (“After taxonomic adjustment”) to ensure consistency across analyses. Created in Microsoft Word.

**Species**

Supplementary Table 7: Updated bacterial species nomenclature used in this study.

| Before taxonomic adjustment | After taxonomic adjustment |
| --- | --- |
| *Actinomyces cardiffensis* | ***Schaalia cardiffensis*** |
| *Actinomyces naeslundii* | ***Actinomyces oris*** |
| *Atopobium pavulum* | ***Lancefieldella parvula*** |
| *Atopobium rimae* | ***Lancefieldella rimae*** |
| *Clostridium boltae* | ***Enterocloster boltae*** |
| *Clostridium aldenense* | ***Enterocloster aldenensis*** |
| *Clostridium ramorum* | ***Thomasclavelia ramosa*** |
| *Clostridium bartlettii* | ***Intestinibacter bartlettii*** |
| *Eubacterium rectale* | ***Agathobacter rectalis*** |
| *Eubacterium hallii* | ***Anaerobutyricum hallii*** |
| *Eubacterium eligens* | ***Lachnospira eligens*** |
| *Eubacterium biforme* | ***Holdemanella biformis*** |
| *Lactobacillus fermentum* | ***Limosilactobacillus fermentum*** |
| *Lactobacillus mucosae* | ***Limosilactobacillus mucosae*** |
| *Lactobacillus oris* | ***Limosilactobacillus oris*** |
| *Lactobacillus reutei* | ***Limosilactobacillus reutei*** |
| *Lactobacillus ruminis* | ***Ligilactobacillus ruminis*** |
| *Lactobacillus sakei* | ***Latilactobacillus sakei*** |
| *Lactobacillus salivarius* | ***Ligilactobacillus salivarius*** |
| *Lactobacillus vaginalis* | ***Limosilactobacillus vaginalis*** |
| *Ruminococcus gnavus* | ***Mediterraneibacter gnavus*** |
| *Ruminococcus obeum* | ***Blautia obeum*** |
| *Streptococcus oligofermentans* | ***Streptococcus cristatus*** |
| *Streptococcus tigurinus* | ***Streptococcus oralis*** |
| *Bacteroides coprocola* | ***Phocaeicola coprocola*** |
| *Bacteroides dorei* | ***Phocaeicola dorei*** |
| *Bacteroides plebeius* | ***Phocaeicola plebeius*** |
| *Bacteroides vulgatus* | ***Phocaeicola vulgatus*** |
| *Prevotella copri* | ***Segatella copri*** |
| *Prevotella enoeca* | ***Hoylesella enoeca*** |
| *Prevotella nanceiensis* | ***Hoylesella nanceiensis*** |
| *Prevotella oris* | ***Segatella oris*** |
| *Prevotella oulorum* | ***Segatella oulorum*** |
| *Prevotella pleuritidis* | ***Hoylesella pleuritidis*** |
| *Prevotella salivae* | ***Segatella salivae*** |
| *Prevotella shahii* | ***Hoylesella shahii*** |
| *Prevotella stercorea* | ***Leyella stercorea*** |
| *Halomonas hamiltonii* | ***Vreelandella hamiltonii*** |

Mapping of bacterial species names to their updated taxonomic designations according to current nomenclature standards. Original species names as reported in included studies (“Before taxonomic adjustment”) were harmonized to updated names (“After taxonomic adjustment”) to ensure consistency across analyses. Created in Microsoft Word.
