# Supplementary material for: Coordinated oral–gut microbiota relocation in connective tissue diseases: a systematic review
Source: Front Immunol. 2026 Jul 3;17:1841874. doi: 10.3389/fimmu.2026.1841874 (PMC13376073; doi:10.3389/fimmu.2026.1841874)

Study-level findings of relative abundance of gut and oral microbes at the phylum level

Legend:

Significantly increased (p<0,05)	⇑
Increased (trend)	↑
Significantly decreased (p<0,05)	⇓
Decreased (trend)	↓
No significant difference	=
Presumption supported by data on different levels	*
Comparing patients with HC	

Systemic lupus erythematosus

Phylum		Actinomycetota (Actinobacteria)	Bacillota (Firmicutes)	Bacteroidota (Bacteroidetes)	Campylobacterota	Cyanobacteriota (Cyanobacteria)	Fusobacteriota (Fusobacteria)	Mycoplasmatota (Tenericutes)	Pseudomonadota (Proteobacteria)	Spirochaetota (Spirochaetes)	Synergistota (Synergistetes)	Thermodesulfobacteria	Verrucomicrobiota (Verrucomicrobia)
Oral	*Correa et al 2017 [42]*												
	Liu et al. 2021 [53]	⇓	↓	↑			↑		↑				
	Li et al. 2020 [48]	⇓				⇑		⇓	* ↓				
	Guo et al. 2023 [44]	⇓	⇑	⇑	⇑	⇑	⇑		⇓				
Gut	*Hevia et al. 2014 [45]*	=	⇓	⇑		=	=		↓				=
	Wei et al. 2019 [65]		* ↓						⇑				
	Chen et al. 2021 [18]												
	Liu et al. 2021 [53]	↑	↓	↓				↑	↓				
	Azzouz et al. 2019 [39]	=	=	=			=		↓				↑
	Li et al. 2019 [50]	=	=	=			⇑	⇓	=		=		=
	Toumi et al. 2022 [60]							⇓					
	Lian et al. 2024 [51]												
	Song et al. 2023 [59]												
	Azzouz et al. 2023 [40]												
	Ling et al. 2023 [52]	↑	↑	⇓			↑		↓				
	Jia et al. 2023 [46]												
	Van der Meulen et al. 2019 [63]	↓	↓	⇑					⇑				=

Sjögren's syndrome

Phylum		Actinomycetota (Actinobacteria)	Bacillota (Firmicutes)	Bacteroidota (Bacteroidetes)	Campylobacterota	Cyanobacteriota (Cyanobacteria)	Fusobacteriota (Fusobacteria)	Mycoplasmatota (Tenericutes)	Pseudomonadota (Proteobacteria)	Spirochaetota (Spirochaetes)	Synergistota (Synergistetes)	Thermodesulfobacteria	Verrucomicrobiota (Verrucomicrobia)	
Oral	*Alam et al. 2020 [38]*	=	⇑	↑			↓		⇓	⇓				
	Sharma et al. 2020 [58]													
	Van der Meulen et al. 2018 [61]	↑	↑	=			=		⇓					
	Kim et al. 2022 [47]													1
	Van der Meulen et al. 2018 [62]													
	Zhou et al. 2018 [69]	=	⇑	⇑			=		⇓					
	Li et al. 2016 [49]	↓	↑			=			⇓	↓				
	Xie et al. 2024 [67]	↑	=	=	↑		↑		⇓					
	Martinez-Nava et al. 2023 [55]	=		=	↑				↓	↓	↓			
Gut	*Jia et al. 2023 [46]*	↓	↑						↑				=	
	Van der Meulen et al. 2019 [63]	↓	↓	⇑					⇑					
	Wu et al. 2019 [66]	⇑	⇑	⇓		=	⇑	⇓	⇑					2
	Moon et al. 2020 [57]	⇓	↓	⇑					↑					
	Cano-Ortiz et al. 2020 [41]	⇓	⇓	⇑				=	⇑					
	Mandl et al. 2017 [54]													
	Mendez et al. 2020 [56]	↑	↓	↑					↑					
	Yang et al. 2022 [68]	↑	↓	↓			↑		↑				↑	
	Goodman et al. 2023 [43]													
	Wang et al. 2023 [64]			* ↓					⇑					

1 no data due to comparison between pSS and non SS sicca

2 data only on patients pre treatment vs. HC

Study-level findings of relative abundance of gut and oral microbes at the family level

Phylum	Actinomycetota (Actinobacteria)								
Family / Systemic lupus erythematosus	Actinomycetaceae	Atopobiaceae	Bifidobacteriaceae	Coriobacteriaceae	Corynebacteriaceae	Eggerthellaceae	Microbacteriaceae	Micrococcaceae	Streptomycetaceae
**Oral**									
Correa et al 2017 [42]									
Liu et al. 2021 [53]									
Li et al. 2020 [48]					↓			↓	
Guo et al. 2023 [44]	↓							↓	
**Gut**									
Hevia et al. 2014 [45]			=						
Wei et al. 2019 [65]									
Chen et al. 2021 [18]									
Liu et al. 2021 [53]			↑	↑					
Azzouz et al. 2019 [39]			=						
Li et al. 2019 [50]									
Toumi et al. 2022 [60]									
Lian et al. 2024 [51]									
Song et al. 2023 [59]									
Azzouz et al. 2023 [40]									
Ling et al. 2023 [52]									
Jia et al. 2023 [46]									
Van der Meulen et al. 2019 [63]									

Phylum	Bacillota (Firmicutes)																					
Family / Systemic lupus erythematosus	Acidaminococcaceae	Aerococcaceae	Carnobacteriaceae	Catabacteraceae	Clostridiaceae	Coprobacillaceae	Defluviitaleacaea	Enterococcaceae	Erysipelotrichaceae	Eubacteriaceae	Filifactoraceae	Gemellaceae	Lachnospiraceae	Lactobacillaceae	Oscillospiraceae (Ruminococcaceae)	Paenibacillaceae	Peptoniphilaceae	Peptostreptococcaceae	Selenomonadaceae	Streptococcaceae	Vallitaleaceae	Veillonellaceae
**Oral**																						
Correa et al 2017 [42]																						
Liu et al. 2021 [53]												↓										
Li et al. 2020 [48]							↓							↑				↓				↑
Guo et al. 2023 [44]													↑					↓		↓		↑
**Gut**																						
Hevia et al. 2014 [45]				↓					=				↓		↓							↑
Wei et al. 2019 [65]					↓										↓							
Chen et al. 2021 [18]																						
Liu et al. 2021 [53]													↓		↓							
Azzouz et al. 2019 [39]					=				↑				↑		↓					↑		↑
Li et al. 2019 [50]														↑						↑		
Toumi et al. 2022 [60]																						
Lian et al. 2024 [51]																						
Song et al. 2023 [59]																						
Azzouz et al. 2023 [40]																						
Ling et al. 2023 [52]																	*↑					
Jia et al. 2023 [46]																						
Van der Meulen et al. 2019 [63]																						

Phylum	Bacteroidota (Bacteroidetes)									Campylobacterota	Cyanobacteriota (Cyanobacteria)	Fusobacteriota (Fusobacteria)		Mycoplasmatota (Tenericutes)
Family / Systemic lupus erythematosus	Bacteroidaceae	Barnesiellaceae	Flavobacteriaceae	Odoribacteraceae	Porphyromonadaceae	Prevotellaceae	Rikenellaceae	Tannerellaceae	Weeksellaceae	Campylobacteraceae		Fusobacteriaceae	Leptotrichiaceae	Mycoplasmataceae
**Oral**														
Correa et al 2017 [42]														
Liu et al. 2021 [53]														
Li et al. 2020 [48]														
Guo et al. 2023 [44]					↓	↑				↑			↑	
**Gut**														
Hevia et al. 2014 [45]					=	↑	↑							
Wei et al. 2019 [65]						↓								
Chen et al. 2021 [18]														
Liu et al. 2021 [53]														
Azzouz et al. 2019 [39]	=				=	↑	↓							
Li et al. 2019 [50]														
Toumi et al. 2022 [60]								↑						
Lian et al. 2024 [51]														
Song et al. 2023 [59]														
Azzouz et al. 2023 [40]														
Ling et al. 2023 [52]														
Jia et al. 2023 [46]														
Van der Meulen et al. 2019 [63]														

Phylum	Pseudomonadota (Proteobacteria)																Spirochaetota (Spirochaetes)	Synergistota (Synergistetes)	Thermodesulfobacteria		Verrucomicrobiota (Verrucomicrobia)	
Family / Systemic lupus erythematosus	Acetobacteraceae	Brucellaceae	Burkholderiaceae	Caulobacteraceae	Comamodaceae	Enterobacteriaceae	Halomonadaceae	Hyphomicrobiaceae	Lysobacteraceae (Xanthomonadaceae)	Moraxellaceae	Neisseriaceae	Oxalobacteraceae	Pasteurellaceae	Roseobacteraceae	Sphaerotilaceae	Sphingomonadaceae	Treponemataceae	Dethiosulfovibrionaceae	Bilophilacea	Desulfovibrionaceae	Akkermansiaceae	Verrucomicrobiaceae
**Oral**																						
Correa et al 2017 [42]																						
Liu et al. 2021 [53]																						
Li et al. 2020 [48]							↓	↓	↓	↑	*↓					*↓						
Guo et al. 2023 [44]						↑	↓				=		↓			↑						
**Gut**																						
Hevia et al. 2014 [45]																				↓		
Wei et al. 2019 [65]						↑																
Chen et al. 2021 [18]																						
Liu et al. 2021 [53]						↓																
Azzouz et al. 2019 [39]																						↑
Li et al. 2019 [50]																						
Toumi et al. 2022 [60]																						
Lian et al. 2024 [51]																						
Song et al. 2023 [59]																						
Azzouz et al. 2023 [40]																						
Ling et al. 2023 [52]																						
Jia et al. 2023 [46]																						
Van der Meulen et al. 2019 [63]																						

Study-level findings of relative abundance of gut and oral microbes at the family level

Phylum	Actinomycetota (Actinobacteria)								
Family / Sjögren's syndrome	Actinomycetaceae	Atopobiaceae	Bifidobacteriaceae	Coriobacteriaceae	Corynebacteriaceae	Eggerthellaceae	Microbacteriaceae	Micrococcaceae	Streptomyceatceae
**Oral**									
Alam et al. 2020 [38]									
Sharma et al. 2020 [58]		* ↑	* ↑						
Van der Meulen et al. 2018 [61]			↑	↑					
Kim et al. 2022 [47]									
Van der Meulen et al. 2018 [62]									
Zhou et al. 2018 [69]				↑					
Li et al. 2016 [49]									
Xie et al. 2024 [67]	↑								
Martinez-Nava et al. 2023 [55]									
**Gu**									
Jia et al. 2023 [46]									
Van der Meulen et al. 2019 [63]									
Wu et al. 2019 [66]									
Moon et al. 2020 [57]									
Cano-Ortiz et al. 2020 [41]			↓						
Mandl et al. 2017 [54]									
Mendez et al. 2020 [56]	↑			↑		↑			
Yang et al. 2022 [68]									
Goodman et al. 2023 [43]			* ↑						
Wang et al. 2023 [64]									

Phylum	Bacillota (Firmicutes)																					
Family / Sjögren's syndrome	Acidaminococcaceae	Aerococcaceae	Carnobacteriaceae	Catabacteraceae	Clostridiaceae	Coprobacillaceae	Defluviitaleacea	Enterococcaceae	Erysipelotrichaceae	Eubacteriaceae	Filifactoraceae	Gemellaceae	Lachnospiraceae	Lactobacillaceae	Oscillospiraceae (Ruminococcaceae)	Paenibacillaceae	Peptoniphilaceae	Peptostreptococcaceae	Selenomonadaceae	Streptococcaceae	Vallitaleaceae	Veillonellaceae
**Oral**																						
Alam et al. 2020 [38]																						
Sharma et al. 2020 [58]														* ↑					* ↑			
Van der Meulen et al. 2018 [61]		↓	↓					↓						↑	↓		↑					* ↑
Kim et al. 2022 [47]																						
Van der Meulen et al. 2018 [62]																						
Zhou et al. 2018 [69]																						
Li et al. 2016 [49]																						
Xie et al. 2024 [67]																			↑	↓		↑
Martinez-Nava et al. 2023 [55]																						
**Gu**																						
Jia et al. 2023 [46]					↑														↓			
Van der Meulen et al. 2019 [63]															* ↑							
Wu et al. 2019 [66]													↓		↑							↑
Moon et al. 2020 [57]					↓								* ↓									
Cano-Ortiz et al. 2020 [41]					↑								↓		↓			=				↑
Mandl et al. 2017 [54]																						
Mendez et al. 2020 [56]					↓					↑			↓	↑	↓							* ↑
Yang et al. 2022 [68]													↓		↓							
Goodman et al. 2023 [43]																						
Wang et al. 2023 [64]	↓									↓				↑								

Phylum	Bacteroidota (Bacteroidetes)									Campylobacterota	Cyanobacteriota (Cyanobacteria)	Fusobacteriota (Fusobacteria)		Mycoplasmatota (Tenericutes)
Family / Sjögren's syndrome	Bacteroidaceae	Barnesiellaceae	Flavobacteriaceae	Odoribacteraceae	Porphyromonadaceae	Prevotellaceae	Rikenellaceae	Tannerellaceae	Weeksellaceae	Campylobacteraceae		Fusobacteriaceae	Leptotrichiaceae	Mycoplasmataceae
**Oral**														
Alam et al. 2020 [38]														
Sharma et al. 2020 [58]													↓	
Van der Meulen et al. 2018 [61]														
Kim et al. 2022 [47]														
Van der Meulen et al. 2018 [62]														
Zhou et al. 2018 [69]														
Li et al. 2016 [49]														
Xie et al. 2024 [67]						↑				↑		↑	↑	
Martinez-Nava et al. 2023 [55]													↑	
**Gu**														
Jia et al. 2023 [46]														
Van der Meulen et al. 2019 [63]														
Wu et al. 2019 [66]	↑													
Moon et al. 2020 [57]														
Cano-Ortiz et al. 2020 [41]	↓			↑	↓	↑	↑							
Mandl et al. 2017 [54]														
Mendez et al. 2020 [56]														
Yang et al. 2022 [68]	↑					↓								
Goodman et al. 2023 [43]														
Wang et al. 2023 [64]					↓		↓							

Phylum	Pseudomonadota (Proteobacteria)																Spirochaetota (Spirochaetes)	Synergistota (Synergistetes)	Thermodesulfobacteria		Verrucomicrobiota (Verrucomicrobia)	
Family / Sjögren's syndrome	Acetobacteraceae	Brucellaceae	Burkholderiaceae	Caulobacteraceae	Comamodaceae	Enterobacteriaceae	Halomonadaceae	Hyphomicrobiaceae	Lysobacteraceae (Xanthomonadaceae)	Moraxellaceae	Neisseriaceae	Oxalobacteraceae	Pasteurellaceae	Roseobacteraceae	Sphaerotilaceae	Sphingomonadaceae	Treponemataceae	Dethiosulfovibrionaceae	Bilophiliacea	Desulfovibrionaceae	Akkermansiaceae	Verrucomicrobiaceae
**Oral**																						
Alam et al. 2020 [38]																						
Sharma et al. 2020 [58]													* ↓									
Van der Meulen et al. 2018 [61]			↓								↓		↓									
Kim et al. 2022 [47]																						
Van der Meulen et al. 2018 [62]																						
Zhou et al. 2018 [69]			↓								↓											
Li et al. 2016 [49]																						
Xie et al. 2024 [67]	↓		↓								↓		↓									
Martinez-Nava et al. 2023 [55]																						
**Gu**																						
Jia et al. 2023 [46]																						
Van der Meulen et al. 2019 [63]																						
Wu et al. 2019 [66]						↑																
Moon et al. 2020 [57]																						
Cano-Ortiz et al. 2020 [41]						↑																
Mandl et al. 2017 [54]																						
Mendez et al. 2020 [56]																					↑	
Yang et al. 2022 [68]						↑																
Goodman et al. 2023 [43]																						
Wang et al. 2023 [64]						↑						↓								↓		

Study-level findings of relative abundance of gut and oral microbes at the genus level

Systemic lupus erythematosus

Phylum	Actinomycetota (Actinobacteria)																		
Family	Actinomycetaceae			Atopobiaceae			Bifidobacteriaceae					Coriobacteriaceae	Corynebacteriaceae	Eggerthellaceae			Microbacteriaceae	Micrococaceae	Streptomyceatceae
Genus	Actinomyces	Mobiluncus	Schaalia	Atopobium	Lancefieldella	Olsenella	Alloscardovia	Bifidobacterium	Gardnerella	Parascardovia	Scardovia	Collinsella	Corynebacterium	Adlercreutzia	Eggerthella	Gordonibacter	Leucobacter	Rothia	
**Oral**																			
Correa et al 2017 [42]																		↓	
Liu et al. 2021 [53]	↓																	↓	
Li et al. 2020 [48]		↓											↓					↓	
Guo et al. 2023 [44]																		↓	
**Gut**																			
Hevia et al. 2014 [45]																			
Wei et al. 2019 [65]																			
Chen et al. 2021 [18]	*↑				*↑	*↑		*↑			*↑		*↑					*↑	
Liu et al. 2021 [53]								↓											
Azzouz et al. 2019 [39]								=											
Li et al. 2019 [50]								↓											
Toumi et al. 2022 [60]																			
Lian et al. 2024 [51]																			
Song et al. 2023 [59]																			
Azzouz et al. 2023 [40]																			
Ling et al. 2023 [52]				↑					↑										
Jia et al. 2023 [46]																			
Van der Meulen et al. 2019 [63]	↓											↓							

Phylum	Bacillota (Firmicutes)										
Family	Acidaminococcaceae	Aerococcaceae	Carnobacteriaceae	Clostridiaceae	Coprobacillaceae	Defluviitaleceaea	Enterococcaceae	Erysipelotrichaceae	Eubacteriaceae	Filifactoraceae	Gemellaceae
Genus	Phascolarctobacterium	Abiotrophia	Granulicatella	Clostridium	Thomasclavelia		Enterococcus	Dielma	Eubacterium	Filifactor	Gemella
**Oral**											
Correa et al 2017 [42]											
Liu et al. 2021 [53]			↓								
Li et al. 2020 [48]										↓	
Guo et al. 2023 [44]			↓								
**Gut**											
Hevia et al. 2014 [45]											
Wei et al. 2019 [65]											
Chen et al. 2021 [18]							*↑				
Liu et al. 2021 [53]	↓										
Azzouz et al. 2019 [39]											
Li et al. 2019 [50]											
Toumi et al. 2022 [60]											
Lian et al. 2024 [51]											
Song et al. 2023 [59]											
Azzouz et al. 2023 [40]											
Ling et al. 2023 [52]	↓										
Jia et al. 2023 [46]											
Van der Meulen et al. 2019 [63]											

Phylum	Bacillota (Firmicutes)																	
Family	Lachnospiraceae																	
Genus	Agathobacter	Anaerobutyricum	Blautia	Coprococcus	Dorea	Enterocloster	Johnsonella	Lachnoanaerobaculum	Lachnoclostridium	Lachnospira	Mediterraneibacter	Otoolea	Oribacterium	Roseburia	Shuttleworthella	Solobacterium	Stomatobaculum	Tyzzerella
**Oral**																		
Correa et al 2017 [42]										↑								
Liu et al. 2021 [53]																		
Li et al. 2020 [48]			↑										↓					
Guo et al. 2023 [44]								↑							↓			
**Gut**																		
Hevia et al. 2014 [45]																		
Wei et al. 2019 [65]														↓				
Chen et al. 2021 [18]				*↓										*↓				
Liu et al. 2021 [53]			↑															
Azzouz et al. 2019 [39]			↑		=													
Li et al. 2019 [50]			↑											↓				
Toumi et al. 2022 [60]																		
Lian et al. 2024 [51]														↓				
Song et al. 2023 [59]			*↑											*↓				
Azzouz et al. 2023 [40]																		
Ling et al. 2023 [52]																		
Jia et al. 2023 [46]											*↑							
Van der Meulen et al. 2019 [63]			=		=					↑								

Phylum	Bacillota (Firmicutes)																							
Family	Lactobacillaceae					Oscillospiraceae (Ruminococcaceae)		Peptoniphiliaceae				Peptostreptococcaceae		Selenomonadaceae				Streptococcaceae	Vallitaleaceae	Veillonellaceae				
Genus	Lactobacillus	Latilactobacillus	Ligilactobacillus	Limosilactobacillus	Weissenella	Faecalibacterium	Ruminococcus	Anaerococcus	Finegoldia	Parvimonas	Peptophilus	Peptostreptococcus	Romboutsia	Megamonas	Mitsuokella	Schwartzia	Selenomonas	Streptococcus	Vallitalea	Allisonella	Anaeroglobus	Dialister	Megasphaera	Veillonella
**Oral**																								
Correa et al 2017 [42]																	↑	↓						
Liu et al. 2021 [53]																	↑	↓						↑
Li et al. 2020 [48]	↑									↓								↓				↓		↑
Guo et al. 2023 [44]												↓						↓						↑
**Gut**																								
Hevia et al. 2014 [45]																								
Wei et al. 2019 [65]							↓											↑						
Chen et al. 2021 [18]				*↑														*↑						*↑
Liu et al. 2021 [53]	↑					↓								↓				↑						↑
Azzouz et al. 2019 [39]							↑											↑				↑	↑	
Li et al. 2019 [50]	↑					↓												↑					↑	
Toumi et al. 2022 [60]																								
Lian et al. 2024 [51]							↓							↓						↓				
Song et al. 2023 [59]																								
Azzouz et al. 2023 [40]																								↑
Ling et al. 2023 [52]	↑					↓		↑	↑	↑				↓				↑				↓		
Jia et al. 2023 [46]																								*↑
Van der Meulen et al. 2019 [63]													↓					↑				↓		

Study-level findings of relative abundance of gut and oral microbes at the genus level

Systemic lupus erythematosus

Phylum	Bacteroidota (Bacteroidetes)																	
Family	Bacteroidaceae		Barnesiellaceae		Flavobacteriaceae	Odoribacteraceae	Porphyromonadaceae	Prevotellaceae						Rikenellaceae	Tannerellaceae		Weeksellaceae	
Genus	Bacteroides	Phocaeicola	Barnesiella	Coprobacter	Capnocytophaga	Odoribacter	Porphyromonas	Alloprevotella	Hoylesella	Leyella	Paraprevotella	Prevotella	Segatella	Alistipes	Parabacteroides	Tannerella	Bergeyella	Chryseobacterium
**Oral**																		
Correa et al 2017 [42]												↑	*↑					
Liu et al. 2021 [53]																		
Li et al. 2020 [48]			↑									*↑				↓		↓
Guo et al. 2023 [44]							↓	↑				↑						
**Gut**																		
Hevia et al. 2014 [45]	↑																	
Wei et al. 2019 [65]							↓				↓	↓						
Chen et al. 2021 [18]	*↓																	
Liu et al. 2021 [53]	↓											↑						
Azzouz et al. 2019 [39]	=																	
Li et al. 2019 [50]																		
Toumi et al. 2022 [60]														↑	↑			
Lian et al. 2024 [51]	↑																	
Song et al. 2023 [59]																		
Azzouz et al. 2023 [40]	*↑																	
Ling et al. 2023 [52]	↓											↓						
Jia et al. 2023 [46]																		
Van der Meulen et al. 2019 [63]	↑	*↑	↑											↑				

Phylum	Campylobacterota	Cyanobacteria (Cyanobacteria)	Fusobacteriota (Fusobacteria)			Mycoplasmato (Tenericut
Family	Campylobacteraceae		Fusobacteriaceae	Leptotrichiaceae		Mycoplasmataceae
Genus	Campylobacter		Fusobacterium	Leptotrichia	Sneathia	Mycoplasma
**Oral**						
Correa et al 2017 [42]						
Liu et al. 2021 [53]			↑			
Li et al. 2020 [48]						↓
Guo et al. 2023 [44]				↑		
**Gut**						
Hevia et al. 2014 [45]						
Wei et al. 2019 [65]						
Chen et al. 2021 [18]						
Liu et al. 2021 [53]					↑	
Azzouz et al. 2019 [39]						
Li et al. 2019 [50]	↑					
Toumi et al. 2022 [60]						
Lian et al. 2024 [51]						
Song et al. 2023 [59]						
Azzouz et al. 2023 [40]			↑			
Ling et al. 2023 [52]				↑		
Jia et al. 2023 [46]						
Van der Meulen et al. 2019 [63]				↑		

Phylum	Pseudomonadota (Proteobacteria)																												
Family	Acetobacteraceae	Brucellaceae	Burkholderiaceae		Caulobacteraceae	Comamodaceae			Enterobacteriaceae				Halomonadaceae		Hyphomicrobiaceae	Lysobacteraceae	Moraxellaceae	Neisseriaceae			Oxalobacteraceae	Pasteurellaceae			Pseudomonadaceae	Roseobacteraceae	Sphaerotilaceae	Sphingomonadaceae	
Genus	Acetobacter	Pseudochrobact	Lautropia	Ralstonia	Caulobacter	Delftia	Comamonas	Limnohabitans	Citrobacter	Enterobacter	Escherichia	Klebsiella	Halomonas	Vreelandella			Acinetobacter	Eikenella	Kingella	Neisseria		Actinobacillus	Haemophilus	Mannheimia	Pseudomonas	Octadecabacter	Roseateles (Mitsuaria)	Novosphingobium	Sphingomonas
**Oral**																													
Correa et al 2017 [42]																							↓		↑				↓
Liu et al. 2021 [53]			↑																	↓			↓						
Li et al. 2020 [48]					↓								↓				↓	↓	↓									↓	↓
Guo et al. 2023 [44]													↓							=			↓						↑
**Gut**																													
Hevia et al. 2014 [45]																													
Wei et al. 2019 [65]																													
Chen et al. 2021 [18]																													
Liu et al. 2021 [53]																													
Azzouz et al. 2019 [39]																													
Li et al. 2019 [50]																													
Toumi et al. 2022 [60]																													
Lian et al. 2024 [51]																													
Song et al. 2023 [59]																													
Azzouz et al. 2023 [40]																													
Ling et al. 2023 [52]										↓																			
Jia et al. 2023 [46]																													
Van der Meulen et al. 2019 [63]																													

Phylum	Spirochaetota (Spirochaetes)	Synergistota (Synergistetes)		Thermodesulfobacteria		Verrucomicrobiota (Verrucomicrobia)	
Family	Treponemataceae	Aminobacteriaceae	Dethiosulfovibrionaceae	Desulfovibrionaceae		Akkermansiaceae	Verrucomicrobiaceae
Genus	Treponema	Fretibacterium	Pyramidobacter	Bilophila	Desulfovibrio	Akkermansia	
**Oral**							
Correa et al 2017 [42]	↑	↑					
Liu et al. 2021 [53]							
Li et al. 2020 [48]	*↓		↑				
Guo et al. 2023 [44]							
**Gut**							
Hevia et al. 2014 [45]					↓		
Wei et al. 2019 [65]							
Chen et al. 2021 [18]							
Liu et al. 2021 [53]							
Azzouz et al. 2019 [39]						↑	
Li et al. 2019 [50]							
Toumi et al. 2022 [60]							
Lian et al. 2024 [51]							
Song et al. 2023 [59]							
Azzouz et al. 2023 [40]							
Ling et al. 2023 [52]							
Jia et al. 2023 [46]							
Van der Meulen et al. 2019 [63]							

Study-level findings of relative abundance of gut and oral microbes at the genus level

Sjögren's syndrome	Actinomyces	Mobiluncus	Schaalia	Atopobium	Lancefieldella	Olsenella	Alloscardovia	Bifidobacterium	Gardnerella	Parascardovia	Scardovia	Collinsella	Corynebacterium	Adlercreutzia	Eggerthella	Gordonibacter	Leucobacter	Rothia	
Phylum	Actinomycetota (Actinobacteria)																		
Family	Actinomycetaceae			Atopobiaceae			Bifidobacteriaceae					Coriobacteriaceae	Corynebacteriaceae	Eggerthellaceae			Microbacteriaceae	Micrococcaceae	Streptomyceataceae
**Oral**																			
Alam et al. 2020 [38]				↑															
Sharma et al. 2020 [58]				↑		↑		↑			↑								
Van der Meulen et al. 2018 [61]				↑			↑	↑											
Kim et al. 2022 [47]																			
Van der Meulen et al. 2018 [62]																		=	
Zhou et al. 2018 [69]	↑																		
Li et al. 2016 [49]																	↑		
Xie et al. 2024 [67]	↑												↑						
Martinez-Nava et al. 2023 [55]	↑																		
**Gut**																			
Jia et al. 2023 [46]								*↑											
Van der Meulen et al. 2019 [63]	↓							↓				↓							
Wu et al. 2019 [66]								↑											
Moon et al. 2020 [57]								↓				↓							
Cano-Ortiz et al. 2020 [41]								↓				↑							
Mandl et al. 2017 [54]								↓											
Mendez et al. 2020 [56]												↓							
Yang et al. 2022 [68]																			
Goodman et al. 2023 [43]								↑		↑									
Wang et al. 2023 [64]											↑	↓				↓			

Sjögren's syndrome	Phascolarctobacterium	Abiotrophia	Granulicatella	Clostridium	Thomasclavelia		Enterococcus	Dielma	Eubacterium	Filifactor	Gemella	Agathobacter	Anaerobutyricum	Blautia	Coprococcus	Dorea	Enterocloster	Johnsonella	Lachnoanaerobaculum	Lachnoclostridium	Lachnospira	Mediterraneibacter	Otoolea	Oribacterium	Roseburia	Shuttleworthella	Solobacterium	Stomatobaculum	Tyzzerella
Phylum	Bacillota (Firmicutes)																												
Family	Acidaminococcaceae	Aerococcaceae	Carnobacteriaceae	Clostridiaceae	Coprobacillaceae	Defluviitalecaea	Enterococcaceae	Erysipelotrichaceae	Eubacteriaceae	Filifactoraceae	Gemellaceae	Lachnospiraceae																	
**Oral**																													
Alam et al. 2020 [38]												↑																	
Sharma et al. 2020 [58]		↓																											
Van der Meulen et al. 2018 [61]		↓	↓				↓				↑																		
Kim et al. 2022 [47]																													
Van der Meulen et al. 2018 [62]																													
Zhou et al. 2018 [69]																													
Li et al. 2016 [49]			↓																										
Xie et al. 2024 [67]																													
Martinez-Nava et al. 2023 [55]																								↑					
**Gut**																													
Jia et al. 2023 [46]																													
Van der Meulen et al. 2019 [63]														=		=					↑								
Wu et al. 2019 [66]				↓																									
Moon et al. 2020 [57]												↓		↓		↓													
Cano-Ortiz et al. 2020 [41]				↑			↑							↓		↓					↓				↓				
Mandl et al. 2017 [54]																													
Mendez et al. 2020 [56]																													
Yang et al. 2022 [68]				↓			↑																						
Goodman et al. 2023 [43]				↑					↓					=											=				
Wang et al. 2023 [64]	↓								↓																				

Sjögren's syndrome	Lactobacillus	Latilactobacillus	Ligilactobacillus	Limosilactobacillus	Weissella	Faecalibacterium	Ruminococcus	Anaerococcus	Finegoldia	Parvimonas	Peptophilus	Peptostreptococcus	Romboutsia	Megamonas	Mitsuokella	Schwartzia	Selenomonas	Streptococcus	Vallitalea	Allisonella	Anaeroglobus	Dialister	Megasphaera	Veillonella
Phylum	Bacillota (Firmicutes)																							
Family	Lactobacillaceae					Oscillospiraceae (Ruminococcaceae)		Peptoniphilaceae				Peptostreptococcaceae		Selenomonadaceae				Streptococcaceae	Vallitaleaceae	Veillonellaceae				
**Oral**																								
Alam et al. 2020 [38]				↑														↑						
Sharma et al. 2020 [58]	↑			↑												↑	↑					↑		
Van der Meulen et al. 2018 [61]	↑						↓			↑		↑						↓		↑		↑		↑
Kim et al. 2022 [47]																							=	
Van der Meulen et al. 2018 [62]				↑													↑	↓						↑
Zhou et al. 2018 [69]	↓											↑												
Li et al. 2016 [49]																								
Xie et al. 2024 [67]	=																	↓						↑
Martinez-Nava et al. 2023 [55]	↓																↓	↑						↑
**Gut**																								
Jia et al. 2023 [46]							*↓																	*↑
Van der Meulen et al. 2019 [63]						↑	↑						↓					↑				↓		
Wu et al. 2019 [66]						↑																		
Moon et al. 2020 [57]	↑					↓																		↑
Cano-Ortiz et al. 2020 [41]						↓	↓											↑						
Mandl et al. 2017 [54]																								
Mendez et al. 2020 [56]						↓																↑	↑	
Yang et al. 2022 [68]						↓												↑				↓	↑	↑
Goodman et al. 2023 [43]	↑			*↑		=							↑											↑
Wang et al. 2023 [64]	↑		*↑				↓							↓				*↑						↑

Study-level findings of relative abundance of gut and oral microbes at the genus level

Phylum	Bacteroidota (Bacteroidetes)																	
Family	Bacteroidaceae		Barnesiellaceae		Flavobacteriaceae	Odoribacteraceae	Porphyromonadaceae	Prevotellaceae						Rikenellaceae	Tannerellaceae		Weeksellaceae	
Sjögren's syndrome / Genus	Bacteroides	Phocaeicola	Barnesiella	Coprobacter	Capnocytophaga	Odoribacter	Porphyromonas	Alloprevotella	Hoylesella	Leyella	Paraprevotella	Prevotella	Segatella	Alistipes	Parabacteroides	Tannerella	Bergeyella	Chryseobacterium
**Oral**																		
Alam et al. 2020 [38]					* ↓							↑						
Sharma et al. 2020 [58]																		
Van der Meulen et al. 2018 [61]								↓										↓
Kim et al. 2022 [47]																		
Van der Meulen et al. 2018 [62]																		
Zhou et al. 2018 [69]												↑						
Li et al. 2016 [49]																		
Xie et al. 2024 [67]								↑				↑						
Martinez-Nava et al. 2023 [55]							↑					↑						
**Gut**																		
Jia et al. 2023 [46]													* ↓					
Van der Meulen et al. 2019 [63]	↑	* ↑	↑											↑				
Wu et al. 2019 [66]	↑											↑						
Moon et al. 2020 [57]	↑													=				
Cano-Ortiz et al. 2020 [41]	↓											↑		↓	↓			
Mandl et al. 2017 [54]														↓				
Mendez et al. 2020 [56]												↑						

Phylum	Campylobacterota	Cyanobacterota (Cyanobacteria)	Fusobacteriota (Fusobacteria)			Mycoplasmato (Tenericut
Family	Campylobacteraceae		Fusobacteriaceae	Leptotrichiaceae		Mycoplasmataceae
Sjögren's syndrome / Genus	Campylobacter		Fusobacterium	Leptotrichia	Sneathia	Mycoplasma
**Oral**						
Alam et al. 2020 [38]	↓		↓	* ↓		
Sharma et al. 2020 [58]				↓		
Van der Meulen et al. 2018 [61]			=	=		
Kim et al. 2022 [47]						
Van der Meulen et al. 2018 [62]			↑			
Zhou et al. 2018 [69]						
Li et al. 2016 [49]						
Xie et al. 2024 [67]	↑		↑	↑		
Martinez-Nava et al. 2023 [55]			↑			
**Gut**						
Jia et al. 2023 [46]						
Van der Meulen et al. 2019 [63]						
Wu et al. 2019 [66]						
Moon et al. 2020 [57]						
Cano-Ortiz et al. 2020 [41]						
Mandl et al. 2017 [54]						
Mendez et al. 2020 [56]						

Phylum	Pseudomonadota (Proteobacteria)																												
Family	Acetobacteraceae	Brucellaceae	Burkholderiaceae		Caulobacteraceae	Comamodaceae			Enterobacteriaceae				Halomonadaceae		Hyphomicrobiaceae	Lysobacteraceae	Moraxellaceae	Neisseriaceae			Oxalobacteraceae	Pasteurellaceae			Pseudomonadaceae	Rosebacteraceae	Sphaerotilaceae	Sphingomonadaceae	
Sjögren's syndrome / Genus	Acetobacter	Pseudochrobact	Lautropia	Ralstonia	Caulobacter	Delftia	Comamonas	Limnohabitans	Citrobacter	Enterobacter	Escherichia	Klebsiella	Halomonas	Vreelandella			Acinetobacter	Eikenella	Kingella	Neisseria		Actinobacillus	Haemophilus	Mannheimia	Pseudomonas	Octadecabacter	Roseateles (Mitsuaria)	Novosphingobium	Sphingomonas
**Oral**																													
Alam et al. 2020 [38]																				↓			↓						
Sharma et al. 2020 [58]																						↓		↓					
Van der Meulen et al. 2018 [61]			↓																	↓			↓						
Kim et al. 2022 [47]																													
Van der Meulen et al. 2018 [62]																							↓						
Zhou et al. 2018 [69]																				↓									
Li et al. 2016 [49]		↑		↑		↓	↑	↓												↓			↓				↑		
Xie et al. 2024 [67]	↓																			↓			↓						
Martinez-Nava et al. 2023 [55]																													
**Gut**																													
Jia et al. 2023 [46]																													
Van der Meulen et al. 2019 [63]																													
Wu et al. 2019 [66]																													
Moon et al. 2020 [57]																													
Cano-Ortiz et al. 2020 [41]											↑																		
Mandl et al. 2017 [54]																													
Mendez et al. 2020 [56]																													

Phylum	Spirochaetota (Spirochaetes)	Synergistota (Synergistetes)		Thermodesulfobacteria		Verrucomicrobiota (Verrucomicrobia)	
Family	Treponemataceae	Aminobacteriaceae	Dethiosulfovibrionaceae	Desulfovibrionaceae		Akkermansiaceae	Verrucomicrobiaceae
Sjögren's syndrome / Genus	Treponema	Fretibacterium	Pyramidobacter	Bilophila	Desulfovibrio	Akkermansia	
**Oral**							
Alam et al. 2020 [38]							
Sharma et al. 2020 [58]							
Van der Meulen et al. 2018 [61]							
Kim et al. 2022 [47]							
Van der Meulen et al. 2018 [62]							
Zhou et al. 2018 [69]							
Li et al. 2016 [49]							
Xie et al. 2024 [67]							
Martinez-Nava et al. 2023 [55]							
**Gut**							
Jia et al. 2023 [46]							
Van der Meulen et al. 2019 [63]							
Wu et al. 2019 [66]							
Moon et al. 2020 [57]							
Cano-Ortiz et al. 2020 [41]							
Mandl et al. 2017 [54]							
Mendez et al. 2020 [56]							

Study-level findings of relative abundance of gut and oral microbes at the species level

Systemic lupus erythematosus

**Phylum**	**Actinomycetota** (Actinobacteria)																																				
**Family**	**Actinomycetacea**								**Atopobiaceae**					**Bifidobacteriaceae**													**Coriobacteriaceae**	**Corynebacteriaceae**			**Eggerthellaceae**			**Microbacteriaceae**	**Micrococcaceae**		**Streptomyceatceae**
**Genus**	**Actinomyces**						**Mobiluncus**	**Schaalia**	**Atopobium**	**Lancefieldella**		**Olsenella**		**Alloscardovia**	**Bifidobacterium**								**Gardnerella**	**Parascardovia**	**Scardovia**		**Collinsella**	**Corynebacterium**			**Adlercreutzia**	**Gordonibacter**	**Eggerthella**	**Leucobacter**	**Rothia**		
**Species**	Actinomyces graevenitzii	Actinomyces johnsonii	Actinomyces massiliensis	Actinomyces oris	Actinomyces timonensis	Actinomyces viscosus		Schaalia cardiffensis		Lancefieldella parvula	Lancefieldella rimae	Olsenella profusa	Olsenella uli	Alloscardovia omnicolens	Bifidobacterium adolescentis	Bifidobacterium animalis	Bifidobacterium bifidum	Bifidobacterium breve	Bifidobacterium catenulatum	Bifidobacterium dentium	Bifidobacterium longum	Bifidobacterium scardovii	Gardnerella vaginalis	Parascardovia denticolens	Scardovia inopinata	Scardovia wiggsiae	Collinsella aerofaciens	Corynebacterium durum	Corynebacterium matruchotii	Corynebacterium propinquum	Adlercreutzia equolifaciens	Gordonibacter pamelaeae	Eggerthella lenta		Rothia aeria	Rothia dentocariosa	
**Oral**																																					
Correa et al 2017 [42]																																			↓		
Liu et al. 2021 [53]																																					
Li et al. 2020 [48]																												↓	↓								
Guo et al. 2023 [44]																																					
**Gut**																																					
Hevia et al. 2014 [45]																																					
Wei et al. 2019 [65]																																					
Chen et al. 2021 [18]	↑	↑	↑	↑	↑	↑		↑		↑	↑	↑	↑	↑				↑		↑			↑	↑	↑	↑	↓	↑		↑	↑		↑		↑	↑	
Liu et al. 2021 [53]															↓						↓																
Azzouz et al. 2019 [39]															=																						
Li et al. 2019 [50]																																					
Toumi et al. 2022 [60]																																					
Lian et al. 2024 [51]																																					
Song et al. 2023 [59]																																					
Azzouz et al. 2023 [40]																																					
Ling et al. 2023 [52]																																					
Jia et al. 2023 [46]																				↑							↓				↑						
Van der Meulen et al. 2019 [63]																																					

Study-level findings of relative abundance of gut and oral microbes at the species level

	Phylum	Bacillota (Firmicutes)																										
	Family	Acidaminococcaceae	Aerococcaceae	Carnobacteriaceae	Coprobacillaceae	Clostridiaceae	Defluviitaleacaea	Enterococcaceae		Erysipelotrichaceae		Eubacteriaceae			Filifactoraceae	Gemellaceae	Lachnospiraceae											
	Genus	Phascolarctobacterium	Abiotrophia	Granulicatella	Thomoclavelia	Clostridium		Enterococcus		Dielma	Holdemanella	Eubacterium			Filifactor	Gemella	Agathobacter	Anaerobutyricum	Blautia			Coprococcus			Dorea	Enterocloster		
Systemic lupus erythematosus	Species		Abiotrophia defectiva	Granulicatella elegans	Thomoclavelia ramosa	Clostridium perfringens		Enterococcus avium	Enterococcus casseliflavus	Dielma fastidiosa	Holdemanella biformis	Eubacterium limosum	Eubacterium ruminatum	Eubacterium ventriosum	Filifactor alocis	Gemella morbillorum	Agathobacter rectalis	Anaerobutyricum hallii	Blautia faecis	Blautia obeum	Blautia wexlerae	Coprococcus catus	Coprococcus comes	Coprococcus eutactus	Dorea longicatena	Enterocloster aldenensis	Enterocloster boltae	Enterocloster clostridiformis
Oral	Correa et al 2017 [42]																											
	Liu et al. 2021 [53]																											
	Li et al. 2020 [48]														↓													
	Guo et al. 2023 [44]																											
Gut	Hevia et al. 2014 [45]																											
	Wei et al. 2019 [65]																											
	Chen et al. 2021 [18]		↑	↑	↑			↑	↑			↑		↓		↑	↓	↑		↓		↓	↓		↓			
	Liu et al. 2021 [53]																											
	Azzouz et al. 2019 [39]																											
	Li et al. 2019 [50]																											
	Toumi et al. 2022 [60]																											
	Lian et al. 2024 [51]																											
	Song et al. 2023 [59]																	↑ (light)	↑ (light)		↑ (light)				↑ (light)	↑ (light)		
	Azzouz et al. 2023 [40]																											
	Ling et al. 2023 [52]																											
	Jia et al. 2023 [46]				↑																		↓					
	Van der Meulen et al. 2019 [63]																											

	Phylum																														
	Family																	Lactobacillaceae													
	Genus	Johnsonella	Lachnoanaerobaculum -	Lachnoclostridium	Lachnospira	Mediterraneibacter			Otoolea	Oribacterium	Roseburia			Shuttleworthella	Solobacterium	Stomatobaculum	Tyzzerella	Lactobacillus					Latilactobacillus	Ligilactobacillus		Limosilactobacillus					Weissenella
	Species	Johnsonella ignava	Lachnoanaerobaculum orale	Clostridium scindens	Lachnospira eligens	Mediterraneibacter gnavus	Ruminococcus lactaris	Ruminococcus torques	Clostridium symbiosum	Oribacterium asaccharolyticum	Roseburia hominis	Roseburia intestinalis	Roseburia inulinivorans	Shuttleworthella satelles		Stomatobaculum longum	Clostridium nexile	Lactobacillus crispatus	Lactobacillus delbrueckii	Lactobacillus gasseri	Lactobacillus iners	Lactobacillus johnsonii	Latilactobacillus sakei	Ligilactobacillus ruminis	Ligilactobacillus salivarius	Limosilactobacillus fermentum	Limosilactobacillus mucosae	Limosilactobacillus oris	Limosilactobacillus reuteri	Limosilactobacillus vaginalis	
Oral	Correa et al 2017 [42]																														
	Liu et al. 2021 [53]																														
	Li et al. 2020 [48]	↓																									↑				
	Guo et al. 2023 [44]																														
Gut	Hevia et al. 2014 [45]																														
	Wei et al. 2019 [65]																														
	Chen et al. 2021 [18]			↑	↓	↑	↓	↑			↓	↓	↓	↑			↑	↑	↓	↑					↑		↑		↑		
	Liu et al. 2021 [53]																				↑										
	Azzouz et al. 2019 [39]					↑																									
	Li et al. 2019 [50]																										↑				
	Toumi et al. 2022 [60]																														
	Lian et al. 2024 [51]																														
	Song et al. 2023 [59]				↓ (light)							↓ (light)	↓ (light)																		
	Azzouz et al. 2023 [40]					↑ (light)																									
	Ling et al. 2023 [52]																														
	Jia et al. 2023 [46]					↑		↑				↓													↑						
	Van der Meulen et al. 2019 [63]																														

	Phylum																
	Family	Oscillospiraceae (Ruminococcaceae)			Peptoniphilaceae				Peptostreptococcaceae			Selenomonadaceae					
	Genus	Faecalibacterium	Ruminococcus		Anaerococcus	Finegoldia	Peptophilus	Parvimonas	Intestinibacter	Peptostreptococcus	Romboutsia	Megamonas		Mitsuokella -	Schwartzia	Selenomonas	
	Species	Faecalibacterium prausnizii	Ruminococcus bromii	Ruminococcus callidus					Intestinibacter bartlettii			Megamonas hypermegale	Megamonas rupellensis	Mitsuokella multacida		Selenomonas noxia	Selenomonas ruminatum
Oral	Correa et al 2017 [42]															↑ (light)	
	Liu et al. 2021 [53]																
	Li et al. 2020 [48]																
	Guo et al. 2023 [44]																
Gut	Hevia et al. 2014 [45]																
	Wei et al. 2019 [65]																
	Chen et al. 2021 [18]	↓		↓					↑				↓	↓			
	Liu et al. 2021 [53]																
	Azzouz et al. 2019 [39]																
	Li et al. 2019 [50]	↓															
	Toumi et al. 2022 [60]																
	Lian et al. 2024 [51]																
	Song et al. 2023 [59]	↓ (light)															
	Azzouz et al. 2023 [40]																
	Ling et al. 2023 [52]																
	Jia et al. 2023 [46]	↓		↓					↑								
	Van der Meulen et al. 2019 [63]																

	Phylum																							
	Family	Streptococcaceae													Vallitaleaceae	Veillonellaceae								
	Genus	Streptococcus													Vallitalea	Anaeroglobus	Dialister		Megasphaera	Veillonella				
	Species	Streptococcus anginosus	Streptococcus australis	Streptococcus constellatus	Streptococcus cristatus	Streptococcus infantis	Streptococcus intermedius	Streptococcus lutetiensis	Streptococcus mutans	Streptococcus oralis	Streptococcus parasanguinis	Streptococcus sanguinis	Streptococcus sobrinus	Streptococcus suis	Vallitalea pronyensis	Anaeroglobus geminatus	Dialister invisus	Dialister succinatiphilus	Megasphera micronuciformis	Veillonella atypica	Veillonella dispar	Veillonella parvula	Veillonella rodentium	Veillonella rogosae
Oral	Correa et al 2017 [42]											↓ (light)				↑ (light)								
	Liu et al. 2021 [53]	↓																						
	Li et al. 2020 [48]																↓							
	Guo et al. 2023 [44]																							
Gut	Hevia et al. 2014 [45]																							
	Wei et al. 2019 [65]																							
	Chen et al. 2021 [18]	↑		↑	↑		↑	↑		↑	↑						↑		↑		↑	↑		
	Liu et al. 2021 [53]																							
	Azzouz et al. 2019 [39]																							
	Li et al. 2019 [50]	↑																			↑			
	Toumi et al. 2022 [60]																							
	Lian et al. 2024 [51]																							
	Song et al. 2023 [59]														↓ (light)			↓ (light)						
	Azzouz et al. 2023 [40]																							
	Ling et al. 2023 [52]																							
	Jia et al. 2023 [46]	↑	↓						↑		↑										↑	↑		
	Van der Meulen et al. 2019 [63]																							

Study-level findings of relative abundance of gut and oral microbes at the species level

Systemic lupus erythematosus

Phylum	Family	Genus	Species	Oral: Correa et al 2017 [42]	Oral: Liu et al. 2021 [53]	Oral: Li et al. 2020 [48]	Oral: Guo et al. 2023 [44]	Gut: Hevia et al. 2014 [45]	Gut: Wei et al. 2019 [65]	Gut: Chen et al. 2021 [18]	Gut: Liu et al. 2021 [53]	Gut: Azzouz et al. 2019 [39]	Gut: Li et al. 2019 [50]	Gut: Toumi et al. 2022 [60]	Gut: Lian et al. 2024 [51]	Gut: Song et al. 2023 [59]	Gut: Azzouz et al. 2023 [40]	Gut: Ling et al. 2023 [52]	Gut: Jia et al. 2023 [46]	Gut: Van der Meulen et al. 2019 [63]
Bacteroidota (Bacteroidetes)	Bacteroidaceae	Bacteroides	Bacteroides caccae																	
			Bacteroides cellulosilyticus							↓ (dark red)										
			Bacteroides clarus							↓ (dark red)										
			Bacteroides eggerthii							↓ (dark red)										
			Bacteroides fragilis							↑ (dark green)		↑ (light green)				↑ (light green)			↑ (dark green)	
			Bacteroides intestinalis							↓ (dark red)										
			Bacteroides ovatus																	↑ (dark green)
			Bacteroides salyersiae							↓ (dark red)										
			Bacteroides thetaiotaomicron																	↑ (dark green)
			Bacteroides uniformis									↓ (dark red)								↑ (dark green)
		Phocaeicola	Phocaeicola coprocola																	
			Phocaeicola dorei																	↑ (light green)
			Phocaeicola plebeius							↓ (dark red)									↓ (dark red)	↑ (light green)
			Phocaeicola vulgatus							↑ (dark green)										↑ (dark green)
	Barnesiellaceae	Barnesiella	Barnesiella intestinihominis																	
		Coprobacter	Coprobacter fastidiosus							↓ (dark red)										
	Flavobacteriaceae	Capnocytophaga	Capnocytophaga gingivalis	↓ (light red)																
			Capnocytophaga leadbetteri																	
	Odoribacteraceae	Odoribacter	Odoribacter splanchnicus																	
	Porphyromonadaceae	Porphyromonas	Porphyromonas gingivalis																	
	Prevotellaceae	Alloprevotella																		
		Hoylesella	Hoylesella enoeca			↑ (dark green)														
			Hoylesella nanceiensis			↓ (dark red)														
			Hoylesella pleuritidis	↑ (light green)		↑ (light green)														
			Hoylesella shahii																	
		Leyella	Leyella stercorea																	
		Paraprevotella	Paraprevotella clara							↓ (dark red)									↓ (dark red)	
		Prevotella	Prevotella genomosp			↑ (light green)														
			Prevotella histicola																	
			Prevotella melaninogenica																	
			Prevotella micans			↑ (light green)														
			Prevotella nigrescens	↑ (light green)																
		Segatella	Segatella copri							↓ (dark red)		↑ (light green)								
			Segatella oris	↑ (light green)																
			Segatella oulorum	↑ (light green)																
			Segatella salivae																	
			Alistipes finegoldii							↑ (dark green)										
			Alistipes onderdonkii							↑ (dark green)				↑ (dark green)						
			Alistipes putredinis																	
			Alistipes senegalensis																	
	Rikenellaceae	Alistipes	Alistipes shahii							↓ (dark red)										
	Tannerellaceae	Parabacteroides	Parabacteroides distasonis																	
			Parabacteroides merdae							↓ (dark red)										
		Tannerella																		
	Weeksellaceae	Bergeyella																		
		Chryseobacterium	Chryseobacterium taiwanense			↓ (dark red)														
Campylobacterota	Campylobacteraceae	Campylobacter	Campylobacter coli																	
			Campylobacter concisus																	
			Campylobacter gracilis																	
			Campylobacter showae																	
Cyanobacteriota (Cyanobacteria)																				

Study-level findings of relative abundance of gut and oral microbes at the species level

Systemic lupus erythematosus

		Species: Fusobacterium nucleatum	Fusobacterium periodonticum	Fusobacterium ulcerans	Fusobacterium varium	Leptotrichia hofstadii	Leptotrichia hongkongensis	Leptotrichia wadei	Sneathia amnii	Mycoplasma ovis
Phylum		Fusobacteriota (Fusobacteria)								Mycoplasmatota (Tenericutes)
Family		Fusobacteriaceae				Leptotrichiaceae				Mycoplasmataceae
Genus		Fusobacterium				Leptotrichia			Sneathia	Mycoplasma
Oral	Correa et al 2017 [42]									
	Liu et al. 2021 [53]									
	Li et al. 2020 [48]									↓
	Guo et al. 2023 [44]									
Gut	Hevia et al. 2014 [45]									
	Wei et al. 2019 [65]									
	Chen et al. 2021 [18]	↑								
	Liu et al. 2021 [53]									
	Azzouz et al. 2019 [39]									
	Li et al. 2019 [50]									
	Toumi et al. 2022 [60]									
	Lian et al. 2024 [51]									
	Song et al. 2023 [59]									
	Azzouz et al. 2023 [40]									
	Ling et al. 2023 [52]									
	Jia et al. 2023 [46]									
	Van der Meulen et al. 2019 [63]									

		Acetobacter	Pseudochrobactrum	Lautropia mirabilis	Ralstonia	Caulobacter	Delftia	Comamonas testosteroni	Limnohabitans	Citrobacter koseri	Enterobacter	Escherichia coli	Escherichia fergusonii	Klebsiella pneumoniae	Halomonas	Vreenlandella hamiltonii	Hyphomicrobiaceae	Lysobacteraceae	Acinetobacter
Phylum		Pseudomonadota (Proteobacteria)																	
Family		Acetobacteraceae	Brucellaceae	Burkholderiaceae		Caulobacteraceae	Comamodaceae			Enterobacteriaceae					Halomonadaceae		Hyphomicrobiaceae	Lysobacteraceae	Moraxellaceae
Genus		Acetobacter	Pseudochrobactrum	Lautropia	Ralstonia	Caulobacter	Delftia	Comamonas	Limnohabitans	Citrobacter	Enterobacter	Escherichia		Klebsiella	Halomonas	Vreenlandella			Acinetobacter
Oral	Correa et al 2017 [42]																		
	Liu et al. 2021 [53]																		
	Li et al. 2020 [48]							↑											
	Guo et al. 2023 [44]																		
Gut	Hevia et al. 2014 [45]																		
	Wei et al. 2019 [65]																		
	Chen et al. 2021 [18]											↑							
	Liu et al. 2021 [53]																		
	Azzouz et al. 2019 [39]																		
	Li et al. 2019 [50]																		
	Toumi et al. 2022 [60]																		
	Lian et al. 2024 [51]																		
	Song et al. 2023 [59]												↑						
	Azzouz et al. 2023 [40]																		
	Ling et al. 2023 [52]																		
	Jia et al. 2023 [46]																		
	Van der Meulen et al. 2019 [63]																		

		Eikenella corrodens	Kingella	Neisseria elongata	Neisseria oralis	Neisseria perflava	Neisseria subflava	Oxalobacteraceae	Actinobacillus	Haemophilus haemolyticus	Haemophilus influenzae	Haemophilus parainfluenzae	Haemophilus sputorum	Mannheimia	Pseudomonas aeruginosa	Pseudomonas graminis	Octadecabacter arcticus	Roseateles	Novosphingobium	Sphingomonas
Phylum		Pseudomonadota (Proteobacteria)																		
Family		Neisseriaceae						Oxalobacteraceae	Pasteurellaceae						Pseudomonadaceae		Roseobacteraceae	Sphaerotilaceae	Sphingomonadaceae	
Genus		Eikenella	Kingella	Neisseria					Actinobacillus	Haemophilus				Mannheimia	Pseudomonas		Octadecabacter	Roseateles	Novosphingobium	Sphingomonas
Oral	Correa et al 2017 [42]											↓								
	Liu et al. 2021 [53]																			
	Li et al. 2020 [48]															↓				
	Guo et al. 2023 [44]																			
Gut	Hevia et al. 2014 [45]																			
	Wei et al. 2019 [65]																			
	Chen et al. 2021 [18]	↑									↑									
	Liu et al. 2021 [53]																			
	Azzouz et al. 2019 [39]																			
	Li et al. 2019 [50]																			
	Toumi et al. 2022 [60]																			
	Lian et al. 2024 [51]																			
	Song et al. 2023 [59]																			
	Azzouz et al. 2023 [40]																			
	Ling et al. 2023 [52]																			
	Jia et al. 2023 [46]																			
	Van der Meulen et al. 2019 [63]																			

		Treponema denticola	Treponema lecithinolyticum	Treponema maltophilum	Fretibacterium fastidiosum	Pyramidobacter piscolens	Bilophila wadsworthia	Desulfovibrio legallii	Desulfovibrio piger	Akkermansia muciniphila	
Phylum		Spirochaetota (Spirochaetes)			Synergistota (Synergistetes)		Thermodesulfobacteria			Verrucomicrobiota (Verrucomicrobia)	
Family		Treponemataceae			Aminobacteriaceae	Dethiosulfovibrionaceae	Desulfovibrionaceae			Akkermansiaceae	Verrucomicrobiaceae
Genus		Treponema			Fretibacterium	Pyramidobacter	Bilophila	Desulfovibrio		Akkermansia	
Oral	Correa et al 2017 [42]			↑	↑						
	Liu et al. 2021 [53]										
	Li et al. 2020 [48]	↓	↓			↑					
	Guo et al. 2023 [44]										
Gut	Hevia et al. 2014 [45]										
	Wei et al. 2019 [65]										
	Chen et al. 2021 [18]									↑	
	Liu et al. 2021 [53]										
	Azzouz et al. 2019 [39]									=	
	Li et al. 2019 [50]										
	Toumi et al. 2022 [60]										
	Lian et al. 2024 [51]										
	Song et al. 2023 [59]										
	Azzouz et al. 2023 [40]										
	Ling et al. 2023 [52]										
	Jia et al. 2023 [46]						↓				
	Van der Meulen et al. 2019 [63]										

Study-level findings of relative abundance of gut and oral microbes at the species level

	Phylum	Actinomycetota (Actinobacteria)																																				
	Family	Actinomycetacea								Atopobiaceae					Bifidobacteriaceae													Coriobacteriaceae	Corynebacteriaceae			Eggerthellaceae			Microbacteriaceae	Micrococcaceae		Streptomyceatceae
	Genus	Actinomyces						Mobiluncus	Schaalia	Atopobium	Lancefieldella		Olsenella		Alloscardovia	Bifidobacterium								Gardnerella	Parascardovia	Scardovia		Collinsella	Corynebacterium			Adlercreutzia	Gordonibacter	Eggerthella	Leucobacter	Rothia		
Sjögren’s syndrome	Species	Actinomyces graevenitzii	Actinomyces johnsonii	Actinomyces massiliensis	Actinomyces oris	Actinomyces timonensis	Actinomyces viscosus		Schaalia cardiffensis		Lancefieldella parvula	Lancefieldella rimae	Olsenella profusa	Olsenella uli	Alloscardovia omnicolens	Bifidobacterium adolescentis	Bifidobacterium animalis	Bifidobacterium bifidum	Bifidobacterium breve	Bifidobacterium catenulatum	Bifidobacterium dentium	Bifidobacterium longum	Bifidobacterium scardovii	Gardnerella vaginalis	Parascardovia denticolens	Scardovia inopinata	Scardovia wiggsiae	Collinsella aerofaciens	Corynebacterium durum	Corynebacterium matruchotii	Corynebacterium propinquum	Adlercreutzia equolifaciens	Gordonibacter pamelaeae	Eggerthella lenta		Rothia aeria	Rothia dentocariosa	
Oral	Alam et al. 2020 [38]										↑																			↓						↓		
	Sharma et al. 2020 [58]																																					
	Van der Meulen et al. 2018 [61]																																					
	Kim et al. 2022 [47]																																					
	Van der Meulen et al. 2018 [62]																																					
	Zhou et al. 2018 [69]																																					
	Li et al. 2016 [49]																																					
	Xie et al. 2024 [67]																																					
	Martinez-Nava et al. 2023 [55]																																					
Gut	Jia et al. 2023 [46]															↑					↑							↓				↑						
	Van der Meulen et al. 2019 [63]																																					
	Wu et al. 2019 [66]																																					
	Moon et al. 2020 [57]															=		=				↓																
	Cano-Ortiz et al. 2020 [41]																																					
	Mandl et al. 2017 [54]																																					
	Mendez et al. 2020 [56]																																					
	Yang et al. 2022 [68]																																					
	Goodman et al. 2023 [43]																↑			↑	↑		↑		↑													
	Wang et al. 2023 [64]																										↑						↓					

Study-level findings of relative abundance of gut and oral microbes at the species level

	Phylum	Bacillota (Firmicutes)																							
	Family	Acidaminococcaceae	Aerococcaceae	Carnobacteriaceae	Coprobacillaceae	Clostridiaceae	Defluviitaleacea	Enterococcaceae		Erysipelotrichaceae		Eubacteriaceae			Filifactoraceae	Gemellaceae	Lachnospiraceae								
	Genus	Phascolarctobacterium	Abiotrophia	Granulicatella	Thomasclavelia	Clostridium		Enterococcus		Dielma	Holdemanella	Eubacterium			Filifactor	Gemella	Agathobacter	Anaerobutyricum	Blautia			Coprococcus			Dorea
Sjögren's syndrome	Species		Abiotrophia defectiva	Granulicatella elegans	Thomasclavelia ramosa	Clostridium perfringens		Enterococcus avium	Enterococcus casseliflavus	Dielma fastidiosa	Holdemanella biformis	Eubacterium limosum	Eubacterium ruminatum	Eubacterium ventriosum	Filifactor alocis	Gemella morbillorum	Agathobacter rectalis	Anaerobutyricum hallii	Blautia faecis	Blautia obeum	Blautia wexlerae	Coprococcus catus	Coprococcus comes	Coprococcus eutactus	Dorea longicatena
Oral	Alam et al. 2020 [38]																								
	Sharma et al. 2020 [58]																								
	Van der Meulen et al. 2018 [61]																								
	Kim et al. 2022 [47]																								
	Van der Meulen et al. 2018 [62]																								
	Zhou et al. 2018 [69]																								
	Li et al. 2016 [49]																								
	Xie et al. 2024 [67]																								
	Martinez-Nava et al. 2023 [55]																								
Gut	Jia et al. 2023 [46]				↑	↑					↓											↑	↓		
	Van der Meulen et al. 2019 [63]																								
	Wu et al. 2019 [66]																								
	Moon et al. 2020 [57]																	↓							
	Cano-Ortiz et al. 2020 [41]																								↓
	Mandl et al. 2017 [54]																								
	Mendez et al. 2020 [56]																								
	Yang et al. 2022 [68]																↑								
	Goodman et al. 2023 [43]																								
	Wang et al. 2023 [64]									↓														↓	

	Phylum																								
	Family																			Lactobacillaceae					
	Genus	Enterocloster			Johnsonella	Lachnoanaerobaculum -	Lachnoclostridium	Lachnospira	Mediterraneibacter			Otoolea	Oribacterium	Roseburia			Shuttleworthella	Solobacterium	Stomatobaculum	Tyzzerella	Lactobacillus				
Sjögren's syndrome	Species	Enterocloster aldenensis	Enterocloster boltae	Enterocloster clostridiformis	Johnsonella ignava	Lachnoanaerobaculum orale	Clostridium scindens	Lachnospira eligens	Mediterraneibacter gnavus	Ruminococcus lactaris	Ruminococcus torques	Clostridium symbiosum	Oribacterium asaccharolyticu	Roseburia hominis	Roseburia intestinalis	Roseburia inulinivorans	Shuttleworthella satelles		Stomatobaculum longum	Clostridium nexile	Lactobacillus crispatus	Lactobacillus delbrueckii	Lactobacillus gasseri	Lactobacillus iners	Lactobacillus johnsonii
Oral	Alam et al. 2020 [38]					↓							↓						↓						
	Sharma et al. 2020 [58]																								
	Van der Meulen et al. 2018 [61]																								
	Kim et al. 2022 [47]																								
	Van der Meulen et al. 2018 [62]																								
	Zhou et al. 2018 [69]																								
	Li et al. 2016 [49]																								
	Xie et al. 2024 [67]																								
	Martinez-Nava et al. 2023 [55]																								
Gut	Jia et al. 2023 [46]		↑					↓	↑		↓	↑			↓										
	Van der Meulen et al. 2019 [63]																								
	Wu et al. 2019 [66]																								
	Moon et al. 2020 [57]																								
	Cano-Ortiz et al. 2020 [41]			↑					=	↓															
	Mandl et al. 2017 [54]																								
	Mendez et al. 2020 [56]																								
	Yang et al. 2022 [68]																								
	Goodman et al. 2023 [43]																						↑		↑
	Wang et al. 2023 [64]																						↑		

	Phylum																								
	Family										Oscillospiraceae (Ruminococcaceae)			Peptoniphiliaceae				Peptostreptococcaceae			Selenomonadaceae				
	Genus	Latilactobacillus	Ligilactobacillus		Limosilactobacillus					Weissenella	Faecalibacterium	Ruminococcus		Anaerococcus	Finegoldia	Peptophilus	Parvimonas	Intestinibacter	Peptostreptococcus	Romboutsia	Megamonas		Mitsuokella -	Schwartzia	Selenomonas
Sjögren's syndrome	Species	Latilactobacillus sakei	Ligilactobacillus ruminis	Ligilactobacillus salivarius	Limosilactobacillus fermentum	Limosilactobacillus mucosae	Limosilactobacillus oris	Limosilactobacillus reuteri	Limosilactobacillus vaginalis		Faecalibacterium prausnizii	Ruminococcus bromii	Ruminococcus callidus					Intestinibacter bartlettii			Megamonas hypermegale	Megamonas rupellensis	Mitsuokella multacida		Selenomonas noxia
Oral	Alam et al. 2020 [38]			↑				↑																	
	Sharma et al. 2020 [58]																								
	Van der Meulen et al. 2018 [61]																								
	Kim et al. 2022 [47]																								
	Van der Meulen et al. 2018 [62]																								
	Zhou et al. 2018 [69]																								
	Li et al. 2016 [49]																								
	Xie et al. 2024 [67]																								
	Martinez-Nava et al. 2023 [55]																								
Gut	Jia et al. 2023 [46]			↑							↓	↓	↓					↑			↓				
	Van der Meulen et al. 2019 [63]																								
	Wu et al. 2019 [66]																								
	Moon et al. 2020 [57]																								
	Cano-Ortiz et al. 2020 [41]										↓														
	Mandl et al. 2017 [54]										↓														
	Mendez et al. 2020 [56]																								
	Yang et al. 2022 [68]										↓														
	Goodman et al. 2023 [43]	↑	↑	↑	↑	↑	↑	↑	↑																
	Wang et al. 2023 [64]							↑			↓														

	Phylum																								
	Family		Streptococcaceae													Vallitaleaceae	Veillonellaceae								
	Genus		Streptococcus													Vallitalea	Anaeroglobus	Dialister		Megasphaera	Veillonella				
Sjögren's syndrome	Species	Selenomonas ruminatum	Streptococcus anginosus	Streptococcus australis	Streptococcus constellatus	Streptococcus cristatus	Streptococcus infantis	Streptococcus intermedius	Streptococcus lutetiensis	Streptococcus mutans	Streptococcus oralis	Streptococcus parasanguinis	Streptococcus sanguinis	Streptococcus sobrinus	Streptococcus suis	Vallitalea pronyensis	Anaeroglobus geminatus	Dialister invisus	Dialister succinatiphilus	Megasphera micronuciformis	Veillonella atypica	Veillonella dispar	Veillonella parvula	Veillonella rodentium	Veillonella rogosae
Oral	Alam et al. 2020 [38]		↑					↑		↑		↑	↓	↑									↑	↑	↓
	Sharma et al. 2020 [58]																								
	Van der Meulen et al. 2018 [61]																								
	Kim et al. 2022 [47]																								
	Van der Meulen et al. 2018 [62]																								
	Zhou et al. 2018 [69]																								
	Li et al. 2016 [49]																								
	Xie et al. 2024 [67]																								
	Martinez-Nava et al. 2023 [55]																								
Gut	Jia et al. 2023 [46]		↑	↓			↓			↑		↑										↑	↑		
	Van der Meulen et al. 2019 [63]																								
	Wu et al. 2019 [66]																								
	Moon et al. 2020 [57]																								
	Cano-Ortiz et al. 2020 [41]																								
	Mandl et al. 2017 [54]																								
	Mendez et al. 2020 [56]																								
	Yang et al. 2022 [68]																								
	Goodman et al. 2023 [43]	↑	↑												↑						↑				
	Wang et al. 2023 [64]								↑	↑															

Study-level findings of relative abundance of gut and oral microbes at the species level

Sjögren's syndrome	Bacteroides caccae	Bacteroides cellulosilyticus	Bacteroides clarus	Bacteroides eggerthii	Bacteroides fragilis	Bacteroides intestinalis	Bacteroides ovatus	Bacteroides salyersiae	Bacteroides thetaiotaomicron	Bacteroides uniformis	Phocaeicola coprocola	Phocaeicola dorei	Phocaeicola plebeius	Phocaeicola vulgatus
Phylum	Bacteroidota (Bacteroidetes)													
Family	Bacteroidaceae													
Genus	Bacteroides										Phocaeicola			
**Oral**														
Alam et al. 2020 [38]														
Sharma et al. 2020 [58]														
Van der Meulen et al. 2018 [61]														
Kim et al. 2022 [47]														
Van der Meulen et al. 2018 [62]														
Zhou et al. 2018 [69]														
Li et al. 2016 [49]														
Xie et al. 2024 [67]														
Martinez-Nava et al. 2023 [55]														
**Gut**														
Jia et al. 2023 [46]					↑ (dark green)								↓ (dark red)	
Van der Meulen et al. 2019 [63]	↑ (light green)						↑ (dark green)			↑ (dark green)		↑ (light green)	↑ (light green)	↑ (dark green)
Wu et al. 2019 [66]														
Moon et al. 2020 [57]														
Cano-Ortiz et al. 2020 [41]					↓ (dark red)									
Mandl et al. 2017 [54]														
Mendez et al. 2020 [56]														
Yang et al. 2022 [68]														
Goodman et al. 2023 [43]											↑ (dark green)			
Wang et al. 2023 [64]											↓ (dark red)		↓ (dark red)	

Sjögren's syndrome	Barnesiella intestinihominis	Coprobacter fastidiosus	Capnocytophaga gingivalis	Capnocytophaga leadbetteri	Odoribacter splanchnicus	Porphyromonas gingivalis		Hoylesella enoeca	Hoylesella nanceiensis	Hoylesella pleuritidis	Hoylesella shahii	Leyella stercorea	Paraprevotella clara	Prevotella genomosp	Prevotella histicola	Prevotella melaninogenica	Prevotella micans	Prevotella nigrescens
Phylum	Bacteroidota (Bacteroidetes)																	
Family	Barnesiellaceae		Flavobacteriaceae		Odoribacteraceae	Porphyromonadaceae	Prevotellaceae											
Genus	Barnesiella	Coprobacter	Capnocytophaga		Odoribacter	Porphyromonas	Alloprevotella	Hoylesella				Leyella	Paraprevotella	Prevotella				
**Oral**																		
Alam et al. 2020 [38]			↓ (dark red)	↓ (dark red)		↓ (dark red)					↓ (dark red)				↑ (dark green)	↑ (light green)		↓ (dark red)
Sharma et al. 2020 [58]																		
Van der Meulen et al. 2018 [61]																		
Kim et al. 2022 [47]																		
Van der Meulen et al. 2018 [62]																		
Zhou et al. 2018 [69]																		
Li et al. 2016 [49]																		
Xie et al. 2024 [67]																		
Martinez-Nava et al. 2023 [55]																		
**Gut**																		
Jia et al. 2023 [46]	↓ (light red)												↓ (dark red)					
Van der Meulen et al. 2019 [63]																		
Wu et al. 2019 [66]																		
Moon et al. 2020 [57]																		
Cano-Ortiz et al. 2020 [41]																		
Mandl et al. 2017 [54]																		
Mendez et al. 2020 [56]																		
Yang et al. 2022 [68]																		
Goodman et al. 2023 [43]												↓ (dark red)						
Wang et al. 2023 [64]					↓ (dark red)													

Sjögren's syndrome	Segatella copri	Segatella oris	Segatella oulorum	Segatella salivae	Alistipes finegoldii	Alistipes onderdonkii	Alistipes putredinis	Alistipes senegalensis	Alistipes shahii	Parabacteroides distasonis	Parabacteroides merdae			Chryseobacterium taiwanens
Phylum	Bacteroidota (Bacteroidetes)													
Family	Prevotellaceae								Rikenellaceae	Tannerellaceae			Weeksellaceae	
Genus	Segatella								Alistipes	Parabacteroides		Tannerella	Bergeyella	Chryseobacterium
**Oral**														
Alam et al. 2020 [38]				↑ (dark green)										
Sharma et al. 2020 [58]														
Van der Meulen et al. 2018 [61]														
Kim et al. 2022 [47]														
Van der Meulen et al. 2018 [62]														
Zhou et al. 2018 [69]														
Li et al. 2016 [49]														
Xie et al. 2024 [67]														
Martinez-Nava et al. 2023 [55]														
**Gut**														
Jia et al. 2023 [46]	↓ (light red)				↓ (light red)	↓ (light red)	↓ (light red)	↓ (light red)			↑ (light green)			
Van der Meulen et al. 2019 [63]														
Wu et al. 2019 [66]														
Moon et al. 2020 [57]														
Cano-Ortiz et al. 2020 [41]	↑ (dark green)									↓ (dark red)				
Mandl et al. 2017 [54]														
Mendez et al. 2020 [56]														
Yang et al. 2022 [68]														
Goodman et al. 2023 [43]														
Wang et al. 2023 [64]											↓ (dark red)			

Sjögren's syndrome	Campylobacter coli	Campylobacter concisus	Campylobacter gracilis	Campylobacter showae	
Phylum	Campylobacterota				Cyanobacteriota (Cyanobacteria)
Family	Campylobacteraceae				
Genus	Campylobacter				
**Oral**					
Alam et al. 2020 [38]		↓ (dark red)		↓ (dark red)	
Sharma et al. 2020 [58]					
Van der Meulen et al. 2018 [61]					
Kim et al. 2022 [47]					
Van der Meulen et al. 2018 [62]					
Zhou et al. 2018 [69]					
Li et al. 2016 [49]					
Xie et al. 2024 [67]			↑ (light green)		
Martinez-Nava et al. 2023 [55]					
**Gut**					
Jia et al. 2023 [46]					
Van der Meulen et al. 2019 [63]					
Wu et al. 2019 [66]					
Moon et al. 2020 [57]					
Cano-Ortiz et al. 2020 [41]					
Mandl et al. 2017 [54]					
Mendez et al. 2020 [56]					
Yang et al. 2022 [68]					
Goodman et al. 2023 [43]	↑ (dark green)				
Wang et al. 2023 [64]					

Study-level findings of relative abundance of gut and oral microbes at the species level

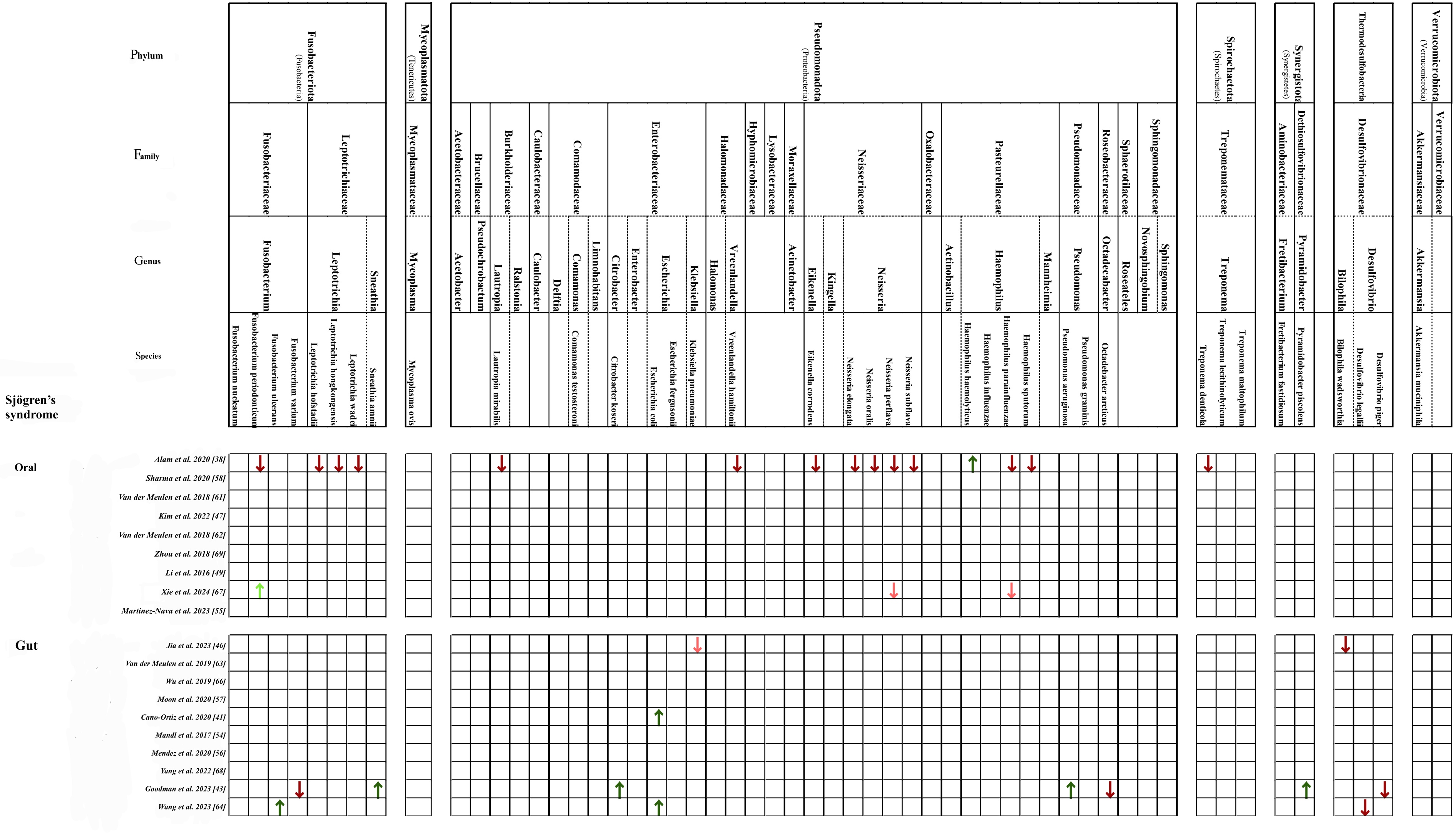

Supplement: Supplementary Data Sheet 2 — Relative abundance changes in oral and gut. [file DataSheet2.pdf]
