# Supplementary material for: Coordinated oral–gut microbiota relocation in connective tissue diseases: a systematic review
Source: Front Immunol. 2026 Jul 3;17:1841874. doi: 10.3389/fimmu.2026.1841874 (PMC13376073; doi:10.3389/fimmu.2026.1841874)
Supplement: Supplementary Data Sheet 3 — Relative abundance changes associated with PPI use. [file DataSheet3.pdf]

Study-level findings of relative abundance of gut and oral microbe with PPI intake at the phylum level

Disease	Site	phylum	Actinomycetota (Actinobacteria)	Bacillota (Firmicutes)	Bacteroidota (Bacteroidetes)	Campylobacterota	Cyanobacteriota (Cyanobacteria)	Fusobacteriota (Fusobacteria)	Mycoplasmatota (Tenericutes)	Pseudomonadota (Proteobacteria)	Spirochaetota (Spirochaetes)	Synergisto (Synergiste	Thermodesulfobacteria	Verrucomicrobiota (Verrucomicrobia)	proportion of patients on PPI
Systemic Lupus Erythematosus	gut	16. Van der Meulen et al. 2019	↓	↓	⬆					⬆				=	18/28
Sjögren's Syndrome	oral	21. Van der Meulen 2018 et al. (a)	↑	↑	=			=		⬇					16/37
		23. Kim et al. 2022													1\23
		26. van der Meulen et al. 2018 (b)													14/36
	gut	16. Van der Meulen et al. 2019	↓	↓	⬆					⬆					13/36
		24. Mandl et al. 2017													36/42

Legend	Symbol
significantly increased (p<0,05)	⬆
increased	↑
significantly decreased (p<0,05)	⬇
decreased	↓
no significant difference	=
Presumption supported by data on different levels	*
comparing patients with HC	

Study-level findings of relative abundance of gut and oral microbes with PPI intake at the family level

Legend:
- significantly increased (p<0,05) ⬆
- increased ↑
- significantly decreased (p<0,05) ⬇
- decreased ↓
- no significant difference =
- Presumption supported by data on different levels *
- Comparing patients with HC

		phylum	Actinomycetota (Actinobacteria)								
		family	Actinomycetaceae	Atopobiaceae	Bifidobacteriaceae	Coriobacteriaceae	Corynebacteriaceae	Eggerthellaceae	Microbacteriaceae	Micrococcaceae	Streptomycetaceae
Systemic Lupus Erythematosus	gut	16.. Van der Meulen et al. 2019									
Sjögren´s Syndrome	oral	21. Van der Meulen 2018 et al.(a)			⬆	⬆					
		23. Kim et al. 2022									
		26. van der Meulen et al. 2018 (b)									
	gut	16. Van der Meulen et al. 2019									
		24. Mandl et al. 2017									

		phylum	Bacillota (Firmicutes)																					
		family	Acidaminococcaceae	Aerococcaceae	Carnobacteriaceae	Catabacteraceae	Clostridiaceae	Coprobacillaceae	Defluviitaleceaea	Enterococcaceae	Erysipelotrichaceae	Eubacteriaceae	Filifactoraceae	Gemellaceae	Lachnospiraceae	Lactobacillaceae	Oscillospiraceae (Ruminococcaceae)	Paenibacillaceae	Peptoniphiliaceae	Peptostreptococcaceae	Selenomonadaceae	Streptococcaceae	Vallitaleaceae	Veillonellaceae
Systemic Lupus Erythematosus	gut	16.. Van der Meulen et al. 2019																						
Sjögren´s Syndrome	oral	21. Van der Meulen 2018 et al.(a)		⬇	⬇					⬇						⬆	⬇		⬆					* ↑
		23. Kim et al. 2022																						
		26. van der Meulen et al. 2018 (b)																						
	gut	16. Van der Meulen et al. 2019															* ↑							
		24. Mandl et al. 2017																						

		phylum	Bacteroidota (Bacteroidetes)									
		family	Bacteroidaceae	Barnesiellaceae	Flavobacteriaceae	Odoribacteraceae	Porphyromonadaceae	Prevotellaceae	Rikenellaceae	Tannerellaceae	Weeksellaceae	proportion of patients on PPI
Systemic Lupus Erythematosus	gut	16.. Van der Meulen et al. 2019										18/28
Sjögren´s Syndrome	oral	21. Van der Meulen 2018 et al.(a)										16/37
		23. Kim et al. 2022										1\23
		26. van der Meulen et al. 2018 (b)										14/36
	gut	16. Van der Meulen et al. 2019										13/36
		24. Mandl et al. 2017										36/42

		phylum	Campylobacterota	Cyanobacteriota (Cyanobacteria)	Fusobacteriota (Fusobacteria)		Mycoplasmatota (Tenericutes)
		family	Campylobacteraceae		Fusobacteriaceae	Leptotrichiaceae	Mycoplasmataceae
Systemic Lupus Erythematosus	gut	16.. Van der Meulen et al. 2019					
Sjögren´s Syndrome	oral	21. Van der Meulen 2018 et al.(a)					
		23. Kim et al. 2022					
		26. van der Meulen et al. 2018 (b)					
	gut	16. Van der Meulen et al. 2019					
		24. Mandl et al. 2017					

		phylum	Pseudomonadota (Proteobacteria)															
		family	Acetobacteraceae	Brucellaceae	Burkholderiaceae	Caulobacteraceae	Comamnodaceae	Enterobacteriaceae	Halomonadaceae	Hyphomicrobiaceae	Lysobacteraceae (Xanthomonadaceae)	Moraxellaceae	Neisseriaceae	Oxalobacteraceae	Pasteurellaceae	Roseobacteraceae	Sphaerotilaceae	Sphingomonadaceae
Systemic Lupus Erythematosus	gut	16.. Van der Meulen et al. 2019																
Sjögren´s Syndrome	oral	21. Van der Meulen 2018 et al.(a)			⬇								⬇		⬇			
		23. Kim et al. 2022																
		26. van der Meulen et al. 2018 (b)																
	gut	16. Van der Meulen et al. 2019																
		24. Mandl et al. 2017																

		phylum	Spirochaetota (Spirochaetes)	Synergistota (Synergistetes)	Thermodesulfobacteria		Verrucomicrobiota (Verrucomicrobia)		
		family	Treponemataceae	Dethiosulfovibrionaceae	Bilophilacea	Desulfovibrionaceae	Akkermansiaceae	Verrucomicrobiaceae	proportion of patients on PPI
Systemic Lupus Erythematosus	gut	16.. Van der Meulen et al. 2019							18/28
Sjögren´s Syndrome	oral	21. Van der Meulen 2018 et al.(a)							16/37
		23. Kim et al. 2022							1\23
		26. van der Meulen et al. 2018 (b)							14/36
	gut	16. Van der Meulen et al. 2019							13/36
		24. Mandl et al. 2017							36/42

Study-level findings of relative abundance of gut and oral microbes with PPI intake at the genus level

Symbols used below: ↑ (sig.) = significantly increased (p<0,05); ↑ = increased; ↓ (sig.) = significantly decreased (p<0,05); ↓ = decreased; = no significant difference; * = presumption supported by data on different levels.

		genus	Actinomyces	Mobiluncus	Schaalia	Atopobium	Lancefieldella	Olsenella	Alloscardovia	Bifidobacterium	Gardnerella	Parascardovia	Scardovia	Collinsella	Corynebacterium	Adlercreutzia	Eggerthella	Gordonibacter	Leucobacter	Rothia	
		phylum	Actinomycetota (Actinobacteria)																		
		family	Actinomycetaceae			Atopobiaceae			Bifidobacteriaceae					Coriobacteriaceae	Corynebacteriaceae	Eggerthellaceae			Microbacteriaceae	Micrococcaceae	Streptomycetaceae
Systemic Lupus Erythematosus	gut	16. Van der Meulen et al. 2019	↓ (sig.)											↓							
Sjögren´s Syndrome	oral	21. Van der Meulen 2018 (a) et al.				↑ (sig.)			↑ (sig.)	↑ (sig.)											
		23. Kim et al. 2022																			
		26. van der Meulen et al. 2018 (b)																		=	
	gut	16. Van der Meulen et al. 2019	↓ (sig.)							↓				↓							
		24. Mandl et al. 2017								↓ (sig.)											

		genus	Phascolarctobacterium	Abiotrophia	Granulicatella	Clostridium	Thomasclavelia		Enterococcus	Dielma	Eubacterium	Filifactor	Gemella
		phylum	Bacillota (Firmicutes)										
		family	Acidaminococcaceae	Aerococcaceae	Carnobacteriaceae	Clostridiaceae	Coprobacillaceae	Defluviitaleaceae	Enterococcaceae	Erysipelotrichaceae	Eubacteriaceae	Filifactoraceae	Gemellaceae
Systemic Lupus Erythematosus	gut	16. Van der Meulen et al. 2019											
Sjögren´s Syndrome	oral	21. Van der Meulen 2018 (a) et al.		↓ (sig.)	↓ (sig.)				↓ (sig.)				↑
		23. Kim et al. 2022											
		26. van der Meulen et al. 2018 (b)											
	gut	16. Van der Meulen et al. 2019											
		24. Mandl et al. 2017											

		genus	Agathobacter	Anaerobutyricum	Blautia	Coprococcus	Dorea	Enterocloster	Johnsonella	Lachnoanaerobaculum	Lachnoclostridium	Lachnospira	Mediterraneibacter	Otoolea	Oribacterium	Roseburia	Shuttleworthella	Solobacterium	Stomatobaculum	Tyzzerella
		phylum	Bacillota (Firmicutes)																	
		family	Lachnospiraceae																	
Systemic Lupus Erythematosus	gut	16. Van der Meulen et al. 2019			=		=					↑ (sig.)								
Sjögren´s Syndrome	oral	21. Van der Meulen 2018 (a) et al.																		
		23. Kim et al. 2022																		
		26. van der Meulen et al. 2018 (b)																		
	gut	16. Van der Meulen et al. 2019			=		=					↑ (sig.)								
		24. Mandl et al. 2017																		

		genus	Lactobacillus	Latilactobacillus	Ligilactobacillus	Limosilactobacillus	Weissenella	Faecalibacterium	Ruminococcus	Anaerococcus	Finegoldia	Parvimonas	Peptophilus	Peptostreptococcus	Romboutsia	Megamonas	Mitsuokella	Schwartzia	Selenomonas	Streptococcus	Vallitalea	Allisonella	Anaeroglobus	Dialister	Megasphaera	Veillonella	proportion of patients on PPI
		phylum	Bacillota (Firmicutes)																								
		family	Lactobacillaceae					Oscillospiraceae (Ruminococcaceae)		Peptoniphilaceae				Peptostreptococcaceae		Selenomonadaceae				Streptococcaceae	Vallitaleaceae	Veillonellaceae					
Systemic Lupus Erythematosus	gut	16. Van der Meulen et al. 2019													↓ (sig.)					↑				↓			18/28
Sjögren´s Syndrome	oral	21. Van der Meulen 2018 (a) et al.	↑ (sig.)						↓ (sig.)			↑ (sig.)		↑ (sig.)						↓		↑ (sig.)		↑ (sig.)		↑	16/37
		23. Kim et al. 2022																							=		1\23
		26. van der Meulen et al. 2018 (b)				↑													↑ (sig.)	↓ (sig.)						↑	14/36
	gut	16. Van der Meulen et al. 2019						↑	↑						↓ (sig.)					↑				↓			13/36
		24. Mandl et al. 2017																									36/42

Legend:

- significantly increased (p<0,05) ↑
- increased ↑
- significantly decreased (p<0,05) ↓
- decreased ↓
- no significant difference =
- Presumption supported by data on different levels *
- Comparing patients with HC

		genus	Bacteroides	Phocaeicola	Barnesiella	Coprobacter	Capnocytophaga	Odoribacter	Porphyromonas	Alloprevotella	Hoylesella	Leyella	Paraprevotella	Prevotella	Segatella	Alistipes	Parabacteroides	Tannerella	Bergeyella	Chryseobacterium	Campylobacter		Fusobacterium	Leptotrichia	Sneathia	Mycoplasma
		phylum	Bacteroidota (Bacteroidetes)																		Campylobacterota	Cyanobacteriota (Cyanobacteria)	Fusobacteriota (Fusobacteria)			Mycoplasma (Teneri
		family	Bacteroidaceae		Barnesiellaceae		Flavobacteriaceae	Odoribacteraceae	Porphyromonadaceae	Prevotellaceae						Rikenellaceae	Tannerellaceae		Weeksellaceae		Campylobacteraceae		Fusobacteriaceae	Leptotrichiaceae		Mycoplasmataceae
Systemic Lupus Erythematosus	gut	16. Van der Meulen et al. 2019	↑ (sig.)	* ↑	↑ (sig.)											↑ (sig.)								↑ (sig.)		
Sjögren´s Syndrome	oral	21. Van der Meulen 2018 (a) et al.								↓ (sig.)										↓ (sig.)			=	=		
		23. Kim et al. 2022																								
		26. van der Meulen et al. 2018 (b)																					↑			
	gut	16. Van der Meulen et al. 2019	↑ (sig.)	* ↑	↑ (sig.)											↑ (sig.)										
		24. Mandl et al. 2017														↓ (sig.)										

		genus	Acetobacter	Pseudochrobactrum	Lautropia	Ralstonia	Caulobacter	Delftia	Comamonas	Limnohabitans	Citrobacter	Enterobacter	Escherichia	Klebsiella	Halomonas	Vreenlandella			Acinetobacter	Eikenella	Kingella	Neisseria		Actinobacillus	Haemophilus	Mannheimia	Pseudomonas	Octadecabacter	Roseateles (Mitsuaria)	Novosphingobium	Sphingomonas
		phylum	Pseudomonadota (Proteobacteria)																												
		family	Acetobacteraceae	Brucellaceae	Burkholderiaceae		Caulobacteraceae	Comamonadaceae			Enterobacteriaceae				Halomonadaceae		Hyphomicrobiaceae	Lysobacteraceae	Moraxellaceae	Neisseriaceae			Oxalobacteraceae	Pasteurellaceae			Pseudomonadaceae	Roseobacteraceae	Sphaerotilaceae	Sphingomonadaceae	
Systemic Lupus Erythematosus	gut	16. Van der Meulen et al. 2019																													
Sjögren´s Syndrome	oral	21. Van der Meulen 2018 (a) et al.			↓ (sig.)																	↓ (sig.)			↓ (sig.)						
		23. Kim et al. 2022																													
		26. van der Meulen et al. 2018 (b)																							↓						
	gut	16. Van der Meulen et al. 2019																													
		24. Mandl et al. 2017																													

		genus	Treponema	Fretibacterium	Pyramidobacter	Bilophila	Desulfovibrio	Akkermansia		proportion of patients on PPI
		phylum	Spirochaetota (Spirochaetes)	Synergistota (Synergistetes)		Thermodesulfobacteria		Verrucomicrobiota (Verrucomicrobia)		
		family	Treponemataceae	Aminobacteriaceae	Dethiosulfovibrionaceae	Desulfovibrionaceae		Akkermansiaceae	Verrucomicrobiaceae	
Systemic Lupus Erythematosus	gut	16. Van der Meulen et al. 2019								18/28
Sjögren´s Syndrome	oral	21. Van der Meulen 2018 (a) et al.								16/37
		23. Kim et al. 2022								1\23
		26. van der Meulen et al. 2018 (b)								14/36
	gut	16. Van der Meulen et al. 2019								13/36
		24. Mandl et al. 2017								36/42

Study-level findings of relative abundance of gut and oral with PPI intake microbes at the species level

Rows of the original table (study rows):
- Systemic Lupus Erythematosus, gut: 16. Van der Meulen et al. 2019 (proportion of patients on PPI 18/28)
- Sjögren´s Syndrome, oral: 21. Van der Meulen et al. 2018 (a) (16/37); 23. Kim et al. 2022 (1\23); 26. van der Meulen et al. 2018 (b) (14/36)
- Sjögren´s Syndrome, gut: 16.. Van der Meulen et al. 2019 (13/36); 24. Mandl et al. 2017 (36/42)

phylum	family	genus	species	SLE gut: 16. Van der Meulen et al. 2019 (18/28)	SS oral: 21. Van der Meulen 2018 (a) et al. (16/37)	SS oral: 23. Kim et al. 2022 (1\23)	SS oral: 26. van der Meulen et al. 2018 (b) (14/36)	SS gut: 16.. Van der Meulen et al. 2019 (13/36)	SS gut: 24. Mandl et al. 2017 (36/42)
Bacillota (Firmicutes)	Acidaminococcaceae	Phascolarctobacterium							
	Aerococcaceae	Abiotrophia	Abiotrophia defectiva						
	Carnobacteriaceae	Granulicatella	Granulicatella elegans						
	Coprobacillaceae	Thomoclavelia	Thomoclavelia ramosa						
	Clostridiaceae	Clostridium	Clostridium perfringens						
	Defluviitaleceaea								
	Enterococcaceae	Enterococcus	Enterococcus avium						
			Enterococcus casseliflavus						
	Erysipelotrichaceae	Dielma	Dielma fastidiosa						
		Holdemanella	Holdemanella biformis						
	Eubacteriaceae	Eubacterium	Eubacterium limosum						
			Eubacterium ruminatum						
			Eubacterium ventriosum						
	Filifactoraceae	Filifactor	Filifactor alocis						
	Gemellaceae	Gemella	Gemella morbillorum						
	Lachnospiraceae	Agathobacter	Agathobacter rectalis						
		Anaerobutyricum	Anaerobutyricum hallii						
		Blautia	Blautia faecis						
			Blautia obeum						
			Blautia wexlerae						
		Coprococcus	Coprococcus catus						
			Coprococcus comes						
			Coprococcus eutactus						
		Dorea	Dorea longicatena						
		Enterocloster	Enterocloster aldenensis						
			Enterocloster boltae						
			Enterocloster clostridiformis						
		Johnsonella	Johnsonella ignava						
		Lachnoanaerobaculum -	Lachnoanaerobaculum orale						
		Lachnoclostridium	Clostridium scindens						
		Lachnospira	Lachnospira eligens						
		Mediterraneibacter	Mediterraneibacter gnavus						
			Ruminococcus lactaris						
			Ruminococcus torques						
		Otoolea	Clostridium symbiosum						
		Oribacterium	Oribacterium asaccharolyticum						
		Roseburia	Roseburia hominis						
			Roseburia intestinalis						
			Roseburia inulinivorans						
		Shuttleworthella	Shuttleworthella satelles						
		Solobacterium							
		Stomatobaculum	Stomatobaculum longum						
		Tyzzerella	Clostridium nexile						
	Lactobacillaceae	Lactobacillus	Lactobacillus crispatus						
			Lactobacillus delbrueckii						
			Lactobacillus gasseri						
			Lactobacillus iners						
			Lactobacillus johnsonii						
		Latilactobacillus	Latilactobacillus sakei						
		Ligilactobacillus	Ligilactobacillus ruminis						
			Ligilactobacillus salivarius						
		Limosilactobacillus	Limosilactobacillus fermentum						
			Limosilactobacillus mucosae						
			Limosilactobacillus oris						
			Limosilactobacillus reuteri						
			Limosilactobacillus vaginalis						
		Weissenella							
	Oscillospiraceae (Ruminococcaceae)	Faecalibacterium	Faecalibacterium prausnizii						↓ (decreased)
		Ruminococcus	Ruminococcus bromii						
			Ruminococcus callidus						
	Peptoniphiliaceae	Anaerococcus							
		Finegoldia							
		Peptophilus							
		Parvimonas							
	Peptostreptococcaceae	Intestinibacter	Intestinibacter bartlettii						
		Peptostreptococcus							
		Romboutsia							
	Selenomonadaceae	Megamonas	Megamonas hypermegale						
			Megamonas rupellensis						
		Mitsuokella -	Mitsuokella multacida						
		Schwartzia							
		Selenomonas	Selenomonas noxia						
			Selenomonas ruminatum						
	Streptococcaceae	Streptococcus	Streptococcus anginosus						
			Streptococcus australis						
			Streptococcus constellatus						
			Streptococcus cristatus						
			Streptococcus infantis						
			Streptococcus intermedius						
			Streptococcus lutetiensis						
			Streptococcus mutans						
			Streptococcus oralis						
			Streptococcus parasanguinis						
			Streptococcus sanguinis						
			Streptococcus sobrinus						
			Streptococcus suis						
	Vallitaleaceae	Vallitalea	Vallitalea pronyensis						
	Veillonellaceae	Anaeroglobus	Anaeroglobus geminatus						
		Dialister	Dialister invisus						
			Dialister succinatiphilus						
		Megasphaera	Megasphera micronuciformis						
		Veillonella	Veillonella atypica						
			Veillonella dispar						
			Veillonella parvula						
			Veillonella rodentium						
			Veillonella rogosae						

Legend:
- significantly increased (p<0,05) ↑ (dark green)
- increased ↑ (light green)
- significantly decreased (p<0,05) ↓ (dark red)
- decreased ↓ (light red)
- no significant difference =
- Presumption supported by data on different levels *
- Comparing patients with HC

phylum	family	genus	species	SLE gut: 16. Van der Meulen et al. 2019 (18/28)	SS oral: 21. Van der Meulen 2018 (a) et al. (16/37)	SS oral: 23. Kim et al. 2022 (1\23)	SS oral: 26. van der Meulen et al. 2018 (b) (14/36)	SS gut: 16.. Van der Meulen et al. 2019 (13/36)	SS gut: 24. Mandl et al. 2017 (36/42)
Bacteroidota (Bacteroidetes)	Bacteroidaceae	Bacteroides	Bacteroides caccae					↑ (increased)	
			Bacteroides cellulosilyticus						
			Bacteroides clarus						
			Bacteroides eggerthii						
			Bacteroides fragilis						
			Bacteroides intestinalis						
			Bacteroides ovatus	↑ (significantly increased)				↑ (significantly increased)	
			Bacteroides salyersiae						
			Bacteroides thetaiotaomicron	↑ (significantly increased)					
			Bacteroides uniformis	↑ (significantly increased)				↑ (significantly increased)	
		Phocaeicola	Phocaeicola coprocola						
			Phocaeicola dorei	↑ (increased)				↑ (increased)	
			Phocaeicola plebeius	↑ (increased)				↑ (increased)	
			Phocaeicola vulgatus	↑ (significantly increased)				↑ (significantly increased)	
	Barnesiellaceae	Barnesiella	Barnesiella intestinihominis						
		Coprobacter	Coprobacter fastidiosus						
	Flavobacteriaceae	Capnocytophaga	Capnocytophaga gingivalis						
			Capnocytophaga leadbetteri						
	Odoribacteraceae	Odoribacter	Odoribacter splanchnicus						
	Porphyromonadaceae	Porphyromonas	Porphyromonas gingivalis						
	Prevotellaceae	Alloprevotella							
		Hoylesella	Hoylesella enoeca						
			Hoylesella nanceiensis						
			Hoylesella pleuritidis						
			Hoylesella shahii						
		Leyella	Leyella stercorea						
		Paraprevotella	Paraprevotella clara						
		Prevotella	Prevotella genomosp						
			Prevotella histicola						
			Prevotella melaninogenica						
			Prevotella micans						
			Prevotella nigrescens						
		Segatella	Segatella copri						
			Segatella oris						
			Segatella oulorum						
			Segatella salivae						
			Alistipes finegoldii						
			Alistipes onderdonkii						
			Alistipes putredinis						
			Alistipes senegalensis						
	Rikenellaceae	Alistipes	Alistipes shahii						
	Tannerellaceae	Parabacteroides	Parabacteroides distasonis						
			Parabacteroides merdae						
		Tannerella							
	Weeksellaceae	Bergeyella							
		Chryseobacterium	Chryseobacterium taiwanense						

-> only phyla with data on species level shown
