# Supplementary material for: Coordinated oral–gut microbiota relocation in connective tissue diseases: a systematic review
Source: Front Immunol. 2026 Jul 3;17:1841874. doi: 10.3389/fimmu.2026.1841874 (PMC13376073; doi:10.3389/fimmu.2026.1841874)
Supplement: Supplementary Data Sheet 4 — Relative abundance changes associated with corticosteroid use. [file DataSheet4.pdf]

Study-level findings of relative abundance of microbes at the phylum level based on corticosteroid intake of patients

Legend:

Symbol	Meaning
↑	Significantly increased (p<0,05)
(↑)	Increased (trend)
↓	Significantly decreased (p<0,05)
(↓)	Decreased (trend)
=	No significant difference
*	Presumption supported by data on different levels
	Comparing patients with HC

Systemic lupus erythematosus

Oral

Phylum	Actinomycetota (Actinobacteria)	Bacillota (Firmicutes)	Bacteroidota (Bacteroidetes)	Campylobacterota	Cyanobacteriota (Cyanobacteria)	Fusobacteriota (Fusobacteria)	Mycoplasmatota (Tenericutes)	Pseudomonadota (Proteobacteria)	Spirochaetota (Spirochaetes)	Synergistota (Synergistetes)	Thermodesulfobacteria	Verrucomicrobiota (Verrucomicrobia)	Proportion of patients	Dose
**Patients on corticosteroids**														
Liu et al. 2021 [53]	↓	(↓)	(↑)			(↑)		(↑)					30/31 pred.	7,5 mg/d
Correa et al 2017 [42]													N/A	N/A
Li et al. 2020 [48]	↓				↑		↓	*(↓)					N/A	N/A
**Patients not on corticosteroids**													**Absence prior study**	
Guo et al. 2023 [44]	↓	↑	↑	↑	↑	↑		↓					N/A	

Gut

Phylum	Actinomycetota	Bacillota	Bacteroidota	Campylobacterota	Cyanobacteriota	Fusobacteriota	Mycoplasmatota	Pseudomonadota	Spirochaetota	Synergistota	Thermodesulfobacteria	Verrucomicrobiota	Proportion of patients	Dose
**Patients on corticosteroids**														
Liu et al. 2021 [53]	(↑)	(↓)	(↓)				(↑)	(↓)					30/31 pred.	7,5 mg/d
Li et al. 2019 [50]	=	=	=			↑	↓	=		=		=	33/40 glucocort.	N/A
Ling et al. 2023 [52]	(↑)	(↑)	↓			(↑)		(↓)					26/30 pred.	avg. 24,4 mg/d
Azzouz et al. 2023 [40]													8/16 pred.	avg. 23,75 mg/d
Azzouz et al. 2019 [39]	=	=	=			=		(↓)				(↑)	22/61 pred + 1/26 methylpred.	avg. 11,15 mg/d + 20mg/c
Toumi et al. 2022 [60]							↓						4/16 pred.	N/A
Van der Meulen et al. 2019 [63]	(↓)	(↓)	↑					↑				=	5/28 corticost.	max. 7,5 mg/d
**Patients not on corticosteroids**													**Absence prior study**	
Hevia et al. 2014 [45]	=	↓	↑		=	=		(↓)				=	6 months	
Jia et al. 2023 [46]													3 months	
Wei et al. 2019 [65]		*(↓)						↑					1 month	
Chen et al. 2021 [18]													N/A	

Sjögren's syndrome

Oral

Phylum	Actinomycetota (Actinobacteria)	Bacillota (Firmicutes)	Bacteroidota (Bacteroidetes)	Campylobacterota	Cyanobacteriota (Cyanobacteria)	Fusobacteriota (Fusobacteria)	Mycoplasmatota (Tenericutes)	Pseudomonadota (Proteobacteria)	Spirochaetota (Spirochaetes)	Synergistota (Synergistetes)	Thermodesulfobacteria	Verrucomicrobiota (Verrucomicrobia)	Proportion of patients	Dose
**Patients on corticosteroids**														
Li et al. 2016 [49]	(↓)	(↑)			=			↓	(↓)				4/10 pred.	10-20 mg/d
Sharma et al. 2020 [58]													11/37 ster.	N/A
Kim et al. 2022 [47]													4/23 corticost.	N/A
Van der Meulen et al. 2018 [62]													4/23 corticost.	N/A
Van der Meulen et al. 2018 [61]	(↑)	(↑)	=			=		↓					5/37 corticost.	N/A
Martinez-Nava et al. 2023 [55]	=		=	(↑)				(↓)	(↓)	(↓)			5/45 pred.	N/A
**Patients not on corticosteroids**													**Absence prior study**	
Alam et al. 2020 [38]	=	↑	(↑)			(↓)		↓	↓				1 month	
Zhou et al. 2018 [69]	=	↑	↑			=		↓					N/A	

Gut

Phylum	Actinomycetota	Bacillota	Bacteroidota	Campylobacterota	Cyanobacteriota	Fusobacteriota	Mycoplasmatota	Pseudomonadota	Spirochaetota	Synergistota	Thermodesulfobacteria	Verrucomicrobiota	Proportion of patients	Dose
**Patients on corticosteroids**														
Mandl et al. 2017 [54]													36/42 glucocort.	avg. 5mg/d
Van der Meulen et al. 2019 [63]	(↓)	(↓)	↑					↑					2/36 corticost.	N/A
**Patients not on corticosteroids**													**Absence prior study**	
Jia et al. 2023 [46]	(↓)	(↑)						(↑)				=	3 months	
Cano-Ortiz et al. 2020 [41]	↓	↓	↑				=	↑					3 months	
Wu et al. 2019 [66]	↑	↑	↓		=	↑	↓	↑					2 months	
Yang et al. 2022 [68]	(↑)	(↓)	(↓)			(↑)		(↑)				(↑)	N/A	

Study-level findings of relative abundance of microbes at the family level based on **corticosteroid intake of patients**

Systemic lupus erythematosus

Phylum	Actinomycetota (Actinobacteria)								
Family	Actinomycetaceae	Atopobiaceae	Bifidobacteriaceae	Coriobacteriaceae	Corynebacteriaceae	Eggerthellaceae	Microbacteriaceae	Micrococcaceae	Streptomycetaceae
**Oral** – Patients on corticosteroids									
Liu et al. 2021 [53]									
Correa et al 2017 [42]									
Li et al. 2020 [48]					↓			↓	
**Oral** – Patients not on corticosteroids									
Guo et al. 2023 [44]	↓							↓	
**Gut** – Patients on corticosteroids									
Liu et al. 2021 [53]			↑	↑ (light)					
Li et al. 2019 [50]									
Ling et al. 2023 [52]									
Azzouz et al. 2023 [40]									
Azzouz et al. 2019 [39]			=						
Toumi et al. 2022 [60]									
Van der Meulen et al. 2019 [63]									
**Gut** – Patients not on corticosteroids									
Jia et al. 2023 [46]			=						
Cano-Ortiz et al. 2020 [41]									
Wu et al. 2019 [66]									
Yang et al. 2022 [68]									

Phylum	Bacillota (Firmicutes)																					
Family	Acidaminococcaceae	Aerococcaceae	Carnobacteriaceae	Catabacteraceae	Clostridiaceae	Coprobacillaceae	Defluviitaleaceae	Enterococcaceae	Erysipelotrichaceae	Eubacteriaceae	Filifactoraceae	Gemellaceae	Lachnospiraceae	Lactobacillaceae	Oscillospiraceae (Ruminococcaceae)	Paenibacillaceae	Peptoniphilaceae	Peptostreptococcaceae	Selenomonadaceae	Streptococcaceae	Vallitaleaceae	Veillonellaceae
**Oral** – Patients on corticosteroids																						
Liu et al. 2021 [53]												↓										
Correa et al 2017 [42]																						
Li et al. 2020 [48]							↓ (light)							↑				↓ (light)				↑
**Oral** – Patients not on corticosteroids																						
Guo et al. 2023 [44]													↑					↓		↓ (light)		↑
**Gut** – Patients on corticosteroids																						
Liu et al. 2021 [53]													↓		↓							
Li et al. 2019 [50]														↑						↑		
Ling et al. 2023 [52]																	* ↑ (light)					
Azzouz et al. 2023 [40]																						
Azzouz et al. 2019 [39]					=				↑ (light)				↑ (light)		↓					↑ (light)		↑
Toumi et al. 2022 [60]																						
Van der Meulen et al. 2019 [63]																						
**Gut** – Patients not on corticosteroids																						
Jia et al. 2023 [46]				↓ (light)					=				↓		↓							↑ (light)
Cano-Ortiz et al. 2020 [41]																						
Wu et al. 2019 [66]					↓										↓							
Yang et al. 2022 [68]																						

Phylum	Bacteroidota (Bacteroidetes)									Campylobacterota	Cyanobacteriota (Cyanobacteria)	Fusobacteriota (Fusobacteria)		Mycoplasmatota (Tenericutes)
Family	Bacteroidaceae	Barnesiellaceae	Flavobacteriaceae	Odoribacteraceae	Porphyromonadaceae	Prevotellaceae	Rikenellaceae	Tannerellaceae	Weeksellaceae	Campylobacteraceae		Fusobacteriaceae	Leptotrichiaceae	Mycoplasmataceae
**Oral** – Patients on corticosteroids														
Liu et al. 2021 [53]														
Correa et al 2017 [42]														
Li et al. 2020 [48]														
**Oral** – Patients not on corticosteroids														
Guo et al. 2023 [44]					↓	↑				↑			↑	
**Gut** – Patients on corticosteroids														
Liu et al. 2021 [53]														
Li et al. 2019 [50]														
Ling et al. 2023 [52]														
Azzouz et al. 2023 [40]														
Azzouz et al. 2019 [39]	=				=	↑ (light)	↓ (light)							
Toumi et al. 2022 [60]								↑						
Van der Meulen et al. 2019 [63]														
**Gut** – Patients not on corticosteroids														
Jia et al. 2023 [46]					=	↑ (light)	↑ (light)							
Cano-Ortiz et al. 2020 [41]														
Wu et al. 2019 [66]						↓								
Yang et al. 2022 [68]														

Phylum	Pseudomonadota (Proteobacteria)															
Family	Acetobacteraceae	Brucellaceae	Burkholderiaceae	Caulobacteraceae	Comamonadaceae	Enterobacteriaceae	Halomonadaceae	Hyphomicrobiaceae	Lysobacteraceae (Xanthomonadaceae)	Moraxellaceae	Neisseriaceae	Oxalobacteraceae	Pasteurellaceae	Roseobacteraceae	Sphaerotilaceae	Sphingomonadaceae
**Oral** – Patients on corticosteroids																
Liu et al. 2021 [53]																
Correa et al 2017 [42]																
Li et al. 2020 [48]							↓	↓ (light)	↓	↑	* ↓ (light)					* ↓ (light)
**Oral** – Patients not on corticosteroids																
Guo et al. 2023 [44]						↑	↓				=		↓			↑
**Gut** – Patients on corticosteroids																
Liu et al. 2021 [53]						↓ (light)										
Li et al. 2019 [50]																
Ling et al. 2023 [52]																
Azzouz et al. 2023 [40]																
Azzouz et al. 2019 [39]																
Toumi et al. 2022 [60]																
Van der Meulen et al. 2019 [63]																
**Gut** – Patients not on corticosteroids																
Jia et al. 2023 [46]																
Cano-Ortiz et al. 2020 [41]																
Wu et al. 2019 [66]						↑										
Yang et al. 2022 [68]																

Phylum	Spirochaetota (Spirochaetes)	Synergistota (Synergistetes)	Thermodesulfobacteria		Verrucomicrobiota (Verrucomicrobia)			
Family	Treponemataceae	Dethiosulfovibrionaceae	Bilophiliacea	Desulfovibrionaceae	Akkermansiaceae	Verrucomicrobiaceae		
**Oral** – Patients on corticosteroids							**proportion of patients**	**dose**
Liu et al. 2021 [53]							30/31 pred.	7,5 mg/d
Correa et al 2017 [42]							N/A	N/A
Li et al. 2020 [48]							N/A	N/A
**Oral** – Patients not on corticosteroids							**abscence prior study**	
Guo et al. 2023 [44]							N/A	
**Gut** – Patients on corticosteroids							**proportion of patients**	**dose**
Liu et al. 2021 [53]							30/31 pred.	7,5 mg/d
Li et al. 2019 [50]							33/40 glucocort.	N/A
Ling et al. 2023 [52]							26/30 pred.	avg. 24,4 mg/d
Azzouz et al. 2023 [40]							8/16 pred.	avg. 23,75 mg/d
Azzouz et al. 2019 [39]						↑ (light)	22/61 pred + 1/26 methylpred.	avg. 11,15 mg/d + 20mg
Toumi et al. 2022 [60]							4/16 pred.	N/A
Van der Meulen et al. 2019 [63]							5/28 corticost.	max. 7,5 mg/d
**Gut** – Patients not on corticosteroids							**abscence prior study**	
Jia et al. 2023 [46]				↓			6 months	
Cano-Ortiz et al. 2020 [41]							3 months	
Wu et al. 2019 [66]							1 month	
Yang et al. 2022 [68]							N/A	

Study-level findings of relative abundance of microbes at the family level based on corticosteroid intake of patients

Phylum	Actinomycetota (Actinobacteria)								
Family / Sjögren's syndrome	Actinomycetaceae	Atopobiaceae	Bifidobacteriaceae	Coriobacteriaceae	Corynebacteriaceae	Eggerthellaceae	Microbacteriaceae	Micrococcaceae	Streptomyceatceae
**Oral** – Patients on corticosteroids									
Li et al. 2016 [49]									
Sharma et al. 2020 [58]		* ↑	* ↑						
Kim et al. 2022 [47]									
Van der Meulen et al. 2018 [62]									
Van der Meulen et al. 2018 [61]			↑	↑					
Martinez-Nava et al. 2023 [55]									
Patients not on corticosteroids									
Alam et al. 2020 [38]									
Zhou et al. 2018 [69]				↑					
Xie et al. 2024 [67]	↑								
**Gut** – Patients on corticosteroids									
Mandl et al. 2017 [54]									
Van der Meulen et al. 2019 [63]									
Patients not on corticosteroids									
Jia et al. 2023 [46]									
Cano-Ortiz et al. 2020 [41]			↓						
Wu et al. 2019 [66]									
Yang et al. 2022 [68]									

Phylum	Bacillota (Firmicutes)																					
Family / Sjögren's syndrome	Acidaminococcaceae	Aerococcaceae	Carnobacteriaceae	Catabacteraceae	Clostridiaceae	Coprobacillaceae	Defluviitaleacea	Enterococcaceae	Erysipelotrichaceae	Eubacteriaceae	Filifactoraceae	Gemellaceae	Lachnospiraceae	Lactobacillaceae	Oscillospiraceae (Ruminococcaceae)	Paenibacillaceae	Peptoniphiliaceae	Peptostreptococcaceae	Selenomonadaceae	Streptococcaceae	Vallitaleaceae	Veillonellaceae
**Oral** – Patients on corticosteroids																						
Li et al. 2016 [49]																						
Sharma et al. 2020 [58]														* ↑					* ↑			
Kim et al. 2022 [47]																						
Van der Meulen et al. 2018 [62]																						
Van der Meulen et al. 2018 [61]		↓	↓					↓						↑	↓		↑					* ↑
Martinez-Nava et al. 2023 [55]																						
Patients not on corticosteroids																						
Alam et al. 2020 [38]																						
Zhou et al. 2018 [69]																						
Xie et al. 2024 [67]																			↑	↓		↑
**Gut** – Patients on corticosteroids																						
Mandl et al. 2017 [54]																						
Van der Meulen et al. 2019 [63]															* ↑							
Patients not on corticosteroids																						
Jia et al. 2023 [46]					↑														↓			
Cano-Ortiz et al. 2020 [41]					↑								↓		↓			=				↑
Wu et al. 2019 [66]													↓		↑							↑
Yang et al. 2022 [68]													↓		↓							

Phylum	Bacteroidota (Bacteroidetes)									Campylobacterota	Cyanobacteriota (Cyanobacteria)	Fusobacteriota (Fusobacteria)		Mycoplasmatota (Tenericutes)
Family / Sjögren's syndrome	Bacteroidaceae	Barnesiellaceae	Flavobacteriaceae	Odoribacteraceae	Porphyromonadaceae	Prevotellaceae	Rikenellaceae	Tannerellaceae	Weeksellaceae	Campylobacteraceae		Fusobacteriaceae	Leptotrichiaceae	Mycoplasmataceae
**Oral** – Patients on corticosteroids														
Li et al. 2016 [49]														
Sharma et al. 2020 [58]													↓	
Kim et al. 2022 [47]														
Van der Meulen et al. 2018 [62]														
Van der Meulen et al. 2018 [61]														
Martinez-Nava et al. 2023 [55]													↑	
Patients not on corticosteroids														
Alam et al. 2020 [38]														
Zhou et al. 2018 [69]														
Xie et al. 2024 [67]						↑				↑		↑	↑	
**Gut** – Patients on corticosteroids														
Mandl et al. 2017 [54]														
Van der Meulen et al. 2019 [63]														
Patients not on corticosteroids														
Jia et al. 2023 [46]														
Cano-Ortiz et al. 2020 [41]	↓			↑	↓	↑	↑							
Wu et al. 2019 [66]	↑													
Yang et al. 2022 [68]	↑					↓								

Phylum	Pseudomonadota (Proteobacteria)																Spirochaetota (Spirochaetes)	Synergistota (Synergistetes)	Thermodesulfobacteria		Verrucomicrobiota (Verrucomicrobia)			
Family / Sjögren's syndrome	Acetobacteraceae	Brucellaceae	Burkholderiaceae	Caulobacteraceae	Comamodaceae	Enterobacteriaceae	Halomonadaceae	Hyphomicrobiaceae	Lysobacteraceae (Xanthomonadaceae)	Moraxellaceae	Neisseriaceae	Oxalobacteraceae	Pasteurellaceae	Roseobacteraceae	Sphaerotilaceae	Sphingomonadaceae	Treponemataceae	Dethiosulfovibrionaceae	Bilophiliacea	Desulfovibrionaceae	Akkermansiaceae	Verrucomicrobiaceae		
**Oral** – Patients on corticosteroids																							**proportion of patients**	**dose**
Li et al. 2016 [49]																							4/10 pred.	10-20 mg/d
Sharma et al. 2020 [58]													* ↓										11/37 ster.	N/A
Kim et al. 2022 [47]																							4/23 corticost.	N/A
Van der Meulen et al. 2018 [62]																							4/23 corticost.	N/A
Van der Meulen et al. 2018 [61]			↓								↓		↓										5/37 corticost.	N/A
Martinez-Nava et al. 2023 [55]																							5/45 pred.	N/A
Patients not on corticosteroids																							**abscence prior study**	
Alam et al. 2020 [38]																							1 month	
Zhou et al. 2018 [69]			↓								↓												N/A	
Xie et al. 2024 [67]	↓		↓								↓		↓										N/A	
**Gut** – Patients on corticosteroids																							**proportion of patients**	**dose**
Mandl et al. 2017 [54]																							36/42 glucocort.	avg. 5mg/d
Van der Meulen et al. 2019 [63]																							2/36 corticost.	N/A
Patients not on corticosteroids																							**abscence prior study**	
Jia et al. 2023 [46]																							3 months	
Cano-Ortiz et al. 2020 [41]						↑																	3 months	
Wu et al. 2019 [66]						↑																	2 months	
Yang et al. 2022 [68]						↑																	N/A	

Study-level findings of relative abundance of microbes at the genus level based on corticosteroid intake of patients

Systemic lupus erythematosus

Phylum: Actinomycetota (Actinobacteria)

Study	Actinomyces	Mobiluncus	Schaalia	Atopobium	Lancefieldella	Olsenella	Alloscardovia	Bifidobacterium	Gardnerella	Parascardovia	Scardovia	Collinsella	Corynebacterium	Adlercreutzia	Eggerthella	Gordonibacter	Leucobacter	Rothia	
Family	Actinomycetaceae			Atopobiaceae			Bifidobacteriaceae					Coriobacteriaceae	Corynebacteriaceae	Eggerthellaceae			Microbacteriaceae	Micrococcaceae	Streptomycetaceae
**Oral – Patients on corticosteroids**																			
Liu et al. 2021 [53]	↓																	↓	
Correa et al 2017 [42]																		↓	
Li et al. 2020 [48]		↓											↓					↓	
**Oral – Patients not on corticosteroids**																			
Guo et al. 2023 [44]																		↓	
**Gut – Patients on corticosteroids**																			
Liu et al. 2021 [53]								↓											
Li et al. 2019 [50]								↓											
Ling et al. 2023 [52]				↑					↑										
Azzouz et al. 2023 [40]																			
Azzouz et al. 2019 [39]								=											
Toumi et al. 2022 [60]																			
Van der Meulen et al. 2019 [63]	↓											↓							
**Gut – Patients not on corticosteroids**																			
Hevia et al. 2014 [45]																			
Jia et al. 2023 [46]																			
Wei et al. 2019 [65]																			
Chen et al. 2021 [18]	*↑				*↑	*↑		*↑			*↑		*↑					*↑	

Phylum: Bacillota (Firmicutes)

Study	Phascolarctobacterium	Abiotrophia	Granulicatella	Clostridium	Thomasclavelia		Enterococcus	Dielma	Eubacterium	Filifactor	Gemella	Agathobacter	Anaerobutyricum	Blautia	Coprococcus	Dorea	Enterocloster	Johnsonella	Lachnoanaerobaculum	Lachnoclostridium	Lachnospira	Mediterraneibacter	Otoolea	Oribacterium	Roseburia	Shuttleworthella	Solobacterium	Stomatobaculum	Tyzzerella
Family	Acidaminococcaceae	Aerococcaceae	Carnobacteriaceae	Clostridiaceae	Coprobacillaceae	Defluviitaleaca	Enterococcaceae	Erysipelotrichaceae	Eubacteriaceae	Filifactoraceae	Gemellaceae	Lachnospiraceae																	
**Oral – Patients on corticosteroids**																													
Liu et al. 2021 [53]			↓																										
Correa et al 2017 [42]																					↑								
Li et al. 2020 [48]										↓				↑										↓					
**Oral – Patients not on corticosteroids**																													
Guo et al. 2023 [44]			↓																↑							↓			
**Gut – Patients on corticosteroids**																													
Liu et al. 2021 [53]	↓													↑															
Li et al. 2019 [50]														↑											↓				
Ling et al. 2023 [52]	↓													↑															
Azzouz et al. 2023 [40]																													
Azzouz et al. 2019 [39]														↑		=													
Toumi et al. 2022 [60]																													
Van der Meulen et al. 2019 [63]														=		=					↑								
**Gut – Patients not on corticosteroids**																													
Hevia et al. 2014 [45]																													
Jia et al. 2023 [46]																						*↑							
Wei et al. 2019 [65]																									↓				
Chen et al. 2021 [18]							*↑								*↓										*↓				

Study	Lactobacillus	Latilactobacillus	Ligilactobacillus	Limosilactobacillus	Weissella	Faecalibacterium	Ruminococcus	Anaerococcus	Finegoldia	Parvimonas	Peptophilus	Peptostreptococcus	Romboutsia	Megamonas	Mitsuokella	Schwartzia	Selenomonas	Streptococcus	Vallitalea	Allisonella	Anaeroglobus	Dialister	Megasphaera	Veillonella	proportion of patients	dose
Family	Lactobacillaceae					Oscillospiraceae (Ruminococcaceae)		Peptoniphilaceae				Peptostreptococcaceae		Selenomonadaceae				Streptococcaceae	Vallitaleaceae	Veillonellaceae						
**Oral – Patients on corticosteroids**																										
Liu et al. 2021 [53]																	↑	↓						↑	30/31 pred.	7,5 mg/d
Correa et al 2017 [42]																	↑	↓							N/A	N/A
Li et al. 2020 [48]	↑									↓												↓		↑	N/A	N/A
**Oral – Patients not on corticosteroids**																									absence prior study	
Guo et al. 2023 [44]												↓						↓						↑	N/A	
**Gut – Patients on corticosteroids**																									proportion of patients	dose
Liu et al. 2021 [53]	↑					↓								↓				↑						↑	30/31 pred.	7,5 mg/d
Li et al. 2019 [50]	↑					↓												↑					↑		33/40 glucocort.	N/A
Ling et al. 2023 [52]	↑					↓		↑	↑	↑				↓				↑				↓			26/30 pred.	avg. 24,4 mg/d
Azzouz et al. 2023 [40]																								↑	8/16 pred.	avg. 23,75 mg/d
Azzouz et al. 2019 [39]							↑											↑				↑	↑		22/61 pred + 1/26 methylpred	avg. 11,15 mg/d +20 mg/d
Toumi et al. 2022 [60]																									4/16 pred.	N/A
Van der Meulen et al. 2019 [63]													↓					↑				↓			5/28 corticost.	max. 7,5 mg/d
**Gut – Patients not on corticosteroids**																									absence prior study	
Hevia et al. 2014 [45]																									6 months	
Jia et al. 2023 [46]																								*↑	3 months	
Wei et al. 2019 [65]							↓											↑							1 month	
Chen et al. 2021 [18]				*↑														*↑						*↑	N/A	

Study-level findings of relative abundance of microbes at the genus level based on corticosteroid intake of patients

Systemic lupus erythematosus

Phylum	Bacteroidota (Bacteroidetes)																		Campylobacterota	Cyanobacteriota (Cyanobacteria)	Fusobacteriota (Fusobacteria)			Mycoplasmatota (Tenericutes)
Family	Bacteroidaceae		Barnesiellaceae		Flavobacteriaceae	Odoribacteraceae	Porphyromonadaceae	Prevotellaceae						Rikenellaceae	Tannerellaceae		Weeksellaceae		Campylobacteraceae		Fusobacteriaceae	Leptotrichiaceae		Mycoplasmataceae
Genus	Bacteroides	Phocaeicola	Barnesiella	Coprobacter	Capnocytophaga	Odoribacter	Porphyromonas	Alloprevotella	Hoylesella	Leyella	Paraprevotella	Prevotella	Segatella	Alistipes	Parabacteroides	Tannerella	Bergeyella	Chryseobacterium	Campylobacter		Fusobacterium	Leptotrichia	Sneathia	Mycoplasma
**Oral – Patients on corticosteroids**																								
Liu et al. 2021 [53]																					↑ (dark green)			
Correa et al 2017 [42]												↑ (light green)	* ↑ (light green)											
Li et al. 2020 [48]			↑ (dark green)									* ↑ (light green)				↓ (dark red)		↓ (dark red)						↓ (dark red)
**Oral – Patients not on corticosteroids**																								
Guo et al. 2023 [44]							↓ (dark red)	↑ (dark green)				↑ (dark green)										↑ (dark green)		
**Gut – Patients on corticosteroids**																								
Liu et al. 2021 [53]	↓ (dark red)											↑ (dark green)											↑ (dark green)	
Li et al. 2019 [50]																			↑ (light green)					
Ling et al. 2023 [52]	↓ (dark red)											↓ (light red)										↑ (light green)		
Azzouz et al. 2023 [40]	* ↑ (light green)																				↑ (light green)			
Azzouz et al. 2019 [39]	=																							
Toumi et al. 2022 [60]														↑ (dark green)	↑ (dark green)									
Van der Meulen et al. 2019 [63]	↑ (dark green)	* ↑ (light green)	↑ (dark green)											↑ (dark green)								↑ (dark green)		
**Gut – Patients not on corticosteroids**																								
Hevia et al. 2014 [45]	↑ (dark green)																							
Jia et al. 2023 [46]																								
Wei et al. 2019 [65]							↓ (dark red)				↓ (dark red)	↓ (dark red)												
Chen et al. 2021 [18]	* ↓ (light red)																							

Phylum	Pseudomonadota (Proteobacteria)																												
Family	Acetobacteraceae	Brucellaceae	Burkholderiaceae		Caulobacteraceae	Comamonadaceae			Enterobacteriaceae				Halomonadaceae		Hyphomicrobiaceae	Lysobacteraceae	Moraxellaceae	Neisseriaceae			Oxalobacteraceae	Pasteurellaceae			Pseudomonadaceae	Roseobacteraceae	Sphaerotilaceae	Sphingomonadaceae	
Genus	Acetobacter	Pseudochrobactrum	Lautropia	Ralstonia	Caulobacter	Delftia	Comamonas	Limnohabitans	Citrobacter	Enterobacter	Escherichia	Klebsiella	Halomonas	Vreelandella			Acinetobacter	Eikenella	Kingella	Neisseria		Actinobacillus	Haemophilus	Mannheimia	Pseudomonas	Octadecabacter	Roseateles (Mitsuaria)	Novosphingobium	Sphingomonas
**Oral – Patients on corticosteroids**																													
Liu et al. 2021 [53]			↑ (dark green)																	↓ (light red)			↓ (light red)						
Correa et al 2017 [42]																							↓ (light red)		↑ (light green)				↓ (light red)
Li et al. 2020 [48]					↓ (dark red)								↓ (dark red)				↓ (dark red)	↓ (dark red)	↓ (dark red)									↓ (dark red)	↓ (dark red)
**Oral – Patients not on corticosteroids**																													
Guo et al. 2023 [44]													↓ (dark red)							=			↓ (dark red)						↑ (dark green)
**Gut – Patients on corticosteroids**																													
Liu et al. 2021 [53]																													
Li et al. 2019 [50]																													
Ling et al. 2023 [52]										↓ (dark red)																			
Azzouz et al. 2023 [40]																													
Azzouz et al. 2019 [39]																													
Toumi et al. 2022 [60]																													
Van der Meulen et al. 2019 [63]																													
**Gut – Patients not on corticosteroids**																													
Hevia et al. 2014 [45]																													
Jia et al. 2023 [46]																													
Wei et al. 2019 [65]																													
Chen et al. 2021 [18]																													

Phylum	Spirochaetota (Spirochaetes)	Synergistota (Synergistetes)		Thermodesulfobacteria		Verrucomicrobiota (Verrucomicrobia)			
Family	Treponemataceae	Aminobacteriaceae	Dethiosulfovibrionaceae	Desulfovibrionaceae		Akkermansiaceae	Verrucomicrobiaceae		
Genus	Treponema	Fretibacterium	Pyramidobacter	Bilophila	Desulfovibrio	Akkermansia			
**Oral – Patients on corticosteroids**								**proportion of patients**	**dose**
Liu et al. 2021 [53]								30/31 pred.	7,5 mg/d
Correa et al 2017 [42]	↑ (light green)	↑ (light green)						N/A	N/A
Li et al. 2020 [48]	* ↓ (light red)		↑ (dark green)					N/A	N/A
**Oral – Patients not on corticosteroids**								**abscence prior study**	
Guo et al. 2023 [44]								N/A	
**Gut – Patients on corticosteroids**								**proportion of patients**	**dose**
Liu et al. 2021 [53]								30/31 pred.	7,5 mg/d
Li et al. 2019 [50]								33/40 glucocort.	N/A
Ling et al. 2023 [52]								26/30 pred.	avg. 24,4 mg/d
Azzouz et al. 2023 [40]								8/16 pred.	avg. 23,75 mg/d
Azzouz et al. 2019 [39]						↑ (light green)		22/61 pred + 1/26 methylpre	avg. 11,15 mg/d + 20mg/d
Toumi et al. 2022 [60]								4/16 pred.	N/A
Van der Meulen et al. 2019 [63]								5/28 corticost.	max. 7,5 mg/d
**Gut – Patients not on corticosteroids**								**abscence prior study**	
Hevia et al. 2014 [45]					↓ (dark red)			6 months	
Jia et al. 2023 [46]								3 months	
Wei et al. 2019 [65]								1 month	
Chen et al. 2021 [18]								N/A	

Study-level findings of relative abundance of microbes at the genus level based on corticosteroid intake of patients

Sjögren's syndrome

Phylum: Actinomycetota (Actinobacteria)

Study	Actinomyces	Mobiluncus	Schaalia	Atopobium	Lancefieldella	Olsenella	Alloscardovia	Bifidobacterium	Gardnerella	Parascardovia	Scardovia	Collinsella	Corynebacterium	Adlercreutzia	Eggerthella	Gordonibacter	Leucobacter	Rothia	
Family	Actinomycetaceae			Atopobiaceae			Bifidobacteriaceae					Coriobacteriaceae	Corynebacteriaceae	Eggerthellaceae			Microbacteriaceae	Micrococcaceae	Streptomycetaceae
**Oral – Patients on corticosteroids**																			
Li et al. 2016 [49]																↑			
Sharma et al. 2020 [58]			↑		↑		↑			↑									
Kim et al. 2022 [47]																			
Van der Meulen et al. 2018 [62]																	=		
Van der Meulen et al. 2018 [61]			↑			↑	↑												
Martinez-Nava et al. 2023 [55]																			
**Oral – Patients not on corticosteroids**																			
Alam et al. 2020 [38]			↑																
Zhou et al. 2018 [69]																			
Xie et al. 2024 [67]												↑							
**Gut – Patients on corticosteroids**																			
Mandl et al. 2017 [54]							↓												
Van der Meulen et al. 2019 [63]							↓				↓								
**Gut – Patients not on corticosteroids**																			
Jia et al. 2023 [46]							*↑												
Cano-Ortiz et al. 2020 [41]							↓				↑								
Wu et al. 2019 [66]							↑												
Yang et al. 2022 [68]																			

Phylum: Bacillota (Firmicutes)

Study	Phascolarctobacterium	Abiotrophia	Granulicatella	Clostridium	Thomosclavelia		Enterococcus	Dielma	Eubacterium	Filifactor	Gemella	Agathobacter	Anaerobutyricum	Blautia	Coprococcus	Dorea	Enterocloster	Johnsonella	Lachnoanaerobaculum	Lachnoclostridium	Lachnospira	Mediterraneibacter	Otoolea	Oribacterium	Roseburia	Shuttleworthella	Solobacterium	Stomatobaculum	Tyzzerella
Family	Acidaminococcaceae	Aerococcaceae	Carnobacteriaceae	Clostridiaceae	Coprobacillaceae	Defluviitaleceaea	Enterococcaceae	Erysipelotrichaceae	Eubacteriaceae	Filifactoraceae	Gemellaceae	Lachnospiraceae																	
**Oral – Patients on corticosteroids**																													
Li et al. 2016 [49]			↓																										
Sharma et al. 2020 [58]		↓																											
Kim et al. 2022 [47]																													
Van der Meulen et al. 2018 [62]																													
Van der Meulen et al. 2018 [61]		↓	↓				↓				↑																		
Martinez-Nava et al. 2023 [55]																								↑					
**Oral – Patients not on corticosteroids**																													
Alam et al. 2020 [38]												↑																	
Zhou et al. 2018 [69]																													
Xie et al. 2024 [67]																													
**Gut – Patients on corticosteroids**																													
Mandl et al. 2017 [54]																													
Van der Meulen et al. 2019 [63]														=		=					↑								
**Gut – Patients not on corticosteroids**																													
Jia et al. 2023 [46]																													
Cano-Ortiz et al. 2020 [41]				↑			↑							↓		↓					↓				↓				
Wu et al. 2019 [66]				↓																									
Yang et al. 2022 [68]				↓			↑																						

Study	Lactobacillus	Latilactobacillus	Ligilactobacillus	Limosilactobacillus	Weissenella	Faecalibacterium	Ruminococcus	Anaerococcus	Finegoldia	Parvimonas	Peptophilus	Peptostreptococcus	Romboutsia	Megamonas	Mitsuokella	Schwartzia	Selenomonas	Streptococcus	Vallitalea	Allisonella	Anaeroglobus	Dialister	Megasphaera	Veillonella	proportion of patients / abscence prior study	dose
Family	Lactobacillaceae					Oscillospiraceae (Ruminococcaceae)		Peptoniphiliaceae				Peptostreptococcaceae		Selenomonadaceae				Streptococcaceae	Vallitaleaceae	Veillonellaceae						
**Oral – Patients on corticosteroids**																									proportion of patients	dose
Li et al. 2016 [49]																									4/10 pred.	10-20 mg/d
Sharma et al. 2020 [58]	↑			↑												↑	↑					↑			11/37 ster.	N/A
Kim et al. 2022 [47]												↑											=		4/23 corticost.	N/A
Van der Meulen et al. 2018 [62]				↑													↑	↓							4/23 corticost.	N/A
Van der Meulen et al. 2018 [61]	↑						↓			↑		↑						↓		↑		↑		↑	5/37 corticost.	N/A
Martinez-Nava et al. 2023 [55]	↓																↓	↑						↑	5/45 pred.	N/A
**Oral – Patients not on corticosteroids**																									abscence prior study	
Alam et al. 2020 [38]				↑														↑							1 month	
Zhou et al. 2018 [69]	↓																								N/A	
Xie et al. 2024 [67]	=																	↓						↑	N/A	
**Gut – Patients on corticosteroids**																									proportion of patients	dose
Mandl et al. 2017 [54]																									36/42 glucocort.	avg. 5mg/d
Van der Meulen et al. 2019 [63]						↑	↑						↓					↑				↓			2/36 corticost.	N/A
**Gut – Patients not on corticosteroids**																									abscence prior study	
Jia et al. 2023 [46]							*↓																	*↑	3 months	
Cano-Ortiz et al. 2020 [41]						↓	↓											↑							3 months	
Wu et al. 2019 [66]						↑																			2 months	
Yang et al. 2022 [68]						↓												↑				↓	↑	↑	N/A	

Study-level findings of relative abundance of microbes at the genus level based on corticosteroid intake of patients

phylum

Sjögren's syndrome

Study	Bacteroides	Phocaeicola	Barnesiella	Coprobacter	Capnocytophaga	Odoribacter	Porphyromonas	Alloprevotella	Hoylesella	Leyella	Paraprevotella	Prevotella	Segatella	Alistipes	Parabacteroides	Tannerella	Bergeyella	Chryseobacterium
Phylum	Bacteroidota (Bacteroidetes)																	
Family	Bacteroidaceae		Barnesiellaceae		Flavobacteriaceae	Odoribacteraceae	Porphyromonadaceae	Prevotellaceae						Rikenellaceae	Tannerellaceae		Weeksellaceae	
**Oral** – Patients on corticosteroids																		
Li et al. 2016 [49]																		
Sharma et al. 2020 [58]																		
Kim et al. 2022 [47]																		
Van der Meulen et al. 2018 [62]																		
Van der Meulen et al. 2018 [61]								↓										↓
Martinez-Nava et al. 2023 [55]							↑					↑						
**Oral** – Patients not on corticosteroids																		
Alam et al. 2020 [38]					*↓							↑						
Zhou et al. 2018 [69]												↑						
Xie et al. 2024 [67]								↑				↑						
**Gut** – Patients on corticosteroids																		
Mandl et al. 2017 [54]														↓				
Van der Meulen et al. 2019 [63]	↑	*↑	↑											↑				
**Gut** – Patients not on corticosteroids																		
Jia et al. 2023 [46]													*↓					
Cano-Ortiz et al. 2020 [41]	↓											↑		↓	↓			
Wu et al. 2019 [66]	↑											↑						
Yang et al. 2022 [68]	↑											↓						

Study	Campylobacter		Fusobacterium	Leptotrichia	Sneathia	Mycoplasma
Phylum	Campylobacterota	Cyanobacteriota (Cyanobacteria)	Fusobacteriota (Fusobacteria)			Mycoplasmato (Tenericu
Family	Campylobacteraceae		Fusobacteriaceae	Leptotrichiaceae		Mycoplasmataceae
**Oral** – Patients on corticosteroids						
Li et al. 2016 [49]						
Sharma et al. 2020 [58]				↓		
Kim et al. 2022 [47]						
Van der Meulen et al. 2018 [62]			↑			
Van der Meulen et al. 2018 [61]			=	=		
Martinez-Nava et al. 2023 [55]			↑			
**Oral** – Patients not on corticosteroids						
Alam et al. 2020 [38]	↓		↓	*↓		
Zhou et al. 2018 [69]						
Xie et al. 2024 [67]	↑		↑	↑		
**Gut** – Patients on corticosteroids						
Mandl et al. 2017 [54]						
Van der Meulen et al. 2019 [63]						
**Gut** – Patients not on corticosteroids						
Jia et al. 2023 [46]						
Cano-Ortiz et al. 2020 [41]						
Wu et al. 2019 [66]						
Yang et al. 2022 [68]						

Study	Acetobacter	Pseudochrobact	Lautropia	Ralstonia	Caulobacter	Delftia	Comamonas	Limnohabitans	Citrobacter	Enterobacter	Escherichia	Klebsiella	Halomonas	Vreelandella			Acinetobacter	Eikenella	Kingella	Neisseria		Actinobacillus	Haemophilus	Mannheimia	Pseudomonas	Octadecabacter	Roseateles (Mitsuaria)	Novosphingobium	Sphingomonas
Phylum	Pseudomonadota (Proteobacteria)																												
Family	Acetobacteraceae	Brucellaceae	Burkholderiaceae		Caulobacteraceae	Comamodaceae			Enterobacteriaceae				Halomonadaceae		Hyphomicrobiaceae	Lysobacteraceae	Moraxellaceae	Neisseriaceae			Oxalobacteraceae	Pasteurellaceae			Pseudomonadaceae	Roseobacteraceae	Sphaerotilaceae	Sphingomonadaceae	
**Oral** – Patients on corticosteroids																													
Li et al. 2016 [49]		↑		↑		↓	↑	↓												↓			↓				↑		
Sharma et al. 2020 [58]																						↓		↓					
Kim et al. 2022 [47]																													
Van der Meulen et al. 2018 [62]																							↓						
Van der Meulen et al. 2018 [61]			↓																	↓			↓						
Martinez-Nava et al. 2023 [55]																													
**Oral** – Patients not on corticosteroids																													
Alam et al. 2020 [38]																				↓			↓						
Zhou et al. 2018 [69]																				↓									
Xie et al. 2024 [67]	↓																			↓			↓						
**Gut** – Patients on corticosteroids																													
Mandl et al. 2017 [54]																													
Van der Meulen et al. 2019 [63]																													
**Gut** – Patients not on corticosteroids																													
Jia et al. 2023 [46]																													
Cano-Ortiz et al. 2020 [41]											↑																		
Wu et al. 2019 [66]																													
Yang et al. 2022 [68]																													

Study	Treponema	Fretibacterium	Pyramidobacter	Bilophila	Desulfovibrio	Akkermansia		proportion of patients	dose
Phylum	Spirochaetota (Spirochaetes)	Synergistota (Synergistetes)		Thermodesulfobacteria		Verrucomicrobiota (Verrucomicrobia)			
Family	Treponemataceae	Aminobacteriaceae	Dethiosulfovibrionaceae	Desulfovibrionaceae		Akkermansiaceae	Verrucomicrobiaceae		
**Oral** – Patients on corticosteroids								**proportion of patients**	**dose**
Li et al. 2016 [49]								4/10 pred.	10-20 mg/d
Sharma et al. 2020 [58]								11/37 ster.	N/A
Kim et al. 2022 [47]								4/23 corticost.	N/A
Van der Meulen et al. 2018 [62]								4/23 corticost.	N/A
Van der Meulen et al. 2018 [61]								5/37 corticost.	N/A
Martinez-Nava et al. 2023 [55]								5/45 pred.	N/A
**Oral** – Patients not on corticosteroids								**abscence prior study**	
Alam et al. 2020 [38]								1 month	
Zhou et al. 2018 [69]								N/A	
Xie et al. 2024 [67]								N/A	
**Gut** – Patients on corticosteroids								**proportion of patients**	**dose**
Mandl et al. 2017 [54]								36/42 glucocort.	avg. 5mg/d
Van der Meulen et al. 2019 [63]								2/36 corticost.	N/A
**Gut** – Patients not on corticosteroids								**abscence prior study**	
Jia et al. 2023 [46]								3 months	
Cano-Ortiz et al. 2020 [41]								3 months	
Wu et al. 2019 [66]								2 months	
Yang et al. 2022 [68]								N/A	

Study-level findings of relative abundance of microbes at the species level based on corticosteroid intake of patients

Species	Actinomyces graevenitzii	Actinomyces johnsonii	Actinomyces massiliensis	Actinomyces oris	Actinomyces timonensis	Actinomyces viscosus		Schaalia cardiffensis		Lancefieldella parvula	Lancefieldella rimae	Olsenella profusa	Olsenella uli	Alloscardovia omnicolens	Bifidobacterium adolescentis	Bifidobacterium animalis	Bifidobacterium bifidum	Bifidobacterium breve	Bifidobacterium catenulatum	Bifidobacterium dentium	Bifidobacterium longum	Bifidobacterium scardovii	Gardnerella vaginalis	Parascardovia denticolens	Scardovia inopinata	Scardovia wiggsiae	Collinsella aerofaciens	Corynebacterium durum	Corynebacterium matruchotii	Corynebacterium propinquum	Adlercreutzia equolifaciens	Gordonibacter pamelaeae	Eggerthella lenta		Rothia aeria	Rothia dentocariosa			
**Phylum**	Actinomycetota (Actinobacteria)																																						
**Family**	Actinomycetacea								Atopobiaceae					Bifidobacteriaceae													Coriobacteriaceae	Corynebacteriaceae			Eggerthellaceae			Microbacteriaceae	Micrococcaceae		Streptomyceatceae		
**Genus**	Actinomyces						Mobiluncus	Schaalia	Atopobium	Lancefieldella		Olsenella		Alloscardovia	Bifidobacterium								Gardnerella	Parascardovia	Scardovia		Collinsella	Corynebacterium			Adlercreutzia	Gordonibacter	Eggerthela	Leucobacter	Rothia				
**Systemic lupus erythematosus**																																							
**Oral** – Patients on corticosteroids																																						**proportion of patients**	**dose**
Liu et al. 2021 [53]																																						30/31 pred.	7,5 mg/d
Correa et al 2017 [42]																																			↓			N/A	N/A
Li et al. 2020 [48]																												↓	↓									N/A	N/A
Patients not on corticosteroids																																						**abscence prior study**	
Guo et al. 2023 [44]																																						N/A	
**Gut** – Patients on corticosteroids																																						**proportion of patients**	**dose**
Liu et al. 2021 [53]															↓						↓																	30/31 pred.	7,5 mg/d
Li et al. 2019 [50]																																						33/40 glucocort.	N/A
Ling et al. 2023 [52]																																						26/30 pred.	avg. 24,4 mg/d
Azzouz et al. 2023 [40]																																						8/16 pred.	avg. 23,75 mg/d
Azzouz et al. 2019 [39]															=																							22/61 pred + 1/26 methylp.	avg. 11,15 mg/d + 20mg/d
Toumi et al. 2022 [60]																																						4/16 pred.	N/A
Van der Meulen et al. 2019 [63]																																						5/28 corticost.	max. 7,5 mg/d
Patients not on corticosteroids																																						**abscence prior study**	
Hevia et al. 2014 [45]																																						6 months	
Jia et al. 2023 [46]																				↑							↓				↑							3 months	
Wei et al. 2019 [65]																																						1 month	
Chen et al. 2021 [18]	↑	↑	↑	↑	↑	↑		↑		↑	↑	↑	↑	↑				↑		↑			↑	↑	↑	↑	↓	↑		↑	↑		↑		↑	↑		N/A	

Study-level findings of relative abundance of microbes at the species level based on **corticosteroid intake of patients**

Systemic lupus erythematosus

Species		Abiotrophia defectiva	Granulicatella elegans	Thomosclavelia ramosa	Clostridium perfringens		Enterococcus avium	Enterococcus casseliflavus	Dielma fastidiosa	Holdemanella biformis	Eubacterium limosum	Eubacterium ruminatum	Eubacterium ventriosum	Filifactor alocis	Gemella morbillorum	Agathobacter rectalis	Anaerobutyricum hallii	Blautia faecis	Blautia obeum	Blautia wexlerae	Coprococcus catus	Coprococcus comes	Coprococcus eutactus	Dorea longicatena	Enterocloster aldenensis	Enterocloster boltae	Enterocloster clostridiformis	Johnsonella ignava	Lachnoanaerobaculum orale	Clostridium scindens	Lachnospira eligens	Mediterraneibacter gnavus	Ruminococcus lactaris	Ruminococcus torques	Clostridium symbiosum	Oribacterium asaccharolyticum	Roseburia hominis	Roseburia intestinalis	Roseburia inulinivorans	Shuttleworthella satelles		Stomatobaculum longum	Clostridium nexile	Lactobacillus crispatus	Lactobacillus delbrueckii	Lactobacillus gasseri	Lactobacillus iners	Lactobacillus johnsonii	Latilactobacillus sakei	Ligilactobacillus ruminis	Ligilactobacillus salivarius	Limosilactobacillus fermentum	Limosilactobacillus mucosae	Limosilactobacillus oris	Limosilactobacillus reuteri	Limosilactobacillus vaginalis		Faecalibacterium prausnitzii	Ruminococcus bromii	Ruminococcus callidus					Intestinibacter bartlettii			Megamonas hypermegale	Megamonas rupellensis	Mitsuokella multacida		Selenomonas noxia	Selenomonas ruminatum	Streptococcus anginosus	Streptococcus australis	Streptococcus constellatus	Streptococcus cristatus	Streptococcus infantis	Streptococcus intermedius	Streptococcus lutetiensis	Streptococcus mutans	Streptococcus oralis	Streptococcus parasanguinis	Streptococcus sanguinis	Streptococcus sobrinus	Streptococcus suis	Vallitalea pronyensis	Anaeroglobus geminatus	Dialister invisus	Dialister succinatiphilus	Megasphaera micronuciformis	Veillonella atypica	Veillonella dispar	Veillonella parvula	Veillonella rodentium	Veillonella rogosae		
Phylum	Bacillota (Firmicutes)																																																																																																
Family	Acidaminococcaceae	Aerococcaceae	Carnobacteriaceae	Coprobacillaceae	Clostridiaceae	Defluviitaleaceae	Enterococcaceae		Erysipelotrichaceae		Eubacteriaceae			Filifactoraceae	Gemellaceae	Lachnospiraceae																												Lactobacillaceae														Oscillospiraceae (Ruminococcaceae)			Peptoniphilaceae				Peptostreptococcaceae			Selenomonadaceae						Streptococcaceae													Vallitaleaceae	Veillonellaceae									
Genus	Phascolarctobacterium	Abiotrophia	Granulicatella	Thomosclavelia	Clostridium		Enterococcus		Dielma	Holdemanella	Eubacterium			Filifactor	Gemella	Agathobacter	Anaerobutyricum	Blautia			Coprococcus			Dorea	Enterocloster			Johnsonella	Lachnoanaerobaculum	Lachnoclostridium	Lachnospira	Mediterraneibacter			Otoolea	Oribacterium	Roseburia			Shuttleworthella	Solobacterium	Stomatobaculum	Tyzzerella	Lactobacillus					Latilactobacillus	Ligilactobacillus		Limosilactobacillus					Weissenella	Faecalibacterium	Ruminococcus		Anaerococcus	Finegoldia	Peptophilus	Parvimonas	Intestinibacter	Peptostreptococcus	Romboutsia	Megamonas		Mitsuokella -	Schwartzia	Selenomonas		Streptococcus													Vallitalea	Anaeroglobus	Dialister		Megasphaera	Veillonella						
**Oral** – Patients on corticosteroids																																																																																																				proportion of patients	dose
Liu et al. 2021 [53]																																																																												↓																								30/31 pred.	7,5 mg/d
Correa et al 2017 [42]																																																																									↑												↓				↑											N/A	N/A
Li et al. 2020 [48]														↓														↓																									↑																																						↓								N/A	N/A
Patients not on corticosteroids																																																																																																			absence prior study	
Guo et al. 2023 [44]																																																																																																		N/A	
**Gut** – Patients on corticosteroids																																																																																																			proportion of patients	dose
Liu et al. 2021 [53]																																															↑																																																			30/31 pred.	7,5 mg/d
Li et al. 2019 [50]																																																					↑					↓																↑																				↑				33/40 glucocort.	N/A
Ling et al. 2023 [52]																																																																																																		26/30 pred.	avg. 24,4 mg/d
Azzouz et al. 2023 [40]																																↑																																																																	8/16 pred.	avg. 23,75 mg/d
Azzouz et al. 2019 [39]																																↑																																																																	22/61 pred + 1/26 methylp.	avg. 11,15 mg/d + 20mg/d
Toumi et al. 2022 [60]																																																																																																			4/16 pred.	N/A
Van der Meulen et al. 2019 [63]																																																																																																			5/28 corticost.	max. 7,5 mg/d
Patients not on corticosteroids																																																																																																				absence prior study	
Hevia et al. 2014 [45]																																																																																																			6 months	
Jia et al. 2023 [46]				↑																		↓										↑		↑				↓													↑							↓		↓					↑									↑	↓						↑		↑										↑	↑			3 months	
Wei et al. 2019 [65]																																																																																																			1 month	
Chen et al. 2021 [18]		↑	↑	↑			↑	↑			↑		↓		↑	↓	↑		↓		↓	↓		↓						↑	↓	↑	↓	↑			↓	↓	↓	↑			↑	↑	↓	↑					↑		↑		↑			↓		↓					↑				↓	↓				↑		↑	↑		↑	↑		↑	↑						↑		↑		↑	↑			N/A	

Study-level findings of relative abundance of microbes at the species level based on corticosteroid intake of patients

Systemic lupus erythematosus

Species	Bacteroides caccae	Bacteroides cellulosilyticus	Bacteroides clarus	Bacteroides eggerthii	Bacteroides fragilis	Bacteroides intestinalis	Bacteroides ovatus	Bacteroides salyersiae	Bacteroides thetaiotaomicron	Bacteroides uniformis	Phocaeicola coprocola	Phocaeicola dorei	Phocaeicola plebeius	Phocaeicola vulgatus	Barnesiella intestinihominis	Coprobacter fastidiosus
Phylum	Bacteroidota (Bacteroidetes)															
Family	Bacteroidaceae														Barnesiellaceae	
Genus	Bacteroides										Phocaeicola				Barnesiella	Coprobacter
**Oral** – Patients on corticosteroids																
Liu et al. 2021 [53]																
Correa et al 2017 [42]																
Li et al. 2020 [48]																
Patients not on corticosteroids																
Guo et al. 2023 [44]																
**Gut** – Patients on corticosteroids																
Liu et al. 2021 [53]																
Li et al. 2019 [50]																
Ling et al. 2023 [52]																
Azzouz et al. 2023 [40]																
Azzouz et al. 2019 [39]					↑					↓						
Toumi et al. 2022 [60]																
Van der Meulen et al. 2019 [63]							↑		↑	↑		↑	↑	↑		
Patients not on corticosteroids																
Hevia et al. 2014 [45]																
Jia et al. 2023 [46]					↑								↓			
Wei et al. 2019 [65]																
Chen et al. 2021 [18]		↓	↓	↓	↑	↓		↓					↓	↑		↓

Species	Capnocytophaga gingivalis	Capnocytophaga leadbetteri	Odoribacter splanchnicus	Porphyromonas gingivalis		Hoylesella enoeca	Hoylesella nanceiensis	Hoylesella pleuritidis	Hoylesella shahii	Leyella stercorea	Paraprevotella clara	Prevotella genomosp	Prevotella histicola	Prevotella melaninogenica	Prevotella micans	Prevotella nigrescens
Phylum	Bacteroidota (Bacteroidetes)															
Family	Flavobacteriaceae		Odoribacteraceae	Porphyromonadaceae	Prevotellaceae											
Genus	Capnocytophaga		Odoribacter	Porphyromonas	Alloprevotella	Hoylesella				Leyella	Paraprevotella	Prevotella				
**Oral** – Patients on corticosteroids																
Liu et al. 2021 [53]																
Correa et al 2017 [42]	↓							↑								↑
Li et al. 2020 [48]						↑	↓	↑				↑			↑	
Patients not on corticosteroids																
Guo et al. 2023 [44]																
**Gut** – Patients on corticosteroids																
Liu et al. 2021 [53]																
Li et al. 2019 [50]																
Ling et al. 2023 [52]																
Azzouz et al. 2023 [40]																
Azzouz et al. 2019 [39]																
Toumi et al. 2022 [60]																
Van der Meulen et al. 2019 [63]																
Patients not on corticosteroids																
Hevia et al. 2014 [45]																
Jia et al. 2023 [46]											↓					
Wei et al. 2019 [65]																
Chen et al. 2021 [18]											↓					

Species	Segatella copri	Segatella oris	Segatella oulorum	Segatella salivae	Alistipes finegoldii	Alistipes onderdonkii	Alistipes putredinis	Alistipes senegalensis	Alistipes shahii	Parabacteroides distasonis	Parabacteroides merdae			Chryseobacterium taiwanense	Campylobacter coli	Campylobacter concisus	Campylobacter gracilis	Campylobacter showae	
Phylum	Bacteroidota (Bacteroidetes)														Campylobacterota				Cyanobacteriota (Cyanobacteria)
Family	Prevotellaceae								Rikenellaceae	Tannerellaceae			Weeksellaceae		Campylobacteraceae				
Genus	Segatella								Alistipes	Parabacteroides		Tannerella	Bergeyella	Chryseobacterium	Campylobacter				
**Oral** – Patients on corticosteroids																			
Liu et al. 2021 [53]																			
Correa et al 2017 [42]		↑	↑																
Li et al. 2020 [48]														↓					
Patients not on corticosteroids																			
Guo et al. 2023 [44]																			
**Gut** – Patients on corticosteroids																			
Liu et al. 2021 [53]																			
Li et al. 2019 [50]																			
Ling et al. 2023 [52]																			
Azzouz et al. 2023 [40]																			
Azzouz et al. 2019 [39]	↑																		
Toumi et al. 2022 [60]						↑													
Van der Meulen et al. 2019 [63]																			
Patients not on corticosteroids																			
Hevia et al. 2014 [45]																			
Jia et al. 2023 [46]																			
Wei et al. 2019 [65]																			
Chen et al. 2021 [18]	↓				↑	↑			↓		↓								

Study	proportion of patients / abscence prior study	dose
**Oral** – Patients on corticosteroids	**proportion of patients**	**dose**
Liu et al. 2021 [53]	30/31 pred.	7,5 mg/d
Correa et al 2017 [42]	N/A	N/A
Li et al. 2020 [48]	N/A	N/A
Patients not on corticosteroids	**abscence prior study**	
Guo et al. 2023 [44]	N/A	
**Gut** – Patients on corticosteroids	**proportion of patients**	**dose**
Liu et al. 2021 [53]	30/31 pred.	7,5 mg/d
Li et al. 2019 [50]	33/40 glucocort.	N/A
Ling et al. 2023 [52]	26/30 pred.	avg. 24,4 mg/d
Azzouz et al. 2023 [40]	8/16 pred.	avg. 23,75 mg/d
Azzouz et al. 2019 [39]	22/61 pred + 1/26 methylp	avg. 11,15 mg/d + 20mg/c
Toumi et al. 2022 [60]	4/16 pred.	N/A
Van der Meulen et al. 2019 [63]	5/28 corticost.	max. 7,5 mg/d
Patients not on corticosteroids	**abscence prior study**	
Hevia et al. 2014 [45]	6 months	
Jia et al. 2023 [46]	3 months	
Wei et al. 2019 [65]	1 month	
Chen et al. 2021 [18]	N/A	

Study-level findings of relative abundance of microbes at the species level based on corticosteroid intake of patients

Systemic lupus erythematosus

**Phylum**	Fusobacteriota (Fusobacteria)								Mycoplasmatota (Tenericutes)
**Family**	Fusobacteriaceae				Leptotrichiaceae				Mycoplasmataceae
**Genus**	Fusobacterium				Leptotrichia			Sneathia	Mycoplasma
**Species**	Fusobacterium nucleatum	Fusobacterium periodonticum	Fusobacterium ulcerans	Fusobacterium varium	Leptotrichia hofstadii	Leptotrichia hongkongensis	Leptotrichia wadei	Sneathia amnii	Mycoplasma ovis
**Oral – Patients on corticosteroids**									
Liu et al. 2021 [53]									
Correa et al 2017 [42]									
Li et al. 2020 [48]									↓
**Oral – Patients not on corticosteroids**									
Guo et al. 2023 [44]									
**Gut – Patients on corticosteroids**									
Liu et al. 2021 [53]									
Li et al. 2019 [50]									
Ling et al. 2023 [52]									
Azzouz et al. 2023 [40]									
Azzouz et al. 2019 [39]									
Toumi et al. 2022 [60]									
Van der Meulen et al. 2019 [63]									
**Gut – Patients not on corticosteroids**									
Hevia et al. 2014 [45]									
Jia et al. 2023 [46]									
Wei et al. 2019 [65]									
Chen et al. 2021 [18]	↑								

**Phylum**	Pseudomonadota (Proteobacteria)																	
**Family**	Acetobacteraceae	Brucellaceae	Burkholderiaceae		Caulobacteraceae	Comamodaceae			Enterobacteriaceae					Halomonadaceae		Hyphomicrobiaceae	Lysobacteraceae	Moraxellaceae
**Genus**	Acetobacter	Pseudochrobactrum	Lautropia	Ralstonia	Caulobacter	Delftia	Comamonas	Limnohabitans	Citrobacter	Enterobacter	Escherichia		Klebsiella	Halomonas	Vreenlandella			Acinetobacter
**Species**			Lautropia mirabilis				Comamonas testosteroni		Citrobacter koseri		Escherichia coli	Escherichia fergusonii	Klebsiella pneumoniae		Vreenlandella hamiltonii			
**Oral – Patients on corticosteroids**																		
Liu et al. 2021 [53]																		
Correa et al 2017 [42]																		
Li et al. 2020 [48]							↑											
**Oral – Patients not on corticosteroids**																		
Guo et al. 2023 [44]																		
**Gut – Patients on corticosteroids**																		
Liu et al. 2021 [53]																		
Li et al. 2019 [50]																		
Ling et al. 2023 [52]																		
Azzouz et al. 2023 [40]																		
Azzouz et al. 2019 [39]																		
Toumi et al. 2022 [60]																		
Van der Meulen et al. 2019 [63]																		
**Gut – Patients not on corticosteroids**																		
Hevia et al. 2014 [45]																		
Jia et al. 2023 [46]																		
Wei et al. 2019 [65]																		
Chen et al. 2021 [18]											↑							

**Phylum**	Pseudomonadota (Proteobacteria)																		
**Family**	Neisseriaceae						Oxalobacteraceae	Pasteurellaceae						Pseudomonadaceae		Roseobacteraceae	Sphaerotilaceae	Sphingomonadaceae	
**Genus**	Eikenella	Kingella	Neisseria					Actinobacillus	Haemophilus				Mannheimia	Pseudomonas		Octadecabacter	Roseateles	Novosphingobium	Sphingomonas
**Species**	Eikenella corrodens		Neisseria elongata	Neisseria oralis	Neisseria perflava	Neisseria subflava			Haemophilus haemolyticus	Haemophilus influenzae	Haemophilus parainfluenzae	Haemophilus sputorum		Pseudomonas aeruginosa	Pseudomonas graminis	Octadecabacter arcticus			
**Oral – Patients on corticosteroids**																			
Liu et al. 2021 [53]																			
Correa et al 2017 [42]											↓								
Li et al. 2020 [48]															↓				
**Oral – Patients not on corticosteroids**																			
Guo et al. 2023 [44]																			
**Gut – Patients on corticosteroids**																			
Liu et al. 2021 [53]																			
Li et al. 2019 [50]																			
Ling et al. 2023 [52]																			
Azzouz et al. 2023 [40]																			
Azzouz et al. 2019 [39]																			
Toumi et al. 2022 [60]																			
Van der Meulen et al. 2019 [63]																			
**Gut – Patients not on corticosteroids**																			
Hevia et al. 2014 [45]																			
Jia et al. 2023 [46]																			
Wei et al. 2019 [65]																			
Chen et al. 2021 [18]	↑									↑									

**Phylum**	Spirochaetota (Spirochaetes)			Synergistota (Synergistetes)		Thermodesulfobacteria			Verrucomicrobiota (Verrucomicrobia)			
**Family**	Treponemataceae			Aminobacteriaceae	Dethiosulfovibrionaceae	Desulfovibrionaceae			Akkermansiaceae	Verrucomicrobiaceae		
**Genus**	Treponema			Fretibacterium	Pyramidobacter	Bilophila	Desulfovibrio		Akkermansia			
**Species**	Treponema denticola	Treponema lecithinolyticum	Treponema maltophilum	Fretibacterium fastidiosum	Pyramidobacter piscolens	Bilophila wadsworthia	Desulfovibrio legallii	Desulfovibrio piger	Akkermansia muciniphila			
**Oral – Patients on corticosteroids**											**proportion of patients**	**dose**
Liu et al. 2021 [53]											30/31 pred.	7,5 mg/d
Correa et al 2017 [42]			↑	↑							N/A	N/A
Li et al. 2020 [48]	↓	↓			↑						N/A	N/A
**Oral – Patients not on corticosteroids**											**abscence prior study**	
Guo et al. 2023 [44]											N/A	
**Gut – Patients on corticosteroids**											**proportion of patients**	**dose**
Liu et al. 2021 [53]											30/31 pred.	7,5 mg/d
Li et al. 2019 [50]											33/40 glucocort.	N/A
Ling et al. 2023 [52]											26/30 pred.	avg. 24,4 mg/d
Azzouz et al. 2023 [40]											8/16 pred.	avg. 23,75 mg/d
Azzouz et al. 2019 [39]									=		22/61 pred + 1/26 methylp	avg. 11,15 mg/d + 20mg/d
Toumi et al. 2022 [60]											4/16 pred.	N/A
Van der Meulen et al. 2019 [63]											5/28 corticost.	max. 7,5 mg/d
**Gut – Patients not on corticosteroids**											**abscence prior study**	
Hevia et al. 2014 [45]											6 months	
Jia et al. 2023 [46]						↓					3 months	
Wei et al. 2019 [65]											1 month	
Chen et al. 2021 [18]									↑		N/A	

Study-level findings of relative abundance of microbes at the species level based on corticosteroid intake of patients

Sjögren's syndrome

Phylum	Actinomycetota (Actinobacteria)																																						
Family	Actinomycetacea								Atopobiaceae					Bifidobacteriaceae													Coriobacteriaceae	Corynebacteriaceae			Eggerthellaceae			Microbacteriaceae	Micrococcaceae		Streptomyceatceae		
Genus	Actinomyces						Mobiluncus	Schaalia	Atopobium	Lancefieldella		Olsenella		Alloscardovia	Bifidobacterium								Gardnerella	Parascardovia	Scardovia		Collinsella	Corynebacterium			Adlercreutzia	Gordonibacter	Eggerthella	Leucobacter	Rothia				
Species	Actinomyces graevenitzi	Actinomyces johnsonii	Actinomyces massiliensis	Actinomyces oris	Actinomyces timonensis	Actinomyces viscosus		Schaalia cardiffensis		Lancefieldella parvula	Lancefieldella rimae	Olsenella profusa	Olsenella uli	Alloscardovia omnicolens	Bifidobacterium adolescentis	Bifidobacterium animalis	Bifidobacterium bifidum	Bifidobacterium breve	Bifidobacterium catenulatum	Bifidobacterium dentium	Bifidobacterium longum	Bifidobacterium scardovii	Gardnerella vaginalis	Parascardovia denticolens	Scardovia inopinata	Scardovia wiggsiae	Collinsella aerofaciens	Corynebacterium durum	Corynebacterium matruchotii	Corynebacterium propinquum	Adlercreutzia equolifaciens	Gordonibacter pamelaeae	Eggerthella lenta		Rothia aeria	Rothia dentocariosa			
**Oral** – Patients on corticosteroids																																						**proportion of patients**	**dose**
Li et al. 2016 [49]																																						4/10 pred.	10-20 mg/d
Sharma et al. 2020 [58]																																						11/37 ster.	N/A
Kim et al. 2022 [47]																																						4/23 corticost.	N/A
Van der Meulen et al. 2018 [62]																																						4/23 corticost.	N/A
Van der Meulen et al. 2018 [61]																																						5/37 corticost.	N/A
Martinez-Nava et al. 2023 [55]																																						5/45 pred.	N/A
**Oral** – Patients not on corticosteroids																																						**abscence prior study**	
Alam et al. 2020 [38]										↑																			↓						↓			1 month	
Zhou et al. 2018 [69]																																						N/A	
Xie et al. 2024 [67]																																						N/A	
**Gut** – Patients on corticosteroids																																						**proportion of patients**	**dose**
Mandl et al. 2017 [54]																																						36/42 glucocort.	avg. 5mg/d
Van der Meulen et al. 2019 [63]																																						2/36 corticost.	N/A
**Gut** – Patients not on corticosteroids																																						**abscence prior study**	
Jia et al. 2023 [46]														↑					↑							↓				↑								3 months	
Cano-Ortiz et al. 2020 [41]																																						3 months	
Wu et al. 2019 [66]																																						2 months	
Yang et al. 2022 [68]																																						N/A	

Study-level findings of relative abundance of microbes at the species level based on corticosteroid intake of patients

Sjögren's syndrome

Study groups (columns below): **Oral** — Patients on corticosteroids: Li et al. 2016 [49]; Sharma et al. 2020 [58]; Kim et al. 2022 [47]; Van der Meulen et al. 2018 [62]; Van der Meulen et al. 2018 [61]; Martinez-Nava et al. 2023 [55]. Patients not on corticosteroids: Alam et al. 2020 [38]; Zhou et al. 2018 [69]; Xie et al. 2024 [67]. **Gut** — Patients on corticosteroids: Mandl et al. 2017 [54]; Van der Meulen et al. 2019 [63]. Patients not on corticosteroids: Jia et al. 2023 [46]; Cano-Ortiz et al. 2020 [41]; Wu et al. 2019 [66]; Yang et al. 2022 [68].

Phylum	Family	Genus	Species	Oral: Li et al. 2016 [49]	Oral: Sharma et al. 2020 [58]	Oral: Kim et al. 2022 [47]	Oral: Van der Meulen et al. 2018 [62]	Oral: Van der Meulen et al. 2018 [61]	Oral: Martinez-Nava et al. 2023 [55]	Oral: Alam et al. 2020 [38]	Oral: Zhou et al. 2018 [69]	Oral: Xie et al. 2024 [67]	Gut: Mandl et al. 2017 [54]	Gut: Van der Meulen et al. 2019 [63]	Gut: Jia et al. 2023 [46]	Gut: Cano-Ortiz et al. 2020 [41]	Gut: Wu et al. 2019 [66]	Gut: Yang et al. 2022 [68]
Bacillota (Firmicutes)	Acidaminococcaceae	Phascolarctobacterium																
	Aerococcaceae	Abiotrophia	Abiotrophia defectiva															
	Carnobacteriaceae	Granulicatella	Granulicatella elegans															
	Coprobacillaceae	Thomosclavelia	Thomosclavelia ramosa												↑ (dark green)			
	Clostridiaceae	Clostridium	Clostridium perfringens												↑ (light green)			
	Defluviitaleceae																	
	Enterococcaceae	Enterococcus	Enterococcus avium															
			Enterococcus casseliflavus															
	Erysipelotrichaceae	Dielma	Dielma fastidiosa															
		Holdemanella	Holdemanella biformis												↓ (light red)			
	Eubacteriaceae	Eubacterium	Eubacterium limosum															
			Eubacterium ruminatum															
			Eubacterium ventriosum															
	Filifactoraceae	Filifactor	Filifactor alocis															
	Gemellaceae	Gemella	Gemella morbillorum															
	Lachnospiraceae	Agathobacter	Agathobacter rectalis															↑ (light green)
		Anaerobutyricum	Anaerobutyricum hallii															
		Blautia	Blautia faecis															
			Blautia obeum															
			Blautia wexlerae															
		Coprococcus	Coprococcus catus												↑ (light green)			
			Coprococcus comes												↓ (dark red)			
			Coprococcus eutactus															
		Dorea	Dorea longicatena													↓ (dark red)		
		Enterocloster	Enterocloster aldenensis															
			Enterocloster boltae												↑ (dark green)			
			Enterocloster clostridiformis													↑ (dark green)		
		Johnsonella	Johnsonella ignava															
		Lachnoanaerobaculum	Lachnoanaerobaculum orale							↓ (dark red)								
		Lachnoclostridium	Clostridium scindens															
		Lachnospira	Lachnospira eligens												↓ (dark red)			
		Mediterraneibacter	Mediterraneibacter gnavus												↑ (dark green)	=		
			Ruminococcus lactaris													↓ (dark red)		
			Ruminococcus torques												↓ (dark red)			
		Otoolea	Clostridium symbiosum												↑ (light green)			
		Oribacterium	Oribacterium asaccharolyticum							↓ (dark red)								
		Roseburia	Roseburia hominis															
			Roseburia intestinalis												↓ (dark red)			
			Roseburia inulinivorans															
		Shuttleworthella	Shuttleworthella satelles															
		Solobacterium																
		Stomatobaculum	Stomatobaculum longum							↓ (dark red)								
		Tyzzerella	Clostridium nexile															
	Lactobacillaceae	Lactobacillus	Lactobacillus crispatus															
			Lactobacillus delbrueckii															
			Lactobacillus gasseri															
			Lactobacillus iners															
			Lactobacillus johnsonii															
		Latilactobacillus	Latilactobacillus sakei															
		Ligilactobacillus	Ligilactobacillus ruminis															
			Ligilactobacillus salivarius							↑ (dark green)					↑ (dark green)			
		Limosilactobacillus	Limosilactobacillus fermentum															
			Limosilactobacillus mucosae															
			Limosilactobacillus oris															
			Limosilactobacillus reuteri							↑ (dark green)								
			Limosilactobacillus vaginalis															
		Weissenella																
	Oscillospiraceae (Ruminococcaceae)	Faecalibacterium	Faecalibacterium prausnitzii										↓ (light red)		↓ (dark red)	↓ (dark red)		↓ (light red)
		Ruminococcus	Ruminococcus bromii												↓ (light red)			
			Ruminococcus callidus												↓ (dark red)			
	Peptoniphilaceae	Anaerococcus																
		Finegoldia																
		Peptophilus																
		Parvimonas																
	Peptostreptococcaceae	Intestinibacter	Intestinibacter bartlettii								↑ (dark green)							
		Peptostreptococcus																
		Romboutsia																
	Selenomonadaceae	Megamonas	Megamonas hypermegale												↓ (light red)			
			Megamonas rupellensis															
		Mitsuokella -	Mitsuokella multacida															
		Schwartzia																
		Selenomonas	Selenomonas noxia															
			Selenomonas ruminatum															
	Streptococcaceae	Streptococcus	Streptococcus anginosus							↑ (dark green)					↑ (dark green)			
			Streptococcus australis												↓ (dark red)			
			Streptococcus constellatus															
			Streptococcus cristatus															
			Streptococcus infantis												↓ (light red)			
			Streptococcus intermedius							↑ (dark green)								
			Streptococcus lutetiensis															
			Streptococcus mutans							↑ (dark green)					↑ (dark green)			
			Streptococcus oralis															
			Streptococcus parasanguinis							↑ (dark green)					↑ (dark green)			
			Streptococcus sanguinis							↓ (dark red)								
			Streptococcus sobrinus							↑ (dark green)								
			Streptococcus suis															
	Vallitaleaceae	Vallitalea	Vallitalea pronyensis															
	Veillonellaceae	Anaeroglobus	Anaeroglobus geminatus															
		Dialister	Dialister invisus															
			Dialister succinatiphilus															
		Megasphaera	Megasphera micronuciformis															
		Veillonella	Veillonella atypica															
			Veillonella dispar												↑ (dark green)			
			Veillonella parvula							↑ (dark green)					↑ (dark green)			
			Veillonella rodentium							↑ (dark green)								
			Veillonella rogosae							↓ (dark red)								

Oral

Patients on corticosteroids	proportion of patients	dose
Li et al. 2016 [49]	4/10 pred.	10-20 mg/d
Sharma et al. 2020 [58]	11/37 ster.	N/A
Kim et al. 2022 [47]	4/23 corticost.	N/A
Van der Meulen et al. 2018 [62]	4/23 corticost.	N/A
Van der Meulen et al. 2018 [61]	5/37 corticost.	N/A
Martinez-Nava et al. 2023 [55]	5/45 pred.	N/A

Patients not on corticosteroids	absence prior study
Alam et al. 2020 [38]	1 month
Zhou et al. 2018 [69]	N/A
Xie et al. 2024 [67]	N/A

Gut

Patients on corticosteroids	proportion of patients	dose
Mandl et al. 2017 [54]	36/42 glucocort.	avg. 5mg/d
Van der Meulen et al. 2019 [63]	2/36 corticost.	N/A

Patients not on corticosteroids	absence prior study
Jia et al. 2023 [46]	3 months
Cano-Ortiz et al. 2020 [41]	3 months
Wu et al. 2019 [66]	2 months
Yang et al. 2022 [68]	N/A

Study-level findings of relative abundance of microbes at the species level based on corticosteroid intake of patients

Sjögren's syndrome

Species	Bacteroides caccae	Bacteroides cellulosilyticus	Bacteroides clarus	Bacteroides eggerthii	Bacteroides fragilis	Bacteroides intestinalis	Bacteroides ovatus	Bacteroides salyersiae	Bacteroides thetaiotaomicron	Bacteroides uniformis	Phocaeicola coprocola	Phocaeicola dorei	Phocaeicola plebeius	Phocaeicola vulgatus	Barnesiella intestinihominis	Coprobacter fastidiosus	Capnocytophaga gingivalis	Capnocytophaga leadbetteri	Odoribacter splanchnicus	Porphyromonas gingivalis
Phylum	Bacteroidota (Bacteroidetes)																			
Family	Bacteroidaceae														Barnesiellaceae		Flavobacteriaceae		Odoribacteraceae	Porphyromonadaceae
Genus	Bacteroides										Phocaeicola				Barnesiella	Coprobacter	Capnocytophaga		Odoribacter	Porphyromonas
**Oral – Patients on corticosteroids**																				
Li et al. 2016 [49]																				
Sharma et al. 2020 [58]																				
Kim et al. 2022 [47]																				
Van der Meulen et al. 2018 [62]																				
Van der Meulen et al. 2018 [61]																				
Martinez-Nava et al. 2023 [55]																				
**Oral – Patients not on corticosteroids**																				
Alam et al. 2020 [38]																	↓	↓		↓
Zhou et al. 2018 [69]																				
Xie et al. 2024 [67]																				
**Gut – Patients on corticosteroids**																				
Mandl et al. 2017 [54]																				
Van der Meulen et al. 2019 [63]	↑						↑			↑		↑	↑	↑						
**Gut – Patients not on corticosteroids**																				
Jia et al. 2023 [46]					↑								↓		↓					
Cano-Ortiz et al. 2020 [41]					↓															
Wu et al. 2019 [66]																				
Yang et al. 2022 [68]																				

Species		Hoylesella enoeca	Hoylesella nanceiensis	Hoylesella pleuritidis	Hoylesella shahii	Leyella stercorea	Paraprevotella clara	Prevotella genomosp	Prevotella histicola	Prevotella melaninogenica	Prevotella micans	Prevotella nigrescens	Segatella copri	Segatella oris	Segatella oulorum	Segatella salivae	Alistipes finegoldii	Alistipes onderdonkii	Alistipes putredinis	Alistipes senegalensis	Alistipes shahii	Parabacteroides distasonis	Parabacteroides merdae			Chryseobacterium taiwanense
Family	Prevotellaceae																				Rikenellaceae	Tannerellaceae			Weeksellaceae	
Genus	Alloprevotella	Hoylesella				Leyella	Paraprevotella	Prevotella					Segatella								Alistipes	Parabacteroides		Tannerella	Bergeyella	Chryseobacterium
**Oral – Patients on corticosteroids**																										
Li et al. 2016 [49]																										
Sharma et al. 2020 [58]																										
Kim et al. 2022 [47]																										
Van der Meulen et al. 2018 [62]																										
Van der Meulen et al. 2018 [61]																										
Martinez-Nava et al. 2023 [55]																										
**Oral – Patients not on corticosteroids**																										
Alam et al. 2020 [38]					↓				↑	↑		↓				↑										
Zhou et al. 2018 [69]																										
Xie et al. 2024 [67]																										
**Gut – Patients on corticosteroids**																										
Mandl et al. 2017 [54]																										
Van der Meulen et al. 2019 [63]																										
**Gut – Patients not on corticosteroids**																										
Jia et al. 2023 [46]							↓						↓				↓	↓	↓	↓			↑			
Cano-Ortiz et al. 2020 [41]													↑									↓				
Wu et al. 2019 [66]																										
Yang et al. 2022 [68]																										

Species	Campylobacter coli	Campylobacter concisus	Campylobacter gracilis	Campylobacter showae		proportion of patients / abscence prior study	dose
Phylum	Campylobacterota				Cyanobacteriota (Cyanobacteria)		
Family	Campylobacteraceae						
Genus	Campylobacter						
**Oral – Patients on corticosteroids**						proportion of patients	dose
Li et al. 2016 [49]						4/10 pred.	10-20 mg/d
Sharma et al. 2020 [58]						11/37 ster.	N/A
Kim et al. 2022 [47]						4/23 corticost.	N/A
Van der Meulen et al. 2018 [62]						4/23 corticost.	N/A
Van der Meulen et al. 2018 [61]						5/37 corticost.	N/A
Martinez-Nava et al. 2023 [55]						5/45 pred.	N/A
**Oral – Patients not on corticosteroids**						abscence prior study	
Alam et al. 2020 [38]		↓		↓		1 month	
Zhou et al. 2018 [69]						N/A	
Xie et al. 2024 [67]			↑			N/A	
**Gut – Patients on corticosteroids**						proportion of patients	dose
Mandl et al. 2017 [54]						36/42 glucocort.	avg. 5mg/d
Van der Meulen et al. 2019 [63]						2/36 corticost.	N/A
**Gut – Patients not on corticosteroids**						abscence prior study	
Jia et al. 2023 [46]						3 months	
Cano-Ortiz et al. 2020 [41]						3 months	
Wu et al. 2019 [66]						2 months	
Yang et al. 2022 [68]						N/A	

Supplementary Data Sheet 4

Study-level findings of relative abundance of microbes at the species level based on corticosteroid intake of patients

Sjögren's syndrome

Fusobacteriota (Fusobacteria) and Mycoplasmatota (Tenericutes)

Phylum	Fusobacteriota (Fusobacteria)								Mycoplasmatota (Tenericutes)
Family	Fusobacteriaceae				Leptotrichiaceae				Mycoplasmataceae
Genus	Fusobacterium				Leptotrichia			Sneathia	Mycoplasma
Species	Fusobacterium nucleatum	Fusobacterium periodonticum	Fusobacterium ulcerans	Fusobacterium varium	Leptotrichia hofstadii	Leptotrichia hongkongensis	Leptotrichia wadei	Sneathia amnii	Mycoplasma ovis
**Oral – Patients on corticosteroids**									
Li et al. 2016 [49]									
Sharma et al. 2020 [58]									
Kim et al. 2022 [47]									
Van der Meulen et al. 2018 [62]									
Van der Meulen et al. 2018 [61]									
Martinez-Nava et al. 2023 [55]									
**Oral – Patients not on corticosteroids**									
Alam et al. 2020 [38]		↓			↓	↓	↓		
Zhou et al. 2018 [69]									
Xie et al. 2024 [67]		↑							
**Gut – Patients on corticosteroids**									
Mandl et al. 2017 [54]									
Van der Meulen et al. 2019 [63]									
**Gut – Patients not on corticosteroids**									
Jia et al. 2023 [46]									
Cano-Ortiz et al. 2020 [41]									
Wu et al. 2019 [66]									
Yang et al. 2022 [68]									

Pseudomonadota (Proteobacteria), part 1

Family	Acetobacteraceae	Brucellaceae	Burkholderiaceae		Caulobacteraceae	Comamonadaceae			Enterobacteriaceae					Halomonadaceae		Hyphomicrobiaceae	Lysobacteraceae	Moraxellaceae
Genus	Acetobacter	Pseudochrobactrum	Lautropia	Ralstonia	Caulobacter	Delftia	Comamonas	Limnohabitans	Citrobacter	Enterobacter	Escherichia		Klebsiella	Halomonas	Vreelandella			Acinetobacter
Species			Lautropia mirabilis				Comamonas testosteroni		Citrobacter koseri		Escherichia fergusonii	Escherichia coli	Klebsiella pneumoniae		Vreelandella hamiltonii			
**Oral – Patients on corticosteroids**																		
Li et al. 2016 [49]																		
Sharma et al. 2020 [58]																		
Kim et al. 2022 [47]																		
Van der Meulen et al. 2018 [62]																		
Van der Meulen et al. 2018 [61]																		
Martinez-Nava et al. 2023 [55]																		
**Oral – Patients not on corticosteroids**																		
Alam et al. 2020 [38]			↓												↓			
Zhou et al. 2018 [69]																		
Xie et al. 2024 [67]																		
**Gut – Patients on corticosteroids**																		
Mandl et al. 2017 [54]																		
Van der Meulen et al. 2019 [63]																		
**Gut – Patients not on corticosteroids**																		
Jia et al. 2023 [46]													↓					
Cano-Ortiz et al. 2020 [41]											↑							
Wu et al. 2019 [66]																		
Yang et al. 2022 [68]																		

Pseudomonadota (Proteobacteria), part 2

Family	Neisseriaceae						Oxalobacteraceae	Pasteurellaceae						Pseudomonadaceae		Roseobacteraceae	Sphaerotilaceae	Sphingomonadaceae	
Genus	Eikenella	Kingella	Neisseria					Actinobacillus	Haemophilus				Mannheimia	Pseudomonas		Octadecabacter	Roseateles	Novosphingobium	Sphingomonas
Species	Eikenella corrodens		Neisseria elongata	Neisseria oralis	Neisseria perflava	Neisseria subflava			Haemophilus haemolyticus	Haemophilus influenzae	Haemophilus parainfluenzae	Haemophilus sputorum		Pseudomonas aeruginosa	Pseudomonas graminis	Octadecabacter arcticus			
**Oral – Patients on corticosteroids**																			
Li et al. 2016 [49]																			
Sharma et al. 2020 [58]																			
Kim et al. 2022 [47]																			
Van der Meulen et al. 2018 [62]																			
Van der Meulen et al. 2018 [61]																			
Martinez-Nava et al. 2023 [55]																			
**Oral – Patients not on corticosteroids**																			
Alam et al. 2020 [38]	↓		↓	↓	↓	↓			↑		↓	↓							
Zhou et al. 2018 [69]																			
Xie et al. 2024 [67]					↓						↓								
**Gut – Patients on corticosteroids**																			
Mandl et al. 2017 [54]																			
Van der Meulen et al. 2019 [63]																			
**Gut – Patients not on corticosteroids**																			
Jia et al. 2023 [46]																			
Cano-Ortiz et al. 2020 [41]																			
Wu et al. 2019 [66]																			
Yang et al. 2022 [68]																			

Spirochaetota, Synergistota, Thermodesulfobacteria, Verrucomicrobiota

Phylum	Spirochaetota (Spirochaetes)			Synergistota (Synergistetes)		Thermodesulfobacteria			Verrucomicrobiota (Verrucomicrobia)	
Family	Treponemataceae			Aminobacteriaceae	Dethiosulfovibrionaceae	Desulfovibrionaceae			Akkermansiaceae	Verrucomicrobiaceae
Genus	Treponema			Fretibacterium	Pyramidobacter	Bilophila	Desulfovibrio		Akkermansia	
Species	Treponema denticola	Treponema lecithinolyticum	Treponema maltophilum	Fretibacterium fastidiosum	Pyramidobacter piscolens	Bilophila wadsworthia	Desulfovibrio legallii	Desulfovibrio piger	Akkermansia muciniphila	
**Oral – Patients on corticosteroids**										
Li et al. 2016 [49]										
Sharma et al. 2020 [58]										
Kim et al. 2022 [47]										
Van der Meulen et al. 2018 [62]										
Van der Meulen et al. 2018 [61]										
Martinez-Nava et al. 2023 [55]										
**Oral – Patients not on corticosteroids**										
Alam et al. 2020 [38]	↓									
Zhou et al. 2018 [69]										
Xie et al. 2024 [67]										
**Gut – Patients on corticosteroids**										
Mandl et al. 2017 [54]										
Van der Meulen et al. 2019 [63]										
**Gut – Patients not on corticosteroids**										
Jia et al. 2023 [46]						↓				
Cano-Ortiz et al. 2020 [41]										
Wu et al. 2019 [66]										
Yang et al. 2022 [68]										

	proportion of patients / absconce prior study	dose
**Oral – Patients on corticosteroids**	**proportion of patients**	**dose**
Li et al. 2016 [49]	4/10 pred.	10-20 mg/d
Sharma et al. 2020 [58]	11/37 ster.	N/A
Kim et al. 2022 [47]	4/23 corticost.	N/A
Van der Meulen et al. 2018 [62]	4/23 corticost.	N/A
Van der Meulen et al. 2018 [61]	5/37 corticost.	N/A
Martinez-Nava et al. 2023 [55]	5/45 pred.	N/A
**Oral – Patients not on corticosteroids**	**abscence prior study**	
Alam et al. 2020 [38]	1 month	
Zhou et al. 2018 [69]	N/A	
Xie et al. 2024 [67]	N/A	
**Gut – Patients on corticosteroids**	**proportion of patients**	**dose**
Mandl et al. 2017 [54]	36/42 glucocort.	avg. 5mg/d
Van der Meulen et al. 2019 [63]	2/36 corticost.	N/A
**Gut – Patients not on corticosteroids**	**abscence prior study**	
Jia et al. 2023 [46]	3 months	
Cano-Ortiz et al. 2020 [41]	3 months	
Wu et al. 2019 [66]	2 months	
Yang et al. 2022 [68]	N/A	
